# Comparative genomics and genome-wide SNPs of endangered Eld’s deer provide breeder selection for inbreeding avoidance

**DOI:** 10.1038/s41598-023-47014-x

**Published:** 2023-11-13

**Authors:** Vichayanee Pumpitakkul, Wanna Chetruengchai, Chalurmpon Srichomthong, Chureerat Phokaew, Wirulda Pootakham, Chutima Sonthirod, Wanapinun Nawae, Sissades Tongsima, Pongsakorn Wangkumhang, Alisa Wilantho, Yongchai Utara, Ampika Thongpakdee, Saowaphang Sanannu, Umaporn Maikaew, Suphattharaphonnaphan Khuntawee, Wirongrong Changpetch, Phairot Phromwat, Kacharin Raschasin, Phunyaphat Sarnkhaeveerakul, Pannawat Supapannachart, Wannapol Buthasane, Budhan S. Pukazhenthi, Klaus-Peter Koepfli, Prapat Suriyaphol, Sithichoke Tangphatsornruang, Gunnaporn Suriyaphol, Vorasuk Shotelersuk

**Affiliations:** 1 Biochemistry Unit, Department of Physiology, Faculty of Veterinary Science, Chulalongkorn University, Bangkok, 10330 Thailand; 2 Center of Excellence for Medical Genomics, Medical Genomics Cluster, Department of Pediatrics, Faculty of Medicine, Chulalongkorn University, Bangkok, 10330 Thailand; 3 Excellence Center for Genomics and Precision Medicine, King Chulalongkorn Memorial Hospital, The Thai Red Cross Society, Bangkok, 10330 Thailand; 4 National Center for Genetic Engineering and Biotechnology, National Science and Technology Development Agency, Pathum Thani, 12120 Thailand; 5 Zoological Park Organization of Thailand, Animal Conservation and Research Institute, Bangkok, 10800 Thailand; 6 Khao Kheow Open Zoo, Zoological Park Organization of Thailand, Chonburi, 20110 Thailand; 7 Ubon Ratchathani Zoo, Zoological Park Organization of Thailand, Ubon Ratchathani District, Ubon Ratchathani, 34000 Thailand; 8 Nakhon Ratchasima Zoo, Zoological Park Organization of Thailand, Nakhon Ratchasima, 30000 Thailand; 9 Huai Kha Khaeng Wildlife Breeding Center, Department of National Parks, Wildlife and Plant Conservation, Uthai Thani, 61160 Thailand; 10 Chulabhorn Wildlife Breeding Center, Department of National Parks, Wildlife and Plant Conservation, Sisaket, 33140 Thailand; 11 Banglamung Wildlife Breeding Center, Department of National Parks, Wildlife and Plant Conservation, Chonburi, 20150 Thailand; 12 Center for Species Survival, Smithsonian Conservation Biology Institute, National Zoological Park, Front Royal, VA 22630 USA; 13 Smithsonian-Mason School of Conservation, George Mason University, Front Royal, VA 22630 USA; 14 Office for Research and Development, Faculty of Medicine, Siriraj Hospital, Mahidol University, Bangkok, 10700 Thailand

**Keywords:** Evolutionary genetics, DNA sequencing, Next-generation sequencing, Genome, Genomics, Inbreeding, Evolution, Genetics, Molecular biology, Systems biology, Zoology

## Abstract

Eld’s deer, a conserved wildlife species of Thailand, is facing inbreeding depression, particularly in the captive Siamese Eld’s deer (SED) subspecies. In this study, we constructed genomes of a male SED and a male Burmese Eld’s deer (BED), and used genome-wide single nucleotide polymorphisms to evaluate the genetic purity and the inbreeding status of 35 SED and 49 BED with limited pedigree information. The results show that these subspecies diverged approximately 1.26 million years ago. All SED were found to be purebred. A low proportion of admixed SED genetic material was observed in some BED individuals. Six potential breeders from male SED with no genetic relation to any female SED and three purebred male BED with no relation to more than 10 purebred female BED were identified. This study provides valuable insights about Eld’s deer populations and appropriate breeder selection in efforts to repopulate this endangered species while avoiding inbreeding.

## Introduction

Eld’s deer, also known as brow-antlered deer (*Rucervus eldii*), is a tropical medium-sized cervid distributed across the lowland forests of South Asia and Southeast Asia. Eld’s deer plays an important role in maintaining ecological balance by dispersing plant seeds and serving as prey for top predators in the food chain^1,2^. The unique features of Eld’s deer are its bow-like antlers and long, slender body with thin, long legs. However, owing to overexploitation of natural resources, illegal trade and habitat destruction, the population of Eld’s deer has critically declined and is at high risk of extinction worldwide. As a result, Eld’s deer is listed as endangered (EN) on the Red List of Threatened Species by the International Union for Conservation of Nature (IUCN) and is also included in Appendix I of the Convention on International Trade in Endangered Species of Wild Fauna and Flora (CITES)^3,4^. In Thailand, Eld’s deer is also classified as conserved wild animal in Wild Animal Conservation and Protection Act, B.E. 2562 (2019)^5^.

The taxonomic status of Eld’s deer is still debated. Some studies propose that Eld’s deer belongs to either the genus *Cervus* or *Panolia*, based on mitochondrial DNA information^6,7^. Other studies report that this cervid shares a general morphological appearance with the extinct Schomburgk’s deer (*Rucervus schomburgki*) and the barasingha (*Rucervus duvaucelii*), and therefore, it is classified in the genus *Rucervus*, which is also currently adopted by the IUCN^8,9^. Three subspecies of Eld’s deer have been classified: Siamese Eld’s deer (*R. e. siamensis*, SED), Burmese Eld’s deer or thamin (*R. e. thamin*, BED) and Manipur Eld’s deer or sangai (*R. e. eldii*)^4^. The isolated population that resides on Hainan Island of China is still controversial and is proposed either as a fourth subspecies or conspecific with the SED subspecies^4,10^.

Thailand is considered a significant historical center of Eld’s deer due to the location and discovery of the oldest fossils from the Middle and Late Pleistocene in the Northeastern and Northern regions. Several fossils were found in the caves of Kao Pah Nam, Tham Wiman Nakin and Khok Sung sand pit^11,12^. The co-occurrence of Eld’s deer and sambar deer fossils dating to the Late Pleistocene were found at the Chiang Dao Wildlife Sanctuary^13^. Two subspecies of Eld’s deer, SED and BED, currently exist in Thailand, and Y chromosome analysis has been used to distinguish the genetic divergence between these subspecies^14^. The population of Eld’s deer is facing the threat of extinction due to severe poaching and a small founder size, which affects the remaining population and genetic diversity level^15^. The captive SED lineage in Thailand is believed to have originated from only three known founders: a pair of breeders in 1984 and one additional male in 2003. Low fertility rates, high neonatal mortality and juvenile death have been observed^15–17^, suggesting inbreeding depression in this lineage. Regarding the BED situation, one male and one female fertile hybrid offspring (F1 generation) were bred from a male BED and female SED founder, and the fertile hybrid F2 generation, obtained in 1985, was reintroduced to mate with other wild BED at the Department of National Parks, Wildlife and Plant Conservation (DNP) for several generations^15,16^. In 1983, the Zoological Park Organization of Thailand (ZPOT) obtained eleven BED founders. After that, outbred BEDs were introduced from various sources, including local organizations and from abroad. The captive breeding of this subspecies has been successful, and many BED fawns have been produced for reintroduction to the wild in Thailand. Further examination of the proportion of admixed SED in the BED population, or vice versa, and the analysis of inbreeding parameters in both subspecies can help to select genetically unrelated and purebred breeders for repopulation in the wild.

Genome-wide single nucleotide polymorphisms (SNPs) can be useful for discovering the pattern of genetic diversity and population structure^18^. Unlike mitochondrial DNA and microsatellites, genome-wide SNPs provide a large number of molecular markers that can be generated with cost-effective methods and low running time^19^. Restriction site-associated DNA sequencing (RADseq) is a promising technique for identifying genome-wide SNPs by reducing the complexity of the target genome^20^. This technique has been used to demonstrate individual-level genotype information of various wildlife, particularly endangered species, such as hog deer (*Axis porcinus*), forest musk deer (*Moschus berezovskii*), Raso lark (*Alauda razae*) and Dahl's toad-headed turtle (*Mesoclemmys dahli*)^21–24^. Additionally, RADseq has been applied to demonstrate hybridization in wild Bactrian camels (*Camelus ferus*)^25^. However, detection of genome-wide SNPs has not yet been implemented in Eld’s deer. A comprehensive analysis of genome-wide genetic diversity could help to avoid inbreeding in populations and aid in organizing further breeding strategies for the Eld’s deer population in Thailand.

In the present study, we performed de novo whole-genome sequencing (WGS) to construct reference genomes and the mitogenomes of a male SED and a male BED. We also examined gene family expansion and contraction, analyzed gene family enrichment, identified orthologous gene families specific to SED and BED, and detected genes under positive selection, in comparison to other species. According to genome-wide SNPs obtained through RADseq, we estimated the demographic history, population structure and runs of homozygosity (ROH), and assessed the genetic purity and relatedness of each SED and BED individual in the captive populations of these subspecies. These findings are crucial for addressing the occurrence of close breeding and avoiding inbreeding dilemmas in this endangered species. Our study emphasizes the genome biology of both subspecies of Eld’s deer and delivers valuable information to promote a well-designed breeding plan and appropriate selection of mating pairs in support of the conservation efforts for this endangered species.

## Results

### De novo genome assemblies and genome annotation

We assembled a de novo genome of a seven-year-old male SED from Ubon Ratchathani Zoo using a combination of Illumina short-reads (92.94 × coverage) and PacBio long-reads (61.6 × coverage) (GenBank accession number: JACCHN000000000). Additionally, we used MGI short-reads (52.15 × coverage) to assemble a de novo genome of a 10-year-old male BED from the Khao Kheow Open Zoo (GenBank accession number: JAJHSM000000000) (see Methods). The genome assembly metrics, GC content, Benchmarking Universal Single-Copy Orthologs (BUSCO) gene completeness scores, repetitive sequences, annotation of protein-coding genes and non-coding RNAs and KEGG categories of putative genes of the SED and BED assemblies are shown in Table 1, Supplementary Tables 1–4, and Supplementary Fig. 1. The completeness of the SED and BED genomes were 92.4% and 90% for the complete single-copy and duplicated BUSCOs of the 9226 single-copy orthologs in the mammalia_odb10 dataset, respectively. The total lengths of the SED and BED genomes are 2.48 Gb and 2.32 Gb, the number of protein-coding genes are 24,913 and 28,831, and the GC content is 41.58% and 41.64%, respectively.

**Table 1:** Statistics of the Siamese (SED) and Burmese (BED) Eld’s deer genomes.

	SED			BED	
	PacBio long-read assembly	Illumina short-read assembly	SSPACE-LongRead hybrid assembly + PacBio contigs	MGI short-read assembly	RagTag scaffolding
N50 contig/scaffold size (bases)	21,876,177	14,321	24,616,240	10,634	23,319,789
L50 contig/scaffold number	33	46,339	28	70,177	27
Assembly size (bases)	2,712,019,647	1,178,002	2,484,241,219	2,838,198,300	2,324,991,452
Number of contigs/scaffolds	2552	360,490	284	3,509,301	12,762
Number of contigs/scaffolds ≥ 1 Kbp	2552	284,266	284	272,907	12,756
Number of contigs/scaffolds ≥ 10 Kbp	2535	74,107	279	0	1202
Number of contigs/scaffolds ≥ 10 Mbp	0	0	77	0	68
Longest contig/scaffold (bases)	69,907,081	242,553	69,955,205	155,293	65,698,769
Number of Ns	0	0	17,942,519	2,957,114	28,963,822
GC content (%)	41.6	41.57	41.58	43	41.64
BUSCO evaluation (% completeness)			92.4		90

### Mitogenome assemblies and comparative mitogenome analysis

**Figure 1:**
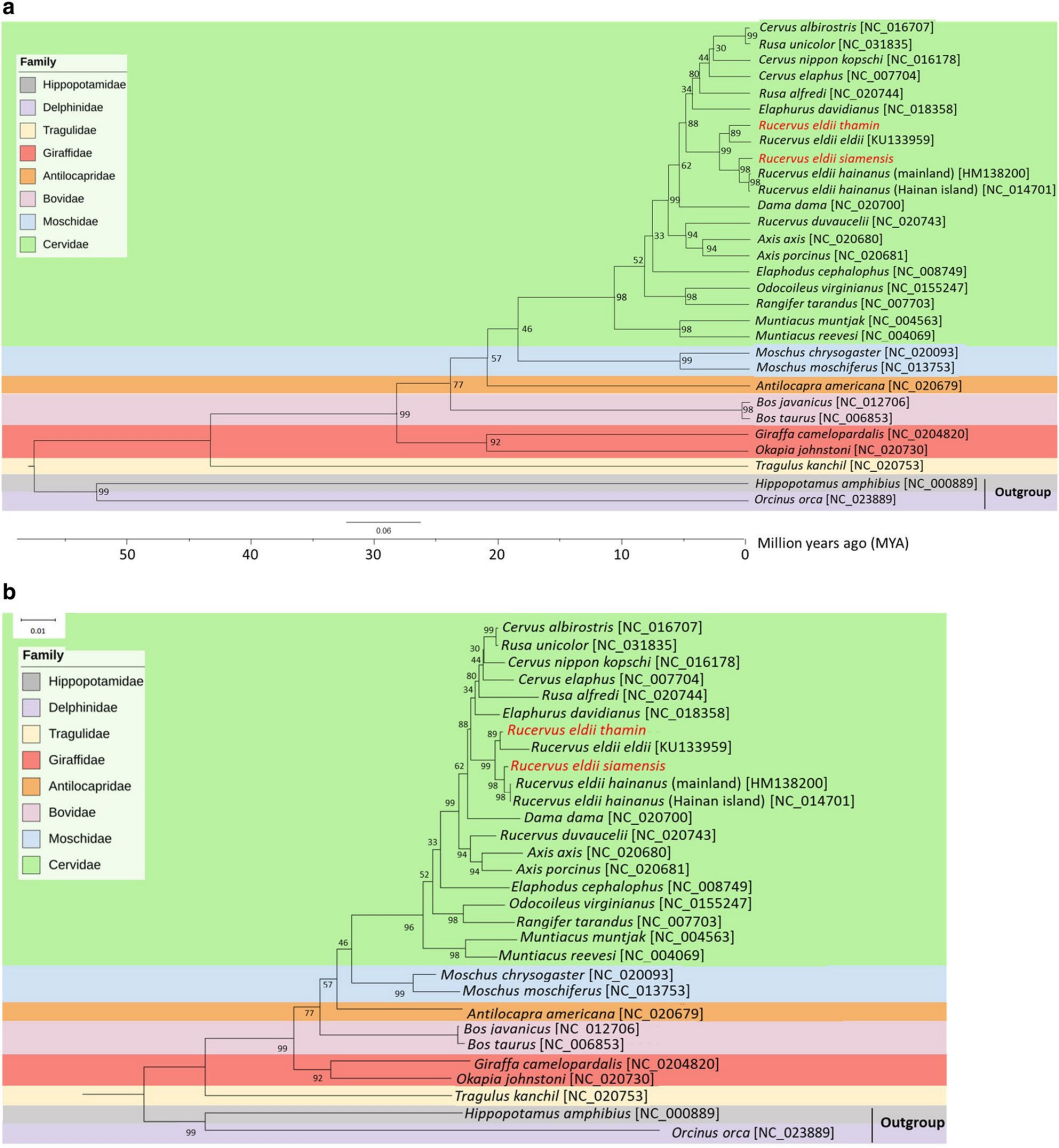
Maximum-likelihood phylogenetic tree based on the amino acid alignments of 13 conserved protein-coding genes in the mitochondrial genomes of *Rucervus eldii siamensis*, *Rucervus eldii thamin* and 28 other Cetartiodactyla species and using *Hippopotamus amphibius* and *Orcinus orca* as outgroups. (**a**) Cladogram. (**b**) Phylogram. The number at each node represents bootstrap values of 1000 replicates. The analysis includes 3805 amino acid characters and 591 parsimony-informative sites. The tree was estimated using MEGA X and visualized using the Interactive Tree Of Life (iTOL; v6). GenBank accession numbers of all selected species are indicated with their species names. *Rucervus eldii siamensis* (SED) and *Rucervus eldii thamin* (BED) are in red.

The sizes of the complete mitogenomes of SED and BED were 16,357 and 16,358 bp, respectively (GenBank accession numbers: OP205647 and OP235941). Both mitogenomes had a GC content of 38.2%. They consisted of 13 protein-coding genes, 22 tRNA genes, two rRNA genes, and the D-loop region. The circular structures and annotation of the complete mitochondrial genomes of SED and BED are illustrated in Supplementary Fig. 2 and Supplementary Table 5. The mitogenomes of SED and BED were aligned with 28 previously published mitogenomes of Cetartiodactyla species (even-toed ungulates) and visualized as a maximum likelihood phylogeny. The mitogenomes of hippopotamus (*Hippopotamus amphibius*) and orca (*Orcinus orca*) were used as outgroups. The SED and BED mitogenomes were closely related to those of Hainan Eld's deer (*Rucervus eldii hainanus*) and sangai (*Rucervus eldii eldii*), respectively. Additionally, the mitogenome of Père David’s deer (*Elaphurus davidianus*) was found to be more closely related to *Rucervus* spp. compared with *Cervus* spp. and *Rusa* spp. (Fig. 1). Pairwise genetic distances were computed among all Eld’s deer subspecies sequences, and remarkably low genetic divergence was observed compared to the divergence observed at the interspecies level. Specifically, the genetic distance between SED and BED was 0.0053, and the genetic distances between SED and Hainan Eld’s deer, and between BED and sangai were 0.0026 and 0.0084, respectively (Supplementary Table 6).

### Comparative genomics, specific orthologous gene families and expanded/contracted gene families

**Figure 2:**
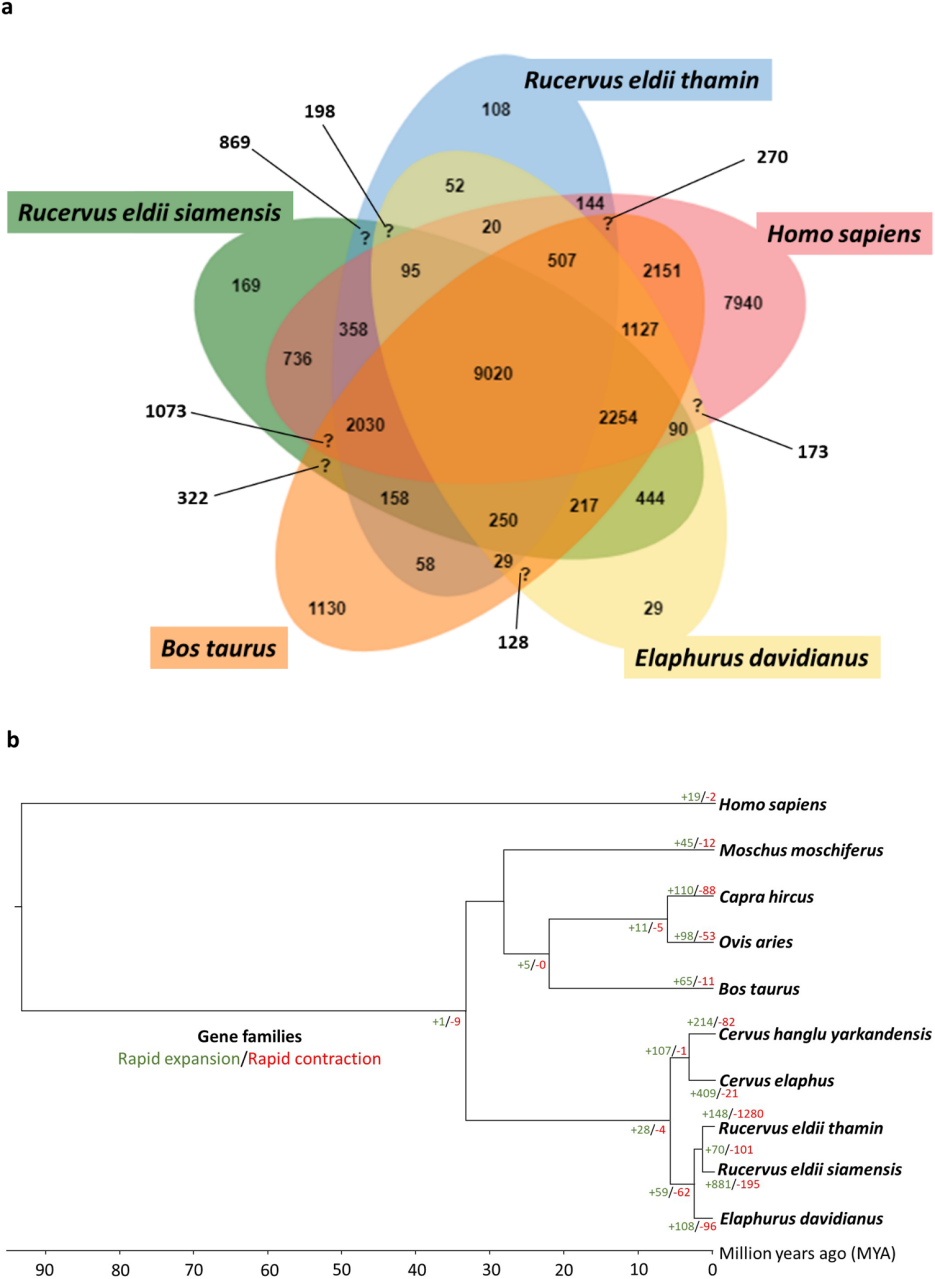
Comparative genomic analysis of *Rucervus eldii siamensis* (SED), *Rucervus eldii thamin* (BED) and three other mammalian species. (**a**) Venn diagram of the gene clusters shared among SED, BED, *Elaphurus davidianus* (Père David’s deer), *Bos taurus* (cattle) and *Homo sapiens* (human) using OrthoVenn2, (**b**) Phylogenetic tree showing divergence times and the evolution of gene family sizes of SED, BED and the genomes of eight other species. The tree was constructed based on single-copy orthologs to show divergence times, computed by MCMCTree in the PAML v4.9j package. *Homo sapiens* was included as an outgroup. The numbers of expanded (green) and contracted (red) gene families are shown at the nodes in the phylogenetic tree and were determined using CAFE v4.2.1.

We compared our SED and BED genomes to eight different mammalian species for the analysis of comparative genomics (Supplementary Table 7). To determine the species-specific gene clusters, we initially selected protein-coding sequences of five species, including *Homo sapiens*, *Bos taurus* and *Elaphurus davidianus,* along with *Rucervus eldii siamensis* and *Rucervus eldii thamin*. A total of 32,149 clusters were formed, of which 30,416 were orthologous clusters (containing at least two species) and 1733 were single-copy gene clusters. Results showed that 9020 gene families were shared among the five species, as illustrated by the Venn diagram in Fig. 2a. A total of 169 species-specific gene families were found in SED, and 125 gene clusters were annotated in the Swiss-Prot database. Functional enrichment analysis of gene pathways showed that genes related to the translation process (GO:0006412; GO level: biological process, *p* = 6.79E−13), sensory perception of smell (GO:0007608; GO level: biological process, *p* = 9.72E−06), and virion assembly (GO:0019068; GO level: biological process, *p* = 1.12E−14) comprised the unique gene clusters in SED (Supplementary Tables 8 and 9). For BED, a total of 108 species-specific gene families were found, and 49 gene clusters were annotated. The pathways related to transposition and RNA-mediated (GO:0032197; GO level: biological process, *p* = 1.40E−26) and DNA recombination (GO:0006310; GO level: biological process, *p* = 1.24E−14) were significantly enriched in BED (Supplementary Tables 10 and 11).

We further analyzed the phylogenetic tree and expansion and contraction of gene families among SED, BED and eight other species. The human (*Homo sapiens*) genome was used as the outgroup. The evolutionary divergence time was estimated from nuclear and mitochondrial information. The analysis of nuclear protein sequences revealed a divergence time of approximately 1.26 million years ago (Mya) (95% confidence interval (CI) 0.64–1.94 Mya) between SED and BED (Fig. 2b, Supplementary Fig. 3a). In comparison, the examination of mitochondrial data showed an estimated divergence time of approximately 2.47 Mya (95% CI 1.54–3.46 Mya) between SED and Hainan Eld’s deer clade and BED and sangai clade (Fig. 1, Supplementary Fig. 3b). Additionally, the divergence time between Père David's deer and Eld's deer was estimated to be approximately 2.34 Mya (95% CI 1.98–2.57 Mya), regarding the nuclear data (Fig. 2b, Supplementary Fig. 3a). Significant gene family expansions and contractions were identified in several clades, including within the Cervidae clade, the Père David’s deer-BED-SED clade, the BED-SED clade, and the individual BED and SED lineages. The functional significance of these gene family changes was determined through Gene Ontology (GO) enrichment analysis. The SED genome showed 1716 expanded gene families and 971 contracted gene families, with 881 expanded and 195 contracted gene families significant at *p* < 0.01. Meanwhile, the BED genome showed 275 expanded and 5019 contracted gene families, with 148 expanded and 1280 contracted gene families significant at *p* < 0.01 (Fig. 2b). Genes related to 60S ribosomal protein were the most expanded gene families in the Cervidae clade, corresponding to the significant GO enrichment pathways of translation (GO: 0006421; GO level: biological process, *p* = 2.8E−05), cytoplasmic translation (GO: 0002181; GO level: biological process, *p* = 5.2E−05) and RNA binding (GO: 0003723; GO level: molecular function, *p* = 4.4E-03). The expanded gene families specifically found in the Cervidae clade included M-phase phosphoprotein 6 (MPHOSPH6) which corresponds to the RNA binding process. Interestingly, the gene families involved in central nervous system development (GO: 0007417; GO level: biological process) and axon guidance (GO: 0007411; GO level: biological process) were expanded in the Père David’s deer-BED-SED clade, such as Down syndrome cell adhesion molecule-like protein 1 (DSCAML1). Groups of olfactory receptors (ORs) were predominantly found among the top expanded gene families of the BED-SED group, associated with the significantly enriched pathways of detection of chemical stimuli involved in sensory perception of smell (GO: 0050911; GO level: biological process, *p* = 7.7E−09), G-protein-coupled receptor signaling pathway (GO:0007186; GO level: biological process, *p* = 1E−05) and OR activity (GO: 0004984; GO level: molecular function, *p* = 2.3E−08). The expanded gene families detected only in the BED-SED group included receptor-type tyrosine-protein phosphatase T (PTPRT) and tyrosine-protein kinase RYK isoform X7 (RYK). Both groups were enriched in the transmembrane receptor protein tyrosine kinase signaling pathway (GO: 0007169; GO level: molecular function). For SED, large (RPLs) and small subunit ribosomal proteins (RPSs), ORs and genes involved in protein binding were among the top expanded gene families. The significantly enriched functions involved cytoplasmic translation (RPL and RPS families) (GO: 0002181; GO level: biological process, *p* = 1.1E−39) and detection of chemical stimuli involved in sensory perception of smell (OR families) (GO: 0050911; GO level: biological process, *p* = 3E−28), G-protein-coupled receptor activity (OR families) (GO: 0004930; GO level: molecular function, *p* = 3.1E−15) and identical protein binding (protein binding gene families) (GO:0042802; GO level: molecular function, *p* = 0.014). In the BED lineage, groups of gene families related to retroviral integration were mostly observed, such as NYN domain and retroviral integrase containing proteins (NYNRIN) and endogenous retrovirus group K proteins (ERVKs). The significantly enriched GO pathways related to those gene families were DNA integration (GO: 0015074; GO level: biological process, *p* = 4.6E−07) and RNA–DNA hybrid ribonuclease activity (GO: 0004523; GO level: molecular function; *p* = 5.1E−05). Also, groups of ORs were considered, which are related to the significantly enriched GO functions of detection of chemical stimuli involved in sensory perception of smell (GO: 0050911; GO level: biological process, *p* = 7.3E−07) and G-protein-coupled receptor activity (GO: 0004930; GO level: molecular function, *p* = 1.2E−05). Furthermore, the GO pathway of metal ion binding was found to be significant (GO: 0046872; GO level: molecular function, *p* = 0.036).

### Detection of positively selected genes (PSGs)

Within the Cervidae clade, single-copy orthologous proteins of SED, BED and three other cervids (*Cervus elaphus*, *Cervus hanglu yarkandensis* and *Elaphurus davidianus*) were compared. The results showed that there were 17, 10, and 12 PSGs in the BED-SED clade, SED lineage and BED lineage, respectively (Supplementary Table 12). Most of the PSGs found in both SED and BED were related to positive regulation of T-cell-mediated cytotoxicity in the GO term enrichment (GO:0001916; GO level: biological process, *p* = 0.02). The genes that showed positive selection specifically in SED displayed a significant enrichment of pathways for the regulation of transcription from RNA polymerase II promoter (GO: 0006357; GO level: biological process, *p* = 0.03) and transcription factor activity and sequence-specific DNA binding (GO: 0003700; GO level: molecular function; *p* < 0.03) (Supplementary Table 13).

### RADseq analysis and SNP calling

**Figure 3:**
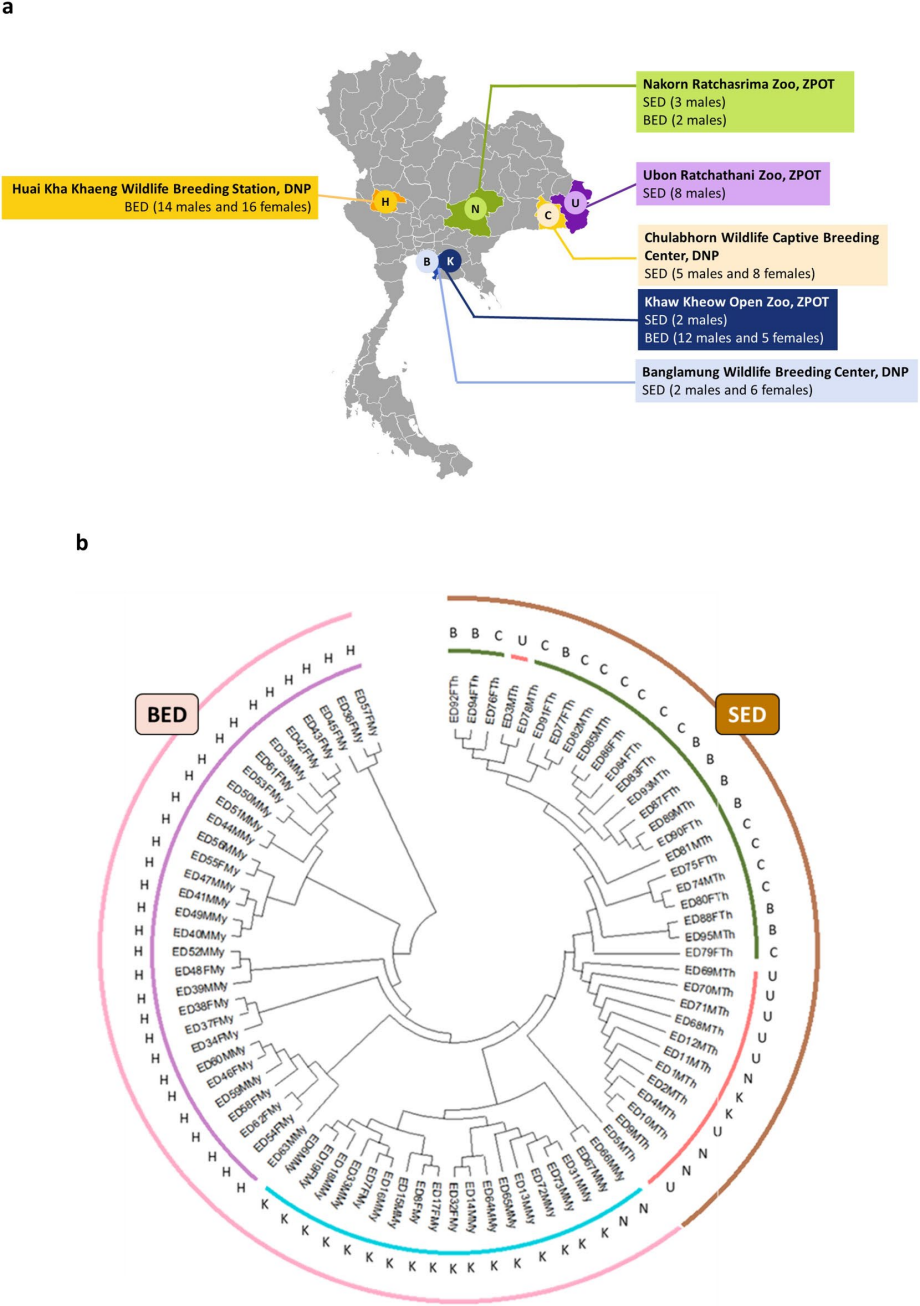
Sample collection site and phylogenetic relationship of 84 Eld’s deer individuals. (**a**) Map of Thailand indicating the locations of sample collection. (**b**) Maximum-likelihood phylogenetic tree of 84 individuals of Siamese Eld’s deer (SED) and Burmese Eld’s deer (BED) populations with 1000 bootstrap replications. Sampling sites: B, Banglamung Wildlife Breeding Center, Department of National Parks Wildlife and Plant Conservation (DNP); C, Chulabhorn Wildlife Breeding Center, DNP; H, Huai Kha Khaeng Wildlife Breeding Center, DNP; K, Khao Kheow Open Zoo, Zoological Park Organization of Thailand (ZPOT), N, Nakorn Ratchasima Zoo, ZPOT; U, Ubon Ratchathani Zoo, ZPOT.

We additionally sequenced and genotyped 84 Eld’s deer individuals (SED = 35, BED = 49) using the RADseq technique. Samples were received from two main organizations in Thailand, ZPOT and DNP, and were classified into six locations (Fig. 3a, Supplementary Table 14). We obtained a total of 2.09 billion cleaned paired-end reads (308.9 Gb) across the 84 individuals and mapped them against our SED genome assembly. The number of aligned reads per individual ranged from 7.63 to 120 million, with an average of 23.94 million reads, accounting for 96%. The total numbers of paired-end and mapped reads, and percentage of overall alignment rates per individual, are shown in Supplementary Table 15. A total of 1,726,048 SNPs were called and used for further downstream analysis.

### RADseq-based population structure

After initially obtaining 1,726,048 candidate SNP loci from the 84 individuals, we subsequently filtered these SNPs and a total of 3067 filtered SNPs were retained. These SNPs were used for constructing a maximum-likelihood phylogenetic tree of the SED and BED populations with 1000 bootstrap replications (see Methods). Individuals of SED and BED were mostly clustered according to subspecies, but were also clustered based on locations (ZPOT and DNP). We also noticed that most individuals that belonged to the same organization were grouped in the same clade, indicating the close genetic relationship within the organization (Fig. 3b).

**Figure 4:**
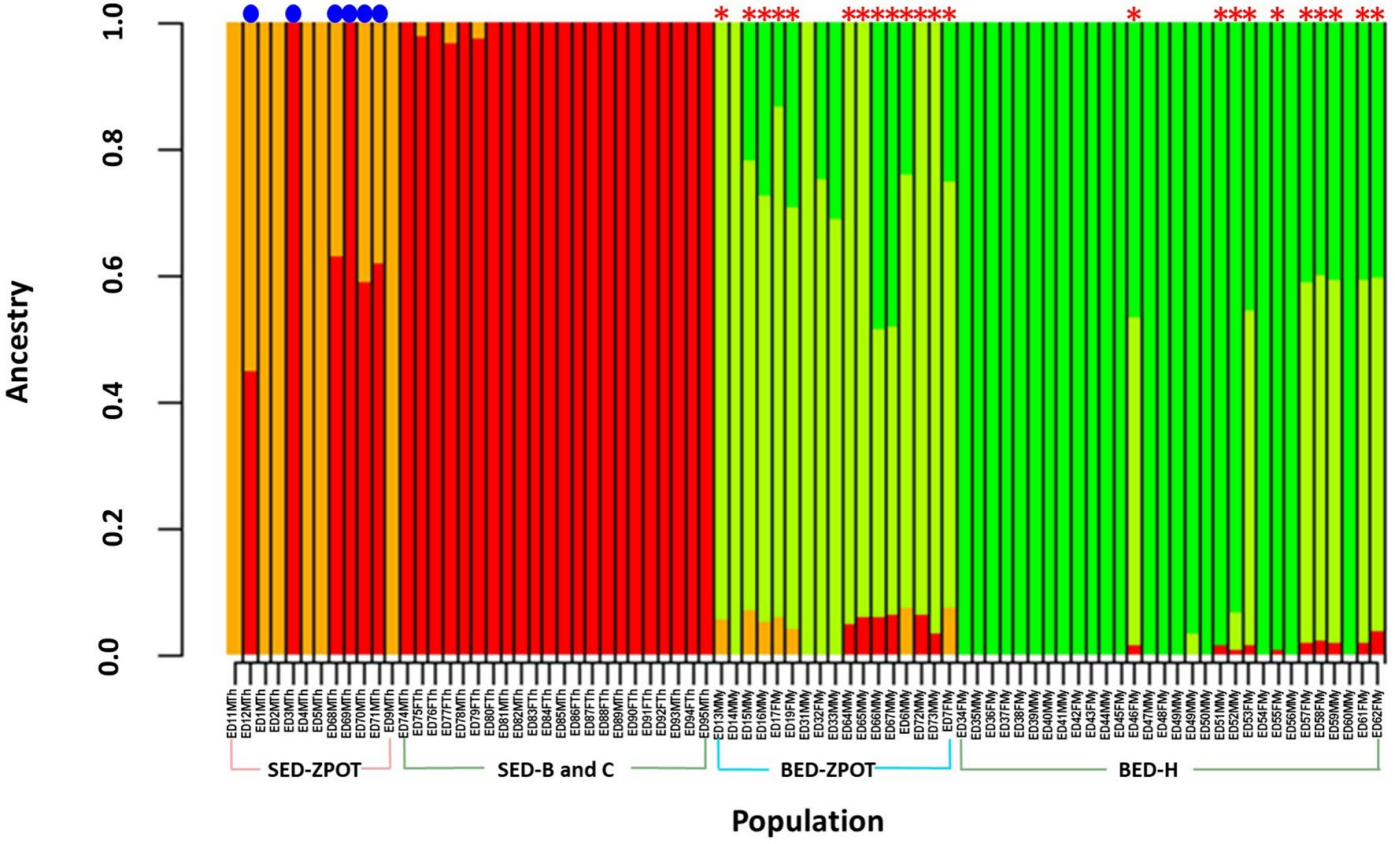
ADMIXTURE analysis plot for *K* = 4 clusters with 81 individuals and 33,708 single nucleotide polymorphism loci. Each vertical bar represents one sample and the proportion determines the estimated genetic ancestry per individual. The vertical axis shows individual ancestry according to sampling populations (range: 0–1). Sampling sites: SED-ZPOT, Siamese Eld’s deer of the Zoological Park Organization of Thailand; SED-DNP, Siamese Eld’s deer of the Department of National Parks, Wildlife and Plant Conservation; BED-ZPOT, Burmese Eld’s deer of the Zoological Park Organization of Thailand; BED-DNP, Burmese Eld’s deer of the Department of National Parks, Wildlife and Plant Conservation. Blue dots indicate SED-ZPOT with > 40% admixture proportion of SED-DNP genetic ancestry. Red asterisks indicate BED with SED genetic ancestry, suggesting past admixture.

We used PLINK v1.9^26^ to filter the 1,726,048 SNP sites. Two SED (ED8FTh and ED10MTh) individuals and one BED (ED18MMy) individual were removed because of the high number of missing genotypes. A total of 273,187 SNP sites were obtained across the 81 individuals for further linkage disequilibrium (LD)-based SNP pruning. Following this, 33,708 SNP sites were acquired for the estimation of individual genetic ancestries using the ADMIXTURE tool (see Methods). The best *K*-value for the whole population was determined from the cross-validation (CV) error plot for *K*-values from 1 to 20. ADMIXTURE analysis demonstrated that *K* = 4 showed the lowest cross-validation error (Supplementary Figs. 4 and 5). Hence, the Eld’s deer samples in the present study can be divided into four subpopulations in accordance with the Eld’s deer subspecies and sampling site organization, including Siamese Eld’s deer from the Zoological Park Organization of Thailand (SED-ZPOT) and from the Department of National Parks, Wildlife and Plant Conservation (SED-DNP), and Burmese Eld’s deer from the Zoological Park Organization of Thailand (BED-ZPOT) and from the Department of National Parks, Wildlife and Plant Conservation (BED-DNP). All males and females in the SED subpopulation exhibited 100% SED genetic ancestry, suggesting that these are purebred animals. However, two male SED-ZPOT individuals (ED3MTh and ED69MTh) exhibited 100% ancestry with the SED-DNP lineage. In addition, four male SED-ZPOT individuals (ED12MTh, ED68MTh, ED70MTh and ED71MTh) had > 40% admixture proportion of SED-DNP genetic ancestry (Fig. 4). In contrast, the BED subpopulation, particularly at the ZPOT, showed a low admixture proportion of the SED lineage, reflecting admixture of the SED lineage into the BED subpopulation. However, we ultimately inferred 24 purebred BED, including 12 males and 12 females (Fig. 4).

### Analysis of genomic diversity and inbreeding parameters

The 33,708 filtered and LD-pruned SNP sites from the previous analysis were subsequently used for the assessment of genetic diversity and inbreeding values of the Eld’s deer populations. The genetic diversity parameters of the four subpopulations were calculated as shown in Table 2 and Supplementary Table 16. Remarkably, the genetic diversities of the subpopulations were significantly different. The highest average heterozygosity and the lowest average genomic inbreeding coefficient were detected in the BED-ZPOT subpopulation and were 0.27 ± 0.04 and 0.03 ± 0.14, respectively. High genomic inbreeding coefficients, combined with low heterozygosity in both SED subpopulations, implied close relatedness and mating among siblings. Among the SED subpopulations, positive values of the inbreeding coefficient were found in all individuals. The SED-DNP subpopulation had a lower average heterozygosity (0.16 ± 0.02 in SED-DNP and 0.18 ± 0.05 in SED-ZPOT) and higher genomic inbreeding coefficient values (0.45 ± 0.08 in SED-DNP and 0.37 ± 0.16 in SED-ZPOT) compared to the SED-ZPOT group. By contrast, negative inbreeding coefficients were also detected in some BED individuals, indicating an excess of heterozygosity. Additionally, the BED-DNP subpopulation had lower heterozygosity than the BED-ZPOT group.

**Table 2:** Summary statistics of genetic diversity parameters of four subpopulations of Eld’s deer.

Subpopulation	Sample size	Heterozygosity rate (Mean ± SD)	Genomic inbreeding coefficient (Mean ± SD)	Genomic inbreeding coefficient (Min)	Genomic inbreeding coefficient (Max)
SED-ZPOT	12	0.18 ± 0.05^a^^, e^	0.37 ± 0.16^a, e^	0.05	0.63
SED-DNP	22	0.16 ± 0.02^b, g^	0.45 ± 0.08^b, g^	0.23	0.61
BED-ZPOT	17	0.27 ± 0.04^c, f^	0.03 ± 0.14^c, f^	− 0.23	0.31
BED-DNP	30	0.25 ± 0.02^d, f, h^	0.16 ± 0.13^d, f, h^	− 0.002	0.63

^a, b^Denotes a significant difference in the same column at *p* < 0.05.

^c, d^Denotes a significant difference in the same column at *p* < 0.05.

^e, f^Denotes a significant difference in the same column at *p* < 0.001.

^g, h^Denotes a significant difference in the same column at *p* < 0.001.

Sampling sites: SED-ZPOT, Siamese Eld’s deer of the Zoological Park Organization of Thailand; SED-DNP, Siamese Eld’s deer of the Department of National Parks, Wildlife and Plant Conservation; BED-ZPOT, Burmese Eld’s deer of the Zoological Park Organization of Thailand; BED-DNP, Burmese Eld’s deer of the Department of National Parks, Wildlife and Plant Conservation.

**Figure 5:**
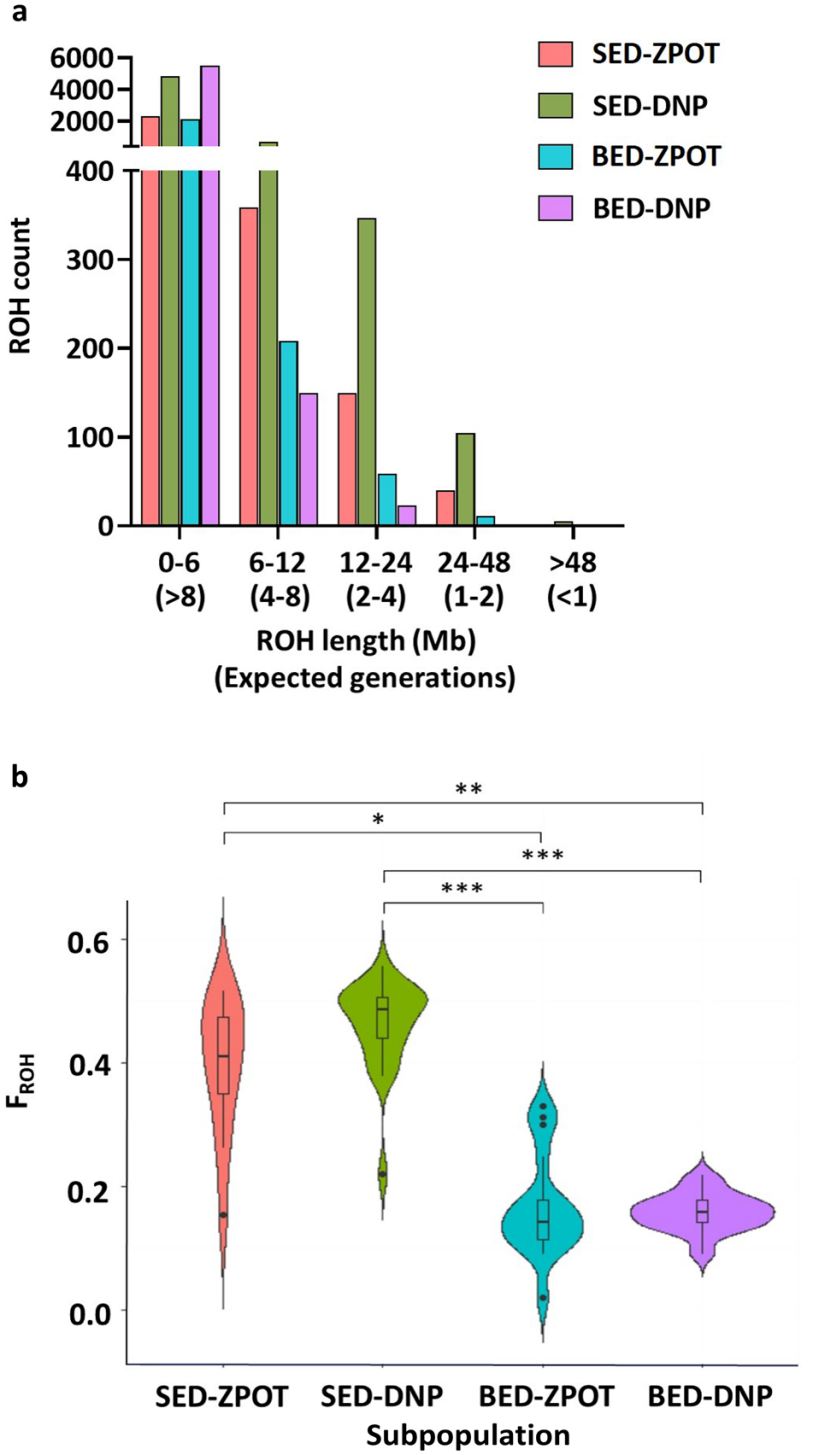
Runs of homozygosity (ROH) analysis of Eld’s deer, calculated using PLINK v1.9 and the detectRUNS package. (**a**) Distribution of ROH in four Eld’s deer subpopulations across length classes. ROH length in Mb and expected number of generations that their lineages shared a common ancestor are indicated on the horizontal axis. ROH count is shown on the vertical axis. The bar graph was plotted using GraphPad Prism. (**b**) Violin plot of genomic inbreeding coefficient based on ROH (F_ROH_) detected in four subpopulations of Eld’s deer. Upper and lower edges of the box represent the upper and lower quartile, respectively; the midline of the box shows the median; dots represent outliers; and the shape of the violin plot indicates the density at any position. The violin plot was visualized using R; statistical significance is shown at *p* < 0.05. Sampling sites: SED-ZPOT, Siamese Eld’s deer of the Zoological Park Organization of Thailand); SED-DNP, Siamese Eld’s deer of the Department of National Parks, Wildlife and Plant Conservation; BED-ZPOT, Burmese Eld’s deer of the Zoological Park Organization of Thailand; BED-DNP, Burmese Eld’s deer of the Department of National Parks, Wildlife and Plant Conservation. *p < 0.05; **p < 0.01; ***p < 0.0001.

We used the detectRUNS package to identify runs of homozygosity (ROH) in the Eld’s deer population^27^. The distribution of 16,985 ROH in total was counted across five tract length classes, 0–6 Mb, 6–12 Mb, 12–24 Mb, 24–48 Mb and > 48 Mb (Fig. 5a and Supplementary Table 17). The majority of the ROH, detected in all 81 individuals, were in the smallest class size (87.29%). The bar plot (Fig. 5a) illustrates that ROH tract lengths in the SED population are found in all class sizes. The total number of detected ROH was significantly higher in the SED-DNP population (n = 6001) than in the SED-ZPOT population (n = 2869). However, the distribution of the longest ROH tract, ranging more than 48 Mb, was still observed in the SED population, accounting for 0.04% of the total detected ROH. These findings indicated that ROH derived from recent ancestors and close inbreeding were prevalent in the SED population. Furthermore, ROH tract length detected in the BED-DNP subpopulation was also observed in only short (0–6 Mb and 6–12 Mb) and intermediate (12–24 Mb) class sizes, whereas ROH allocated in BED-ZPOT were additionally found in the 24–48 Mb length category. Findings revealed that homozygosity observed in BED-DNP was likely to have been gained from more distant ancestors, compared to the BED-ZPOT subpopulation and thus, the chance of close breeding was less of a concern in this subpopulation.

We then used the ROH tract length to predict the time in generations of shared common ancestors, using the formula g = 100/(2rL), where g is the generation time, r is the recombination rate (cM/Mb) and L is the length of the ROH tract (Mb)^28^. The recombination rate in deer was set at 1.04 cM/Mb^29^. The longest ROH were detected in the SED-ZPOT and SED-DNP subpopulations (> 48 Mb), suggesting that these subpopulations were small in the recent past (< 1 generation ago) and might have suffered from close inbreeding effects. On the other hand, the BED-ZPOT subpopulation contained short and intermediate ROH tract lengths (24–48 Mb), indicating that the time of shared ancestors was longer than in the SED population (> 1.7 generation ago). However, ROH tracts less than 24 Mb were observed in the BED-DNP subpopulation, suggesting that the lineage shares a common ancestor as far back as more than two generations.

The genomic inbreeding coefficient based on ROH (F_ROH_) was depicted using a violin plot across the four Eld’s deer subpopulations (Fig. 5b and Supplementary Table 18). Comparing the two subspecies, the BED population had lower F_ROH_ values than the SED population. The average F_ROH_ coefficients of SED-ZPOT and SED-DNP were 0.39 ± 0.11 and 0.46 ± 0.07, respectively, whereas those of BED-ZPOT and BED-DNP were 0.17 ± 0.08 and 0.16 ± 0.03, respectively. The higher F_ROH_ coefficients of SED subpopulations compared to BED subpopulations indicate a history of close breeding in the population.

**Figure 6:**
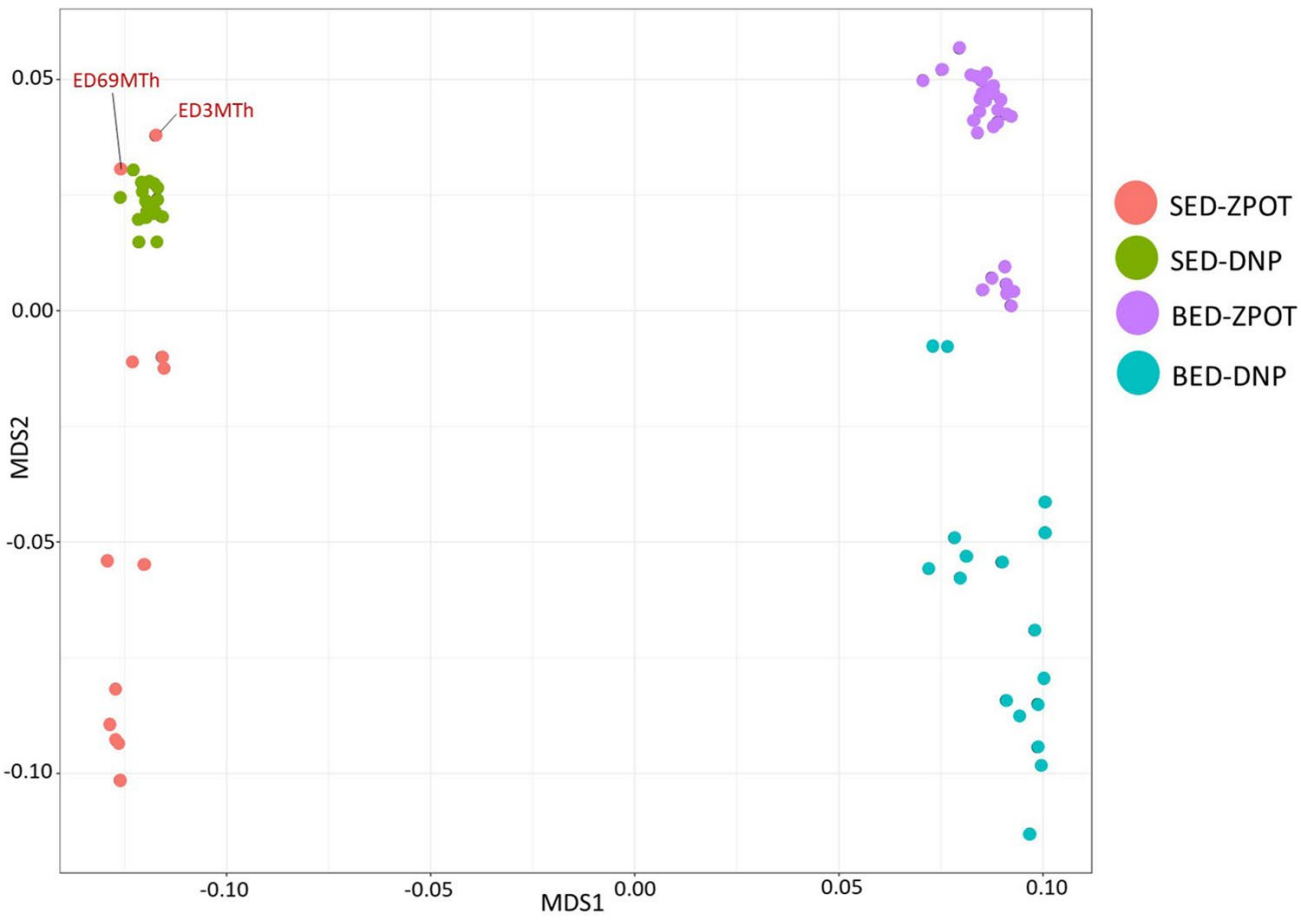
Multidimensional scaling (MDS) of 81 sequenced individuals of Siamese Eld’s deer (SED) and Burmese Eld’s deer (BED) populations. The MDS was depicted using R. Sampling sites: SED-ZPOT, Siamese Eld’s deer of the Zoological Park Organization of Thailand; SED-DNP, Siamese Eld’s deer of the Department of National Parks, Wildlife and Plant Conservation; BED-ZPOT, Burmese Eld’s deer of the Zoological Park Organization of Thailand; BED-DNP, Burmese Eld’s deer of the Department of National Parks, Wildlife and Plant Conservation. Sampling sites and specific organizations holding Eld’s deer: B, Banglamung Wildlife Breeding Center, DNP; C, Chulabhorn Wildlife Breeding Center, DNP; H, Huai Kha Khaeng Wildlife Breeding Center, DNP; K, Khao Kheow Open Zoo, ZPOT; N, Nakorn Ratchasima Zoo, ZPOT; U, Ubon Ratchathani Zoo, ZPOT.

**Figure 7:**
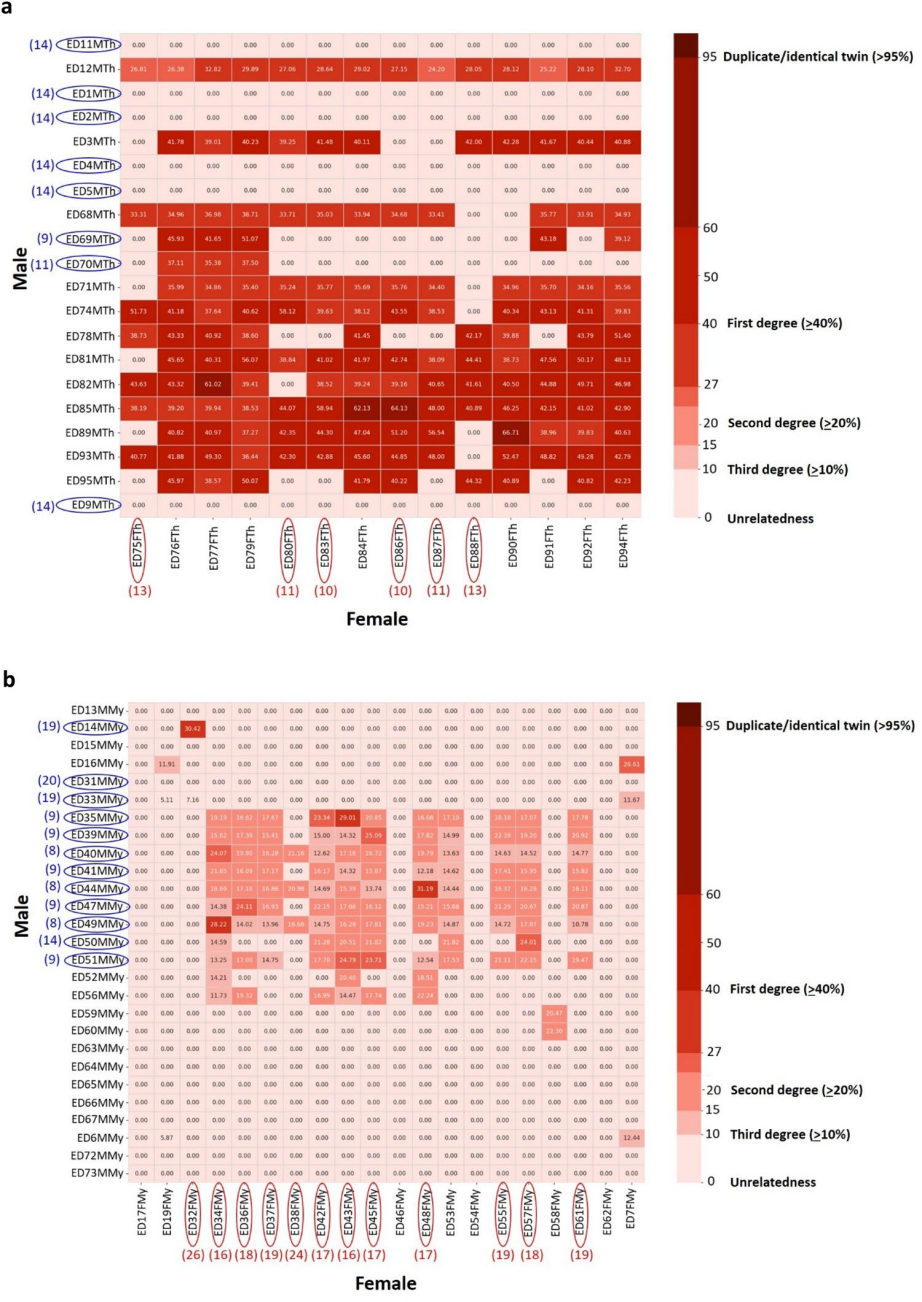
Heatmap of percentage of Pi-Hat coefficient calculated from samples of (**a**) Siamese Eld’s deer (SED) pairs and (**b**) Burmese Eld’s deer (BED) pairs by gender. Female individuals are indicated along the horizontal axis, whereas male individuals are depicted along the vertical axis. Individuals circled in blue denote male breeder candidates. Blue numbers in parentheses indicate numbers of females genetically unrelated to the selected males. Individuals circled in red denote female breeder candidates. Red numbers in parentheses indicate numbers of males genetically unrelated to the selected females.

The analysis of familial relatedness in 81 Eld’s deer individuals was conducted using the proportion of IBD alleles or Pi-Hat values, calculated from 33,708 filtered and LD-pruned SNPs. The results were visualized using a multidimensional scaling (MDS) plot, which shows the separation of SED and BED populations, consistent with the results from the phylogenetic tree and admixture analysis (Fig. 6). Notably, two individuals (ED3MTh and ED69MTh of ZPOT) appeared in the SED-DNP cluster, in agreement with the admixture analysis. The potential genetic relationships within SED and BED populations was determined using modified criteria from previous studies: (a) a relationship of greater than 95% indicates a duplicate or identical twin; (b) a relationship of 40% or more indicates a first-degree relationship (e.g. parent-offpsring, siblings); (c) a relationship of 20% or more indicates a second-degree relationship (e.g. grandparents, grandchildren, half-siblings); (d) a relationship of 10% or more indicates a third-degree relationship (e.g. great-grandparents, great-grandchildren, first cousins); and (e) a relationship of less than 10% is considered unrelated^30,31^. The heatmap of SED and BED populations by genders was plotted using Pi-Hat values of each pair. The numbers of Pi-Hat were scaled from dark red to light red, indicating high to low values of relatedness. The average Pi-Hat coefficient of the SED population was remarkably higher than that of the BED population, with values of 0.22 ± 0.21 and 0.02 ± 0.06, respectively, from 280 and 540 pairs. Despite the high risk of close inbreeding in the SED population, six male SED-ZPOT individuals (ED1MTh, ED2MTh, ED4MTh, ED5MTh, ED9MTh and ED11MTh) were found to have no genetic relationship with any of the 14 female SED-DNP individuals (Fig. 7a).

Two male SED-ZPOT individuals (ED70MTh and ED69MTh) had no genetic relationship with 11 and nine female SED-DNP individuals, respectively (Fig. 7a). Six female SED-DNP individuals (ED75FTh, ED88FTh, ED80FTh, ED87FTh, ED83FTh and ED86FTh) had no genetic relationship with more than 10 male SED individuals (Fig. 7a). In the BED population, several genetically unrelated male and female pairs were observed (Fig. 7b). However, concerning the genetic purity of the BED population, only 12 male and 12 female BED were purebred (Fig. 4). Of the purebred BED, two male BED-DNP individuals (ED31MMy and ED33MMy) were genetically unrelated to all 12 purebred female BED individuals (Fig. 7b). One male BED-ZPOT (ED14MMy) and one male BED-DNP (ED50MMy) had no genetic relationship with 11 and seven purebred female BED individuals, respectively (Fig. 7b). One female BED-ZPOT (ED32FMy) and one female BED-DNP (ED37FMy) had no genetic relatedness to 11 and nine purebred male BED individuals, respectively (Fig. 7b). If the admixture of BED with SED were considered low and omittable for breeding purposes, there would be nine more potential male breeders with no genetic relationship to all 20 females (Fig. 7b).

## Discussion

The declining Eld’s deer population has impacted the food chain balance and increased the risk of inbreeding. This study presents de novo genome and mitogenome assemblies, comparative mitogenomics, and comparative genomic analyses of expanded/contracted gene families and positively selected genes of the SED and BED lineages. Despite limited pedigree data, the genetic relationships and genetic purity of captive Eld’s deer populations in Thailand was analyzed. Based on the data obtained from RADseq, suitable breeding pairs were chosen in efforts to produce fawns with high genetic purity and reduce inbreeding effects. The SED hybrid genome assembly has a scaffold N50 of 24.62 Mb and a total size of 2.48 Gb, and the BED genome assembly has a scaffold N50 of 23.32 Mb and a genome size of 2.32 Gb. Compared to the sister taxon Père David’s deer, having a scaffold N50 of 2.85–3.03 Mb and a genome size of 2.52–2.58 Gb from Illumina short-read sequencing, the Eld’s deer assemblies are slightly smaller but have significantly higher scaffold N50 lengths, possibly owing to the sequencing approaches used^32,33^. Repetitive element analysis showed that long interspersed elements (LINEs) were predominant in Eld’s deer, similar to Père David’s deer. Our analysis also revealed that there were 24,913 and 28,831 predicted protein-coding genes in the genomes of SED and BED, respectively, which were higher compared to the previously reported numbers of 19,368 genes in red deer, 22,473 genes in hog deer and 20,651 genes in white-tailed deer^24,34,35^. Gene structures were predicted using homology-based and ab initio methods. Gene annotation can be further improved through the addition of transcriptomic (RNA-seq) evidence derived from different tissues.

We reported the complete mitogenomes of both SED and BED and found that the number of protein-coding genes, tRNA and rRNA of both subspecies were similar to that of Manipur Eld’s deer. The lengths of the SED and BED mitogenomes (16,357 and 16,358 bp, respectively) were the same or similar to that of Manipur Eld’s deer (16,357 bp)^36^. Our present study, based on the mitogenome data, revealed the genetic relationship of all subspecies of Eld’s deer, including SED, BED, Manipur Eld’s deer and Hainan Eld’s deer, for the first time. Our results showed that Eld’s deer is more closely related to Père David’s deer than to deer within the same genus, specifically the barasingha (*Rucervus duvaucelii*) (Fig. 1). This finding is consistent with previous research^37^, showing a phylogenetic tree of the Cervini tribe based on mitogenome data. That study also found that Manipur Eld’s deer and Père David’s deer were related together as a sister group. Our study showed a close relationship between BED and Manipur Eld’s deer, which is probably a result of their close geographical proximity. On the other hand, the disjunct distribution of SED and BED is likely to be attributable to the mountain ranges between Thailand and Myanmar, including the Daen Lao and Dawna ranges^10^. The close relationship between SED and Hainan Eld’s deer, respectively residing on the mainland and Hainan Island, was demonstrated. This is consistent with evidence from mitochondrial DNA D-loop sequences that suggested that Eld’s deer migrated from Indochina to Hainan via a land bridge during the Pleistocene (0.69 Mya ago)^16,37^. Our mitogenome findings are congruent with the previous phylogeny constructed using only mitochondrial DNA control region sequences^10^. Our analysis of complete mitogenomes of the two subspecies provided valuable data for understanding their evolutionary relationships.

In this study, the estimated divergence time based on 5470 single-copy orthologous nuclear genes between SED and BED versus Père David's deer of 2.34 Mya was close to previous estimates of 2.1–2.01 Mya for Manipur Eld's deer and Père David’s deer^5,37^. The divergence time between SED and BED in the present study of 1.26 Mya was estimated to be substantially earlier than previous estimates of 0.44 Mya between Manipur Eld's deer and Hainan Eld's deer^37^, suggesting that these lineages have been evolving independently since the Calabrian stage of the Pleistocene. Consistent with the results obtained from mitochondrial gene sequences, the estimated divergence time between the clade of SED and Hainan’s Eld’s deer and the clade of BED and sangai also displayed to be earlier than in the previous study. Comparative genomic analysis showed that gene families related to odor recognition (ORs) were expanded in the SED and BED subspecies, similar to other deer species, including forest musk deer, reindeer and sika deer^38–40^. It is possible that the expansion of these gene families allowed Eld's deer to better detect and respond to a wide range of odors in its environment, which would have helped it to survive and reproduce. Eld’s deer use the secretions released from the preorbital gland to mark their directory^41^. Additionally, we found the existence of expanded gene families related to endogenous retroviral integration in BED, indicating previous retroviral infections that have been incorporated into the host’s genome and subsequently passed down through successive generations^42,43^. The integration of endogenous retroviruses has contributed to the evolution of mammalian genomes^44^. It is possible that endogenous retroviruses produce proteins that are beneficial to the host genome, particularly in the modulation of innate immunity^45,46^.

Genome-wide SNPs generated by RADseq were used to explore genetic relationships within the population at an affordable cost. Based on the best *K*-value estimated from the genetic ancestry analyses, the results of the study showed that the whole population was divided into four subgroups in accordance with the subspecies and sampling locations (Fig. 4), and the clusters were correlated with the results of the phylogenetic tree and MDS plot (Fig. 3b and 6). Genetic ancestry analysis was previously used to infer population clustering and structure in grey wolf and forest musk deer populations^22,47^. Admixture analysis was also used to indicate the genetic purity within and between the SED and BED populations.

Two individuals (ED3MTh and ED69MTh) from the SED-ZPOT group were found to be admixed with the SED-DNP group, which was traced back in the family records (Fig. 4). We discovered that all parents of both individuals belonged to DNP. The mother of ED3MTh was transferred to ZPOT while she was pregnant and gave birth to ED3MTh there, while ED69MTh was moved from DNP to ZPOT when he was a fawn. Four other SED-ZPOT individuals (ED12MTh, ED68MTh, ED70MTh, and ED71MTh) were also found to contain a high proportion of SED-DNP genetic ancestry. Family records showed that ED12MTh was born to a DNP hind, whereas ED68MTh and ED70MTh were fawns of a DNP stag with unknown identity. The pedigree record of ED71MTh from DNP was lacking.

Focusing on the BED subpopulation, a very small amount of admixed SED genetic ancestry was observed in 23 individuals. According to the known pedigree record, five of these individuals (ED13MMy, ED64MMy, ED65MMy, ED72MMy and ED73MMy) were offspring of ED31MMy, a BED with pure genetic ancestry that came from Myanmar. This suggests that these five BEDs acquired their SED genetic ancestry from the maternal side (not included in the study). However, the proportion of admixture between SED and BED was found to be very low. This could be attributed to historical breeding between the two subspecies, which produce fertile hybrids^15,16^. Introgressive hybridization can potentially lead to the loss of distinct gene pools and the disruption of genetic purity in endangered species^48,49^. And mixing of these two highly divergent lineages should be avoided, which could possibly lead to outbreeding depression^50^. Our study demonstrated that genome-wide SNP analysis, based on RADseq, is a powerful and cost-effective tool that can help clarify hybridization and introgression in Eld’s deer, particularly in cases where hybrids cannot be reliably identified based on morphology alone.

Our evaluation of genomic diversity and inbreeding parameters showed a high likelihood of close breeding within the SED subspecies, as indicated by low heterozygosity, high genomic inbreeding coefficients and high F_ROH_ values (Fig. 5 and Supplementary Tables 17 and 18). This could be due to the fact that the captive SED lineage in Thailand was believed to have been originated from only three known founders that produced limited numbers of offspring^15,16^. Furthermore, SED have been found specifically in captivity, and breeding management programs have been typically confined to individual breeding centers or zoos with limited exchange with other lineages from different organizations or neighboring countries. By contrast, the BED population did not have as pressing an issue with low genetic diversity and inbreeding, probably because of a larger and diverse group of founders and easier reproduction, contributing to the successful reintroduction of BED^15^.

Our study found that the coefficient of inbreeding, F_ROH_, in the whole Eld’s deer population ranged from 0.11 to 0.56, with the majority of the higher values found in the SED subpopulation. These values were higher than those found in Père David's deer (F_ROH_ ranging from 0.11 to 0.16)^33^, indicating that Eld’s deer are more vulnerable to inbreeding. To guide repopulation efforts, we recommend selecting six male SED-ZPOT individuals ((ED1MTh, ED2MTh, ED4MTh, ED5MTh, ED9MTh and ED11MTh) to mate with all females from SED-DNP, and we also recommend selecting two males from SED-ZPOT (ED70MTh and ED69MTh) to mate with 11 and nine genetically unrelated females from SED-DNP, respectively. For the BED population, although a higher number of genetically unrelated males and females were observed, only some of those males without SED genetic ancestry were suggested as suitable breeders for mating with genetically unrelated females (Fig. 7b). To prevent high levels of inbreeding, genetic ancestry (to determine purity) and inbreeding analyses are important initial steps in selecting suitable breeders. In future repopulation efforts, matings should be planned as either natural or using assisted reproductive technology (ART), such as artificial insemination or in vitro fertilization, particularly when selected pairs are geographically distant or located in separated regions. Our ultimate goal is to achieve the systematic repopulation of Eld’s deer and other endangered species while avoiding inbreeding.

In summary, our study uncovered the genomic characteristics and genetic relationships of ex situ SED and BED populations. We recommend using genome-wide SNPs, generated through the RADseq method, to evaluate genetic ancestry and inbreeding levels in order to properly select breeders in animal populations that lack comprehensive pedigree records and/or are at high risk of inbreeding depression.

## Methods

### Ethical approval

All methods were carried out in accordance with guidelines and regulations and the study was carried out in compliance with the ARRIVE guidelines. All animals were managed following the ethical guidelines required under the Chulalongkorn University Animal Care and Use Committee (CU-ACUC), Thailand (approval number 2031071).

### De novo genome assemblies and annotations

Blood samples were collected from a 7-year-old male SED (ED3MTh) from Ubon Ratchathani Zoo, Ubon Ratchathani, and a 10-year-old male BED (ED14MMy) from Khao Kheow Open Zoo, Chonburi. Genomic DNA was extracted from whole blood. For SED, long-read and short-read sequencing was conducted using the Pacific Biosciences (PacBio) Sequel system and Illumina NovaSeq 6000 platform, which produced 147.8 and 97.0 Gb of raw data, respectively. Hybrid scaffolding was conducted by using the long-reads as a backbone and then combining these with short-read contigs. Then, PacBio contigs were reassembled against the hybrid scaffolding to fill gaps and improve contiguity. The assembled SED genome was then polished and corrected using the short-read data. This SED genome was used for a reference-guided genome assembly of the BED genome and for SNP identification by mapping RADseq data obtained from 84 other Eld’s deer samples. For the BED genome, only short-reads were sequenced using MGI sequencing technology with a 150-bp paired-end mode, which generated 121 Gb of raw data. The completeness of the SED and BED genome assemblies was evaluated using BUSCO v4.0.5^51^ by searching against single-copy orthologs with the mammalia_odb gene set (n = 9226). Furthermore, repetitive elements contained in the SED and BED assemblies were annotated using RepeatModeler v2.0.1 (http://www.repeatmasker.org/RepeatModeler.html). Protein-coding sequences and gene structures were also identified using Exonerate v2.2^52^ for homology-based prediction, and Augustus v3.3.3^53^ and SNAP v2006-07-28^54^ for ab initio prediction. Functional annotation of predicted genes and the putative non-coding RNAs and tRNAs is described in the Supplementary Methods.

### Mitochondrial genome assemblies, annotations and phylogeny inference

We also assembled the mitochondrial genomes of the SED and BED reference individuals. The complete mitogenomes were annotated, in terms of protein-coding, ribosomal RNA and transfer RNA genes. The best-fit model selection and a maximum likelihood phylogenetic tree was constructed using MEGA X. The mtREV24 + G + I model was selected as the best choice. The tree was built from an alignment of the concatenated 13 protein-coding genes, and it involved 1000 bootstrap replications, which also included the corresponding sequences from the published complete mitochondrial genomes of 28 other Cetartiodactyla species, retrieved from GenBank (Supplementary Table 6). The mitochondrial sequences of *H. amphibius* (hippopotamus; AP003425) and *O. orca* (killer whale; NC_023889) were used as outgroups (Supplementary Methods). The estimated divergence time was also computed on MCMCTree in the Phylogenetic Analysis by Maximum Likelihood package (PAML) v4.9j package^55^. The known fossil information of the node of the Cervinae and Munctiacinae subfamily was obtained as a calibration constraint^56^. The pairwise distances between each pair of mitochondrial genome sequences from SED, BED and the other 28 species were calculated using MEGA X^57^ and the p-distance method, based on the amino acid dataset of 13 protein-coding genes.

### Species-specific orthologous gene families

Protein-coding genes of SED, BED and three other mammalian species (*Homo sapiens*, *Elaphurus davidianus* and *Bos taurus*) were clustered into gene families using OrthoVenn2^58^. The species-specific orthologous gene families and enrichment pathways of SED and BED were revealed and depicted in a Venn diagram (Supplementary Methods).

### Phylogenetic tree, divergence time estimation and detection of expanded/contracted gene families

We aligned protein sequences from single-copy orthologous groups of SED, BED and eight other mammalian species using the MUSCLE program^59^ and constructed a maximum-likelihood phylogenetic tree with the RAxML-NG program. The best-fit model selection was conducted with ModelTest-NG, which identified the JTT + I + G4 + F model as the most suitable^60^ (Supplementary Table 7). The divergence times were estimated using MCMCTree from the PAML v4.9j package^55^ with two known fossil calibration priors and one secondary prior: (1) the split between Bovidae and Antilopinae^61^, (2) the split between goat and sheep^62,63^, and (3) the published divergence time between Père David’s deer and Eld’s deer^37^. Gene family expansion and contraction along the SED and BED lineages were evaluated across the phylogenetic branches (*p* < 0.01) with the gene birth-date (λ) parameters predicted by a maximum-likelihood calculation in CAFE v4.2.1^64^. GO enrichment pathway analysis was also performed using the Database for Annotation, Visualization and Integrated Discovery (DAVID)^65,66^. Pathways were considered to be significantly enriched at *p* < 0.05 (Supplementary Methods).

### Positively selected genes

To test for positive selection, coding sequences of single-copy orthologs from SED, BED and three selected species (*C. elaphus, C. h. yarkandensis and E. davidianus*) were analyzed using the PosiGene pipeline^67^. Candidate genes were considered as positively selected at a false discovery rate (FDR) < 0.05 and an ω value of > 1. Additionally, GO enrichment pathways involving biological pathways, molecular functions and cellular components were analyzed using DAVID^65,66^ (Supplementary Methods).

### RAD sequencing and SNP identification

We extracted genomic DNA from 83 whole blood samples and one muscle sample of 84 Eld’s deer individuals (35 SED and 49 BED) for RADseq (Supplementary Table 14). Samples were collected from six locations (Ubon Ratchathani Zoo, Nakhon Ratchasima Zoo, Khao Kheow Open Zoo, Chulabhorn Wildlife Breeding Center, Banglamung Wildlife Breeding Center and Huai Kha Khaeng Wildlife Breeding Center) that belonged to two main organizations of Thailand; ZPOT and DNP (Fig. 3a). The RAD libraries were constructed and then sequenced on the MGISEQ-2000RS platform. Clean reads were obtained and mapped against the previously assembled SED genome. SNPs were called using the Genome Analysis Toolkit Unified Genotyper (GATK) pipeline v4.2.3.0^68^ (Supplementary Methods).

### Phylogenetic and admixture analyses

To identify the phylogenetic relationships among individuals, the SNP dataset of 84 individuals was filtered to retain four-fold degenerate sites with the following criteria: (a) MAF between 0.1 and 0.9; (b) a depth of coverage between 10 × and 200 × ; and (c) missing data ≤ 10%. A set of 3067 filtered SNPs was then used to construct a maximum-likelihood phylogenetic tree using MEGA X^57^ with 1000 bootstrap replicates. The best-fit model selection was constructed using MEGA X^57^ and the T92 + G + I model was selected as the best choice. Moreover, a total SNPs of 1,726,048 was filtered using the following criteria: excluding SNPs with (a) > 10% missing genotype data; (b) MAF < 0.05; (c) significant level of Hardy–Weinberg equilibrium test > 0.01; and (d) excluding individuals with ≥ 15% missing genotype data to gain 273,187 SNP dataset for the analysis of population structure based on a Bayesian clustering method, implemented in ADMIXTURE v1.3.0^69^. The quality control and LD-pruning were applied using PLINK v1.9^26^. Three individuals (ED8FTh, ED10MTh and ED18MMy) were excluded from the analysis because of a high number of missing genotypes. A total of 33,708 qualified SNPs was used to estimate the optimal *K*-value with the lowest cross-validation (CV) error. The data were visualized using R v4.1.1 (Supplementary Methods).

### Analysis of inbreeding coefficient, identical by descent (IBD)-based relatedness estimation and ROH

We used a total of 33,708 qualified and LD-pruned SNPs to calculate the genetic diversity parameters using PLINK v1.9^26^. The pairwise IBD-based relatedness parameters, including PI_HAT, were also estimated. The familial relationship between individuals of the SED and BED populations was visualized in a multi-dimensional scaling plot and in heatmaps, constructed in R v4.1.1 and using an in-house Python script. We also detected ROH in the Eld’s deer populations. Inputs were prepared using PLINK v1.9^26^ and were run in R using the slidingRUNS.run function in the detectRUNS package^27^ with the following parameters: (a) windowSize = 15; (b) threshold = 0.05; (c) minSNP = 20; (d) maxOppWindow = 1; (e) maxMissWindow = 1; (f) maxGap = 10^6^; (g) minLengthBps = 250,000; (h) minDensity = 1/10^3^; (i) maxOppRun = NULL; and (j) maxMissRun = NULL. The distribution of ROH tracts across five different length classes was visualized on a bar chart. The F_ROH_ among Eld’s deer subpopulations was calculated and plotted using R v4.1.1 (Supplementary Methods).

### Supplementary Information

Supplementary Information.

## Data Availability

The whole genome sequencing data and genome assemblies generated in this study have been deposited in the NCBI database under BioProject PRJNA637163 and PRJNA776694. The whole genome sequencing of SED and BED have been submitted to NCBI with the accession numbers JACCHN000000000 and JAJHSM000000000. Mitochondrial genome assemblies for SED and BED are available in GenBank with the accession numbers OP205647 and OP235941. All other relevant data are available upon request.

## Code availability

Analyses were performed using open-source software tools, and the detailed parameters for each tool are outlined in the relevant methods section in the Supplementary information.
