# Supplementary material for: Comparative genomics and genome-wide SNPs of endangered Eld’s deer provide breeder selection for inbreeding avoidance
Source: Sci Rep. 2023 Nov 13;13:19806. doi: 10.1038/s41598-023-47014-x (PMC10643696; doi:10.1038/s41598-023-47014-x)
Supplement: Supplementary file 1 — Supplementary Information. [file 41598_2023_47014_MOESM1_ESM.docx]

**Supplementary information**

### Comparative genomics and genome-wide SNPs of Eld’s deer provide

### the breeder selection for inbreeding avoidance

### Vichayanee Pumpitakkul^+^, Wanna Chetruengchai^+^, Chalurmpon Srichomthong, Chureerat Phokaew, Wirulda Pootakham, Chutima Sonthirod, Wanapinun Nawae, Sissades Tongsima, Pongsakorn Wangkumhang, Alisa Wilantho, Yongchai Utara, Ampika Thongpakdee, Saowaphang Sanannu, Umaporn Maikaew, Suphattharaphonnaphan Khuntawee, Wirongrong Changpetch, Phairot Phromwat, Kacharin Raschasin, Phunyapat Sarnkhaeveerakul, Pannawat Supapannachart, Wannapol Buthasane, Budhan S. Pukazhenthi, Klaus-Peter Koepfli, Prapat Suriyaphol, Sithichoke Tangphatsornruang^*^, Gunnaporn Suriyaphol^*^, Vorasuk Shotelersuk

^+^Contributed equally to this work

^*^Correspondence: [Gunnaporn.V@chula.ac.th](mailto:Gunnaporn.V@chula.ac.th); sithichoke.tan@biotec.or.th

**Supplementary methods**

**Sample collection.** We obtained 83 whole blood samples and one muscle sample from 84 Eld’s deer individuals (35 SED and 49 BED) from two main organizations in Thailand, ZPOT (Ubon Ratchathani Zoo in Ubon Ratchathani province, Nakhon Ratchasima Zoo in Nakhon Ratchasima province, and Khao Kheow Open Zoo in Chonburi province) and DNP (Chulabhorn Wildlife Breeding Center in Srisaket province, Banglamung Wildlife Breeding Center in Chonburi province, and Huai Kha Khaeng Wildlife Breeding Center in Uthai Thani province). Additionally, we used two samples from a 7-year-old male SED (ED3MTh) and a 10-year-old male BED (ED14MMy) for whole-genome sequencing (WGS) and *de novo* genome assembly (Supplementary Table 1).

**DNA isolation, library preparation and whole genome sequencing.** We extracted genomic DNA from 600-µL whole blood samples of SED (ED3MTh) and BED (ED14MMy) using the Qiagen DNeasy Blood and Tissue kit (Qiagen) and the Quick-DNA Miniprep Plus Kit (Zymo Research) for long-read and short-read WGS, respectively. We evaluated the integrity and quality of the gDNA by pulse-field gel electrophoresis and quantified DNA concentration using a NanoDrop One Microvolume UV-Vis Spectrophotometer (Thermo Fisher Scientific) and a Qubit fluorometer (Life Technologies) before sequencing. For the SED, we prepared a library of Illumina short-read sequencing by fragmenting and ligating adapters at both ends of DNA strands with a 550 bp insert size, using the TruSeq Nano DNA Kit (Illumina). Paired-end sequencing was conducted on the Illumina NovaSeq 6000 platform (Illumina) with the 2×150 bp mode. To produce long-fragment reads for single-molecule real-time (SMRT) sequencing, we constructed a large-insert library (>30 kb) using an SMRTbell Express Template Preparation Kit (Pacific Biosciences). A total of 15 SMRT cells were run on the Pacific Biosciences (PacBio) Sequel System with 600-min movie times. We also sequenced the genome of a BED. We used approximately 400 ng of gDNA with a peak size of 300–500 bp to construct a library using an MGIEasy FS DNA Library Prep Set (BGI Group). The library was sequenced on the MGISEQ-2000RS, using the MGISEQ-2000 RS Sequencing Flow Cell V3.0 (BGI Group) to generate 150 bp paired-end reads.

**Genome size estimation using k-mer analysis.** We used k-mer analysis to estimate the genome size of the SED and BED assembled genomes using GenomeScope v1.0^1^. At a k-mer size of 27, the estimated genome sizes of SED and BED were 2.49 Gb and 2.30 Gb, respectively.

**De novo genome assemblies.** To perform the hybrid assembly approach of the SED genome, we used data obtained from both Illumina short-read and PacBio long-read sequencing. Firstly, we determined the quality of the raw Illumina reads by FastQC, and the reads were then assembled using Minia v3.2.0 (http://minia.genouest.org/)^2^. The PacBio long-reads were subsequently trimmed using DEXTRACTOR v1.0^3^. Reads with a length ≥10 kb and Q > 0.8 were selected for assembly, and the clean reads were assembled into contigs using Canu v1.8^4^.

For scaffolding, we performed the hybrid assembly of Illumina contigs and PacBio contigs, as a backbone, using SSPACE-LongRead software v1.1^5^. Furthermore, to obtain a more contiguous final assembled genome, PacBio contigs were reassembled against the hybrid scaffolds using Quickmerge Meta-assembler v0.3^6^. The final assembled genome of SED was polished and error corrected using Pilon v1.16^7^.

Regarding the BED genome, we used Trim Galore v0.6.5^8^ to trim low-quality bases and adapters from the MGI raw reads. We constructed BED contigs using ABySS v.2.3.1^9^ with a k-mer size of 96. In the present study, we used the SED genome as a guided reference for BED scaffolding and assembly correction using RagTag v1.1.0^10^. We evaluated the completeness of both Eld’s deer genome assemblies using Benchmarking Universal Single-Copy Orthologs (BUSCO) v4.0.5 by searching for 9,226 single-copy orthologs in the mammalian gene set (mammalia_odb10.2019-11-20).

**Annotation of repeat elements.** We identified repetitive elements and families in the unannotated assembled genomes of SED and BED using RepeatModeler v2.0.1. These were then aligned to the GenBank non-redundant protein sequence database using BLASTX with an e-value cutoff of 1×10^–6^. This step was performed to ensure that the large families of protein-coding genes were not included in transposable elements. We used RepeatMasker v4.0.6^11^ to combine and mask repetitive sequences from both the *de novo* repeat library generated by RepeatModeler and homology-based searching against RepeatMasker consensus library 20150807 (www.girinst.org).

**Gene prediction and functional annotation.** We annotated protein-coding sequences and gene structures using two approaches: homology-based and *ab initio* predictions. For the homology-based search, we aligned protein sequences from Ensembl release 100, including *Bos taurus* (cattle), *Homo sapiens* (human), *Sus scrofa* (wild boar), *Ovis aries* (sheep), *Equus caballus* (horse) and *Camelus dromedarius* (Arabian camel), to the assembled genomes using Exonerate v2.2 in the MAKER annotation pipeline^12^. We used the gene model of the closely related species *Elaphurus davidianus* (Père David's deer) as an alternative. For the *ab initio* approach, gene prediction was obtained using Augustus v3.3.3 with model parameters trained on *H. sapiens* genes (RRID: SCR_008417). Additionally, we employed SNAP v2006-07-28 for *ab initio* prediction. The parameters settings were computationally optimized by a training set in the first run of the MAKER annotation pipeline. Genes were filtered based on the following criteria: (a) minimum coding sequence length of 150 bp, (b) maximum intron length of 10 kb, and (c) genes containing fewer than three exons, which could not be aligned well in either the UniProtKB/Swiss-Prot v5 or GenBank non-redundant database v5 (80% query coverage).

### Functional annotation of SED and BED predicted genes was assigned using OmicsBox v2.0 (https://www.biobam.com/downloadomicsbox/). We aligned protein sequences based on the best match alignment to annotated proteins retrieved from protein databases, including the UniProKB/Swiss-Prot and GenBank non-redundant protein sequence database V5, by local BLASTP with an e-value cutoff of 1×10^–613^. Gene Ontology (GO) term mapping, Kyoto Encyclopedia of Genes and Genomes (KEGG) pathway and enzyme commission (EC) number were assigned to and retrieved from the SED and BED query sequences. Specifically, we identified the functional annotation of protein-coding genes in the KEGG pathway using the KEGG Automatic Annotation Server (KAAS) with bidirectional best hit (BBH) BLAST methods (https://www.genome.jp/kegg/kaas/). A bar chart of KEGG classification with the numbers of involved genes was plotted using GraphPad Prism (GraphPad Software, San Diego, CA, USA).

**Annotation of non-coding RNA.** For annotation of non-coding RNAs, we identified putative short non-coding RNAs using StructRNAfinder v1.0^14^. Various tools automatically utilized by the pipeline, including Infernal v1.1.4^15^, RNAfold v2.4.18^16^ and Rfam 12.0 database^17^**,** were used to predict non-coding RNAs and determine their secondary structures. tRNAs were annotated using tRNAscan-SE v1.23 with default parameters^18^. Additionally, the gene content in both subspecies was evaluated using BUSCO v4.0.5.

### Mitochondrial genome assemblies and annotation. We identified the mitochondrial genome sequences of both SED and BED and we assembled the complete mitochondrial genomes using NOVOPlasty v3.8.2^19^. Next, we annotated mitochondrial genomes using the MITOS web server^20^, with protein-coding, rRNA and tRNA genes determined using the NCBI Basic Local Alignment Search Tool (BLAST)^21^. We visualized the circular structure of mitogenomes using OrganellarGenomeDRAW (OGDRAW) v1.3.1^22^. Thirteen conserved PSGs (CYTB, ND1, ND2, ND3, ND4, ND4L, ND5, ND6, COX1, COX2, COX3, ATP6 and ATP8) of SED, BED and 28 other cetartiodactyls were concatenated and subjected to multiple alignments using MUSCLE. Prior to the construction of the maximum likelihood phylogeny, the best-fit model was selected using MEGA X and the mtREV24+G+I model was selected. Subsequently, the phylogenetic tree was generated using MEGA X with 1,000 bootstrap replications, and we employed *Hippopotamus amphibius* (hippopotamus, NC_000889) and *Orcinus orca* (killer whale, NC_023889) JTT+I+G4+F as an outgroup. The resulting tree was visualized using Interactive Tree Of Life (iTOL) v6^23^. The divergence time estimation was performed using MCMCTree in the PAML 4.9j package based on the alignment of protein sequences. CODEML (in the PAML 4.9j package) was employed to calculate the appropriate Hessian matrix using an empirical rate matrix and gamma rates (WAG+Gamma). All ambiguous characters and gaps were defined as missing data in the calculation of the likelihood. The MCMC chains were run for 2 million cycles, and sampling was performed every 10 iterations. The program ignored the first 2,000 iterations due to the burn-in setting. The known fossil calibration constraint of the node of the Cervinae and Munctiacinae subfamily estimated at 7−9 Mya was acquired as a calibration point.

### Comparative genomic analysis. We used OrthoVenn2 to group the protein-coding genes of SED, BED, and three other mammals including *Homo sapiens* (human), *Elaphurus davidianus* (Pere David’s deer), and *Cervus elaphus* (red deer) into orthologous clusters. We visualized the species-specific gene families among the five genomes using a Venn diagram. We annotated the gene clusters specific to SED and BED according to the Swiss Protein Database in terms of biological process, molecular function, and cellular component. Additionally, we performed pathway enrichment analysis. Next, we identified protein sequences of single-copy orthologous groups of SED, BED, *H. sapiens* (human), *Moschus moschiferus* (Siberian musk deer) *B. taurus* (cattle), *O. aries* (sheep), *Capra hircus* (goat), *E. davidianus* (Pere David’s deer), *C. elaphus* (red deer) and *Cervus hanglu yarkandensis* (Yarkand deer) using OrthoFinder^24^. We then used these sequences in the analyses of phylogeny and expanded and contracted gene families. Firstly, sequences in each orthologous group were aligned using MUSCLE and alignment gaps were removed using the trimAl tool^25^ with the automated1 heuristic method. The concatenation of alignment blocks was retrieved with the catsequences program (http://github.com/ChrisCreevey/catsquences), and the substitution model for each block was predicted by the ModelTest-NG program^26^. These outputs were then used to construct a phylogenetic tree with the maximum-likelihood method in RAxML-NG, employing the JTT+I+G4+F best-fit model. We estimated the time of divergence among lineages/species via MCMCTree in the PAML 4.9j package. The alignment of amino acid sequences was used and the approximate likelihood method was computed. CODEML (in the PAML 4.9j package) was employed to calculate the appropriate Hessian matrix using an empirical rate matrix and gamma rates (WAG+Gamma). All ambiguous characters and gaps were defined as missing data in the calculation of the likelihood. The MCMC chains were run for 2 million cycles, and sampling was performed every 10 iterations. The program ignored the first 2,000 iterations due to the burn-in setting. In the analysis, we utilized two known fossil calibration priors and one secondary prior, incorporating the following estimates: (a) the split between Bovinae (cow) and Antilopinae (sheep), estimated at 18.3–28.5 MYA; (b) the split between *C. hircus* (goat) and *O. aries* (sheep), estimated at 5–7 MYA, and (c) the published divergence time between *E. davidianus* (Père David’s deer) and *Rucervus eldii* (Eld’s deer), estimated at 1.5–2.5 MYA. We evaluated expanded and contracted gene families across the phylogenetic tree using CAFE v4.2.1, with the gene birth-date parameters estimated by the maximum-likelihood method. The rapidly expanded and contracted gene families were revealed at *p* < 0.01.

### To search for the genes under positive selection on the genome-wide scale, we used the PosiGene pipeline, with protein-coding sequences as input. *C. h. yarkandensis* (Yarkand deer) was set as the anchor species for PosiGene. Sequences of each species were assigned to ortholog groups using the BBH BLAST search^27,28^. Multiple sequence alignments were initially computed using CLUSTALW^29,30^ and phylogenetic trees were reconstructed using the PHYLIP package^31^. All isoform assignments were aligned using PRANK^32^ on a codon level. We used the branch-site test of positive selection in the PAML 4.9j package to identify genes under positive selection at ω > 1 and FDR < 0.05. We evaluated GO pathway enrichment of expanded/contracted gene families and PSGs using DAVID in terms of biological pathway and molecular function. The significantly enriched pathways were determined at *p* < 0.05.

### RAD sequencing and SNP identification. To determine genetic variation and inbreeding status, we performed RADseq on 84 samples. We extracted genomic DNA using the Quick-DNA Miniprep Plus Kit (Zymo Research), following the manufacturer’s protocol, and quantified DNA concentration using a NanoDrop One Microvolume UV-Vis Spectrophotometer (Thermo Fisher Scientific) and a Qubit fluorometer (Life Technologies). We evaluated sample integrity by pulse-field gel electrophoresis. The DNA samples were diluted to a final concentration of 0.05 µg/µL for library construction.

### We constructed the RAD library using an MGIEasy RAD Library Prep Kit (MGI Tech, Shenzhen, China) following the manufacturer’s protocol. We used two restriction enzymes, *Taq*I and *Mse*I, to digest 1 µg of genomic DNA. Briefly, after the enzymatic fragmentation, RAD adapters and barcodes were added to ligate the digested fragments. Next, the products from all samples were pooled, and the quality of library construction was evaluated by assessing fragment size distribution using the Fragment Analyzer System (Agilent Technologies). The final PCR-amplified library fragments in the 400−450 bp size range were converted into a single-strand circularization DNA library and submitted for sequencing on the MGISEQ-2000RS platform (MGI Tech) to generate 150-bp paired-end reads. Raw reads were demultiplexed according to their barcodes. The sequences of adapters, barcodes and low-quality reads were discarded. Clean reads from all samples were mapped against the previously constructed SED genome assembly using Bowtie2 v2.4.2^33^. The genome-wide SNPs were called using SAMtools v1.3.1^34^ and the Genome Analysis Toolkit Unified Genotyper (GATK) v4.2.3.0 to obtain a multi-sample variant call format (VCF) file for further downstream analysis.

### Admixture analysis. The SNPs obtained from the 84 individuals were qualified using the following criteria in PLINK v1.9: (a) removing SNPs with > 10% missing genotype data (--geno 0.1); (b) minor allele frequency > 0.05 (--maf 0.05); (c) significance level of HWE test > 0.01 (--hwe 0.01) and (d) removing individuals with > 15% missing genotype data (--mind 0.15). The number of SNPs obtained after filtering was 273,187 for 81 individuals. LD pruning was conducted to remove redundancy, by calculating within a sliding window size of 50 SNPs, shifting five SNPs each time, and an r^2^ threshold of 0.5 (--indep-pairwise option: 50 5 0.2) using PLINK v1.9. A total of 33,708 filtered SNPs were used in the admixture analysis. We tested the cross-validation (CV) error, ranging from *K* = 1 to *K* = 20 (Supplementary Figs 4 and 5). We determined the best *K*-value based on the lowest CV error. We created a bar plot of each population cluster using R v4.1.1.

**Inbreeding coefficient, IBD-based relatedness estimation and runs of homozygosity (ROH) analysis.** For genetic diversity analysis, a dataset of 33,708 filtered and LD-pruned SNPs from 81 individuals was computed and analyzed using the --het function in PLINK v1.9. Genetic diversity parameters, including observed homozygosity, expected homozygosity, number of non-missing genotypes and genomic inbreeding coefficient, were identified for each subpopulation and individual. The observed heterozygosity rate was calculated using the formula:

Observed heterozygosity rate = (N(NM) − O(Hom))/N(NM)

where N(NM) is the number of non-missing genotypes and O(Hom) is observed homozygosity^35^. We used the values of heterozygosity rate and genomic inbreeding coefficient to test the statistical difference between subpopulations. We used the Shapiro–Wilk test for normality testing, and performed one-way ANOVA, with statistical significance considered at *p* < 0.05.

Additionally, we determined the familial relationships of SED and BED populations separately using the --genome function in PLINK v1.9. The Pi-Hat coefficient and the probability of sharing zero (Z0), one (Z1), and two (Z2) IBD alleles were estimated. We used the Pi-Hat coefficients to plot the MDS via R v4.1.1, and the percentage of shared Pi-Hat values was applied to construct the heatmap using an in-house Python script. The Pi-Hat coefficient was calculated using the formula:

$\hat{\pi}=P\left( IBD=2 \right)+0.5P(IBD=1)$

where P(IBD = 1) is the probability of sharing one allele (Z1) and P(IBD = 2) is the probability of sharing two alleles (Z2). The relatedness between pairs of Eld's deer, separated by subspecies (SED/BED) and genders (male SED/female SED, male BED/female BED), was visualized using a heatmap generated by an in-house Python script. Dark red blocks indicated high allele sharing between pairs, and the heatmap was used to predict the degree of relatedness (Fig. 7). In addition, we evaluated the ROH for each individual and subpopulation, including the number of ROH tract lengths per class-size (Mb) and the F_ROH_. To perform the ROH analysis, input files (.ped and .map) were prepared using the --recode option in PLINK v1.9 and then the detectRUNS 0.9.6 package in R v4.1.1 was run using the slidingRUNS.run function with the following parameters: (a) windowSize = 15; (b) threshold = 0.05; (c) minSNP = 20; (d) maxOppWindow = 1; (e) maxMissWindow = 1; (f) maxGap = 10^6; (g) minLengthBps = 250000; (h) minDensity = 1/10^3; (i) maxOppRun = NULL; and (j) maxMissRun = NULL. The analysis was conducted using five different length classes (0–6 Mb, 6–12 Mb, 12–24 Mb, 24–48 Mb and >48 Mb). The F_ROH_ coefficient was calculated using the formula:

$$FROH= \frac{\sum LROH}{Lgenome}$$

### where ∑L_ROH_ is the sum of the length of all detected ROH and L_genome_ is the total length of the applied genome^36^. This was performed using the Froh_inbreeding function in the detectRUNS package. The F_ROH_ among the four Eld’s deer subpopulations was visualized using a violin plot, and the statistical difference between subpopulations was determined using the Kruskal–Wallis test, with a significant difference considered at *p* < 0.05.

### Supplementary Figures

**Supplementary Figure 1. KEGG pathway classifications of the assembled sequences from Eld’s deer. a** KEGG pathway classifications of the assembled sequences from Siamese Eld’s deer. **b** KEGG pathway classifications of the assembled sequences from Burmese Eld’s deer. The bar chart shows the distribution of the number of proteins in different KEGG functional categories annotated using the KEGG Automatic Annotation Server (KAAS). The diagram was drawn using GraphPad Prism.

**a**

**b**

**Supplementary Figure 2. Complete mitogenomes of *Rucervus eldii* generated by OrganellarGenomeDRAW (OGDRAW). a** Complete mitogenome of *Rucervus eldii siamensis*. **b** Complete mitogenome of *Rucervus eldii thamin*. The complex I (NADH dehydrogenase), complex IV (cytochrome c oxidase), ATP synthase, ribosomal RNAs, transfer RNAs and cytochrome *b* are annotated. The genes outside the circle are transcribed clockwise, whereas the genes inside are transcribed counterclockwise. The inner ring shadow indicates the GC content of the genome.

**a**

**
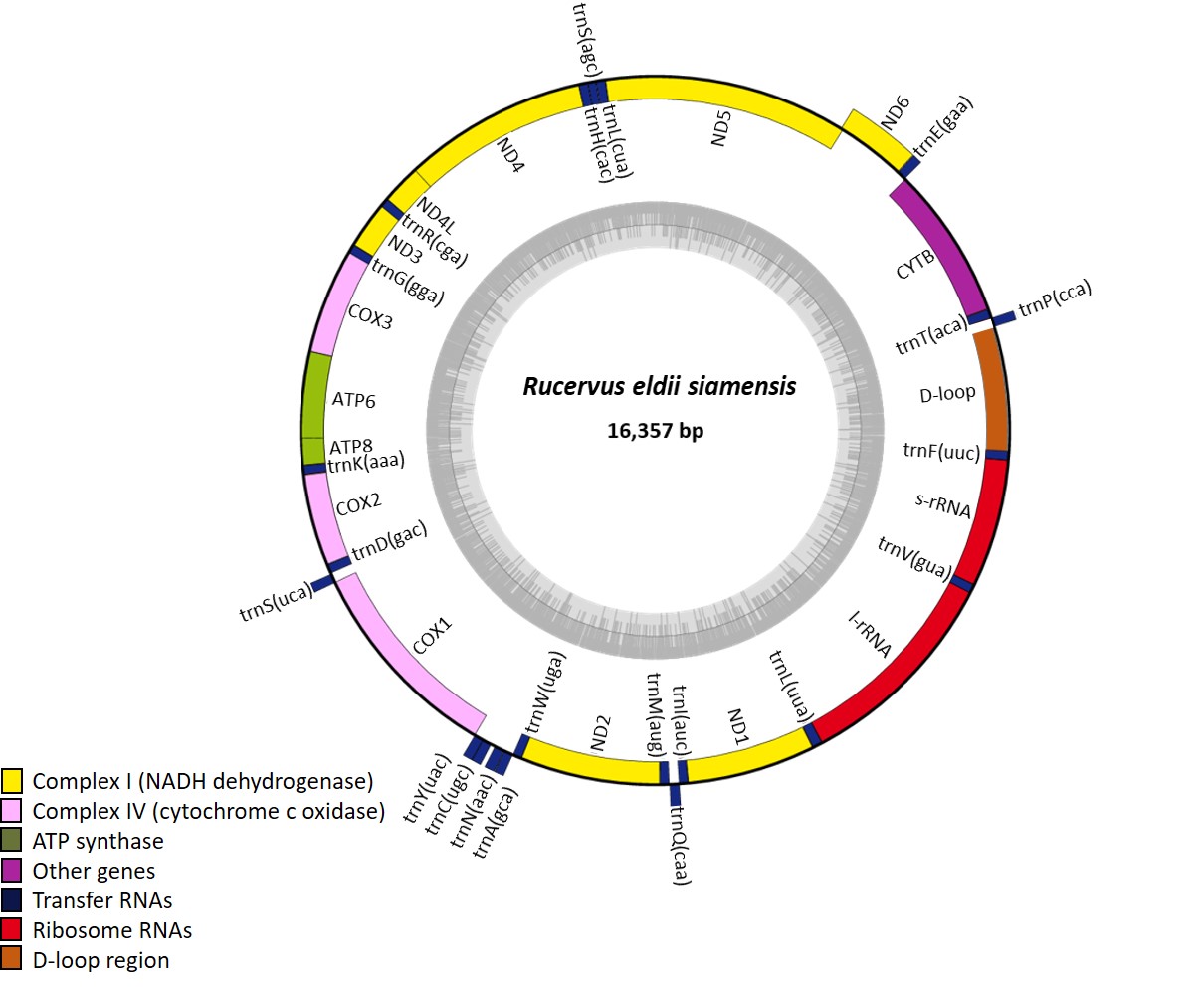
**

**b**


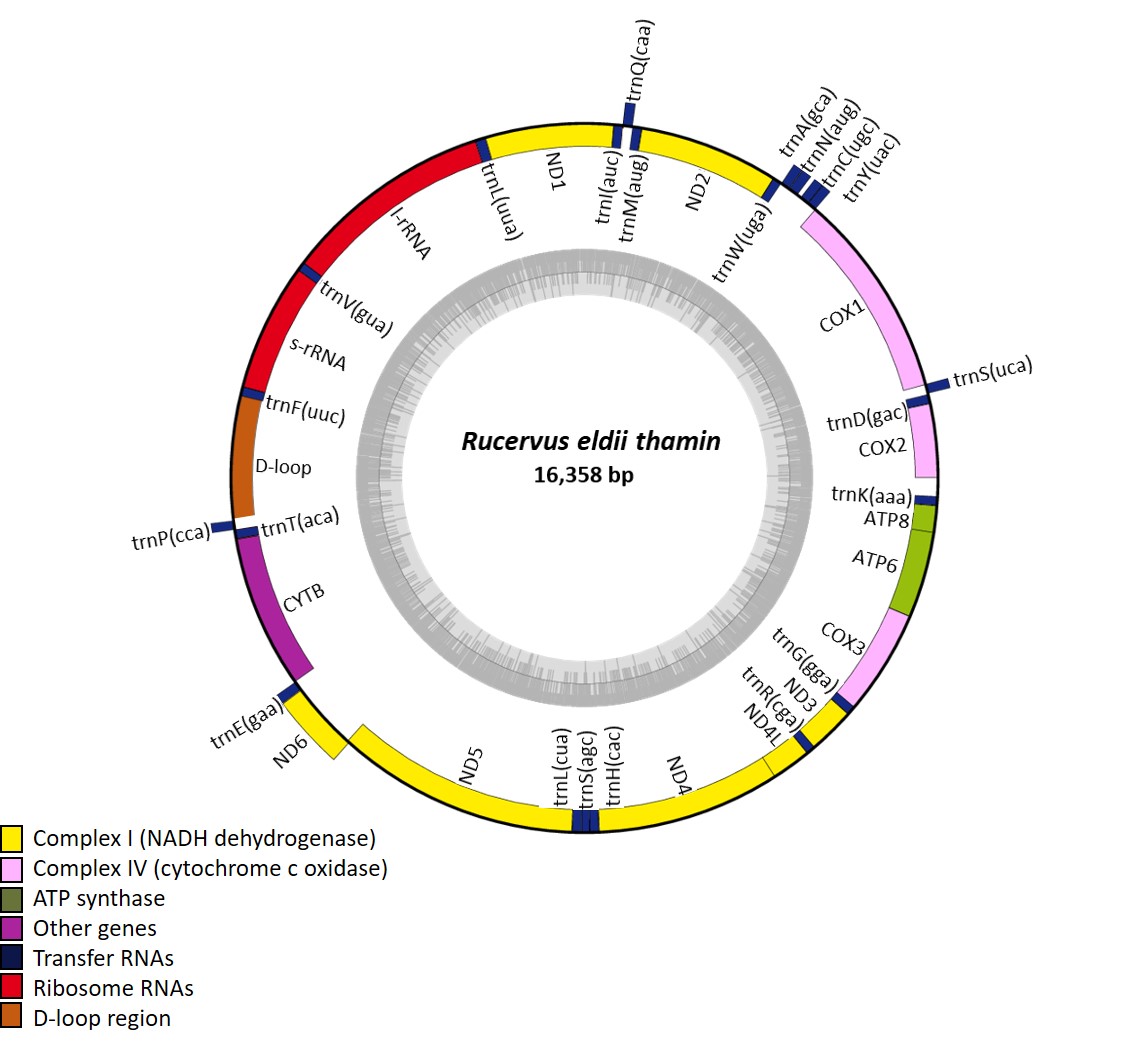


**Supplementary Figure 3. Phylogenetic tree with divergence time estimation.** A maximum-likelihood phylogenetic tree based on 5,470 single-copy orthologous genes was constructed using the RAxML-NG program. Divergence times were computed with MCMCTree in the PAML v4.9j package. The divergence time estimates are shown at each node with 95% confidence intervals (CI) in parentheses.


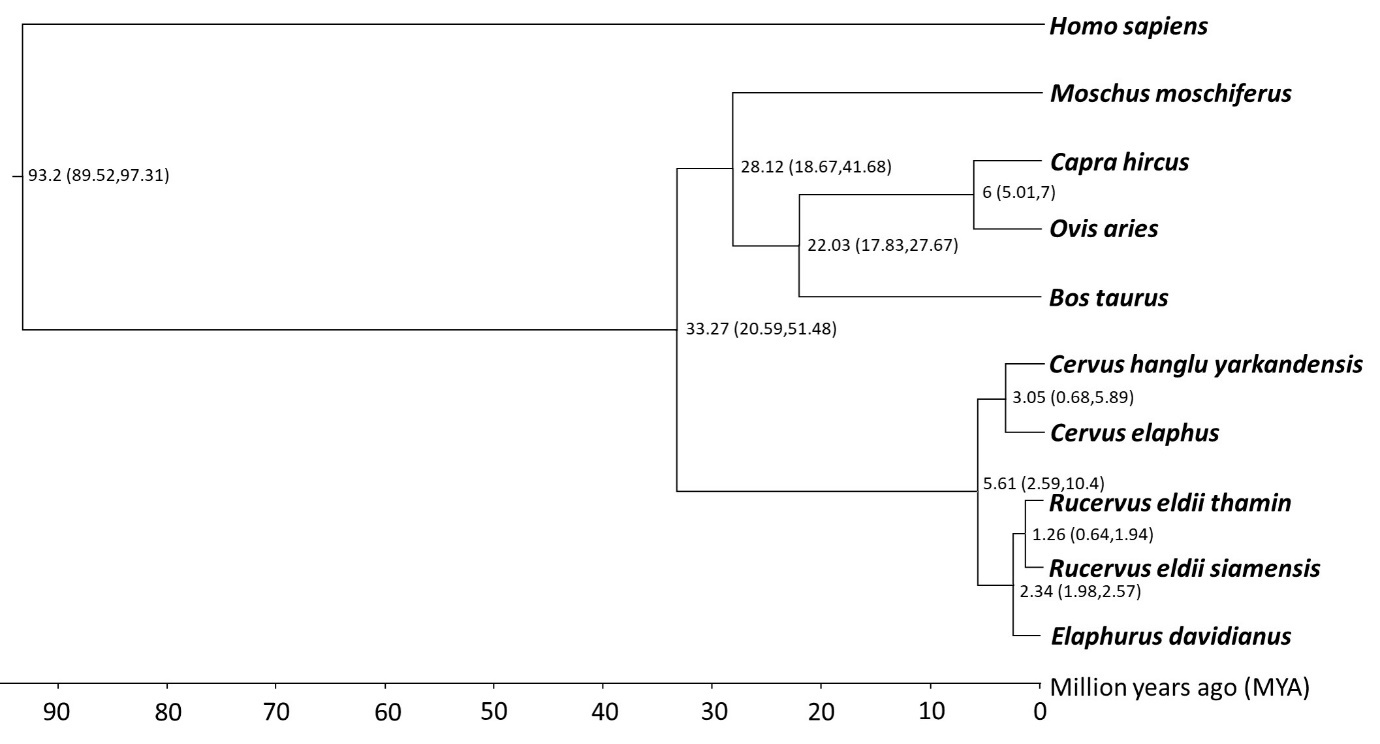


**Supplementary Figure 4.** Cross-validation (CV) error plot for *K*-values from 1 to 20 with 81 individuals and 33,708 single nucleotide polymorphism loci.

###
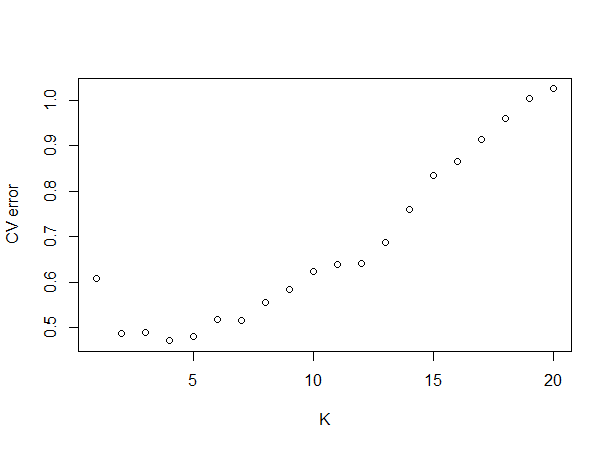


**Supplementary Figure 5. ADMIXTURE analysis of SED and BED populations with *K* = 2 to *K* = 5.** The color of each vertical bar represents the ancestral component and the proportion determines the estimated genetic ancestry per individual. The bar graph is plotted using R. Sampling sites: SED-ZPOT, Siamese Eld’s deer of the Zoological Park Organization of Thailand; SED-DNP, Siamese Eld’s deer of the Department of National Parks, Wildlife and Plant Conservation; BED-ZPOT, Burmese Eld’s deer of the Zoological Park Organization of Thailand; BED-DNP, Burmese Eld’s deer of the Department of National Parks, Wildlife and Plant Conservation.

###
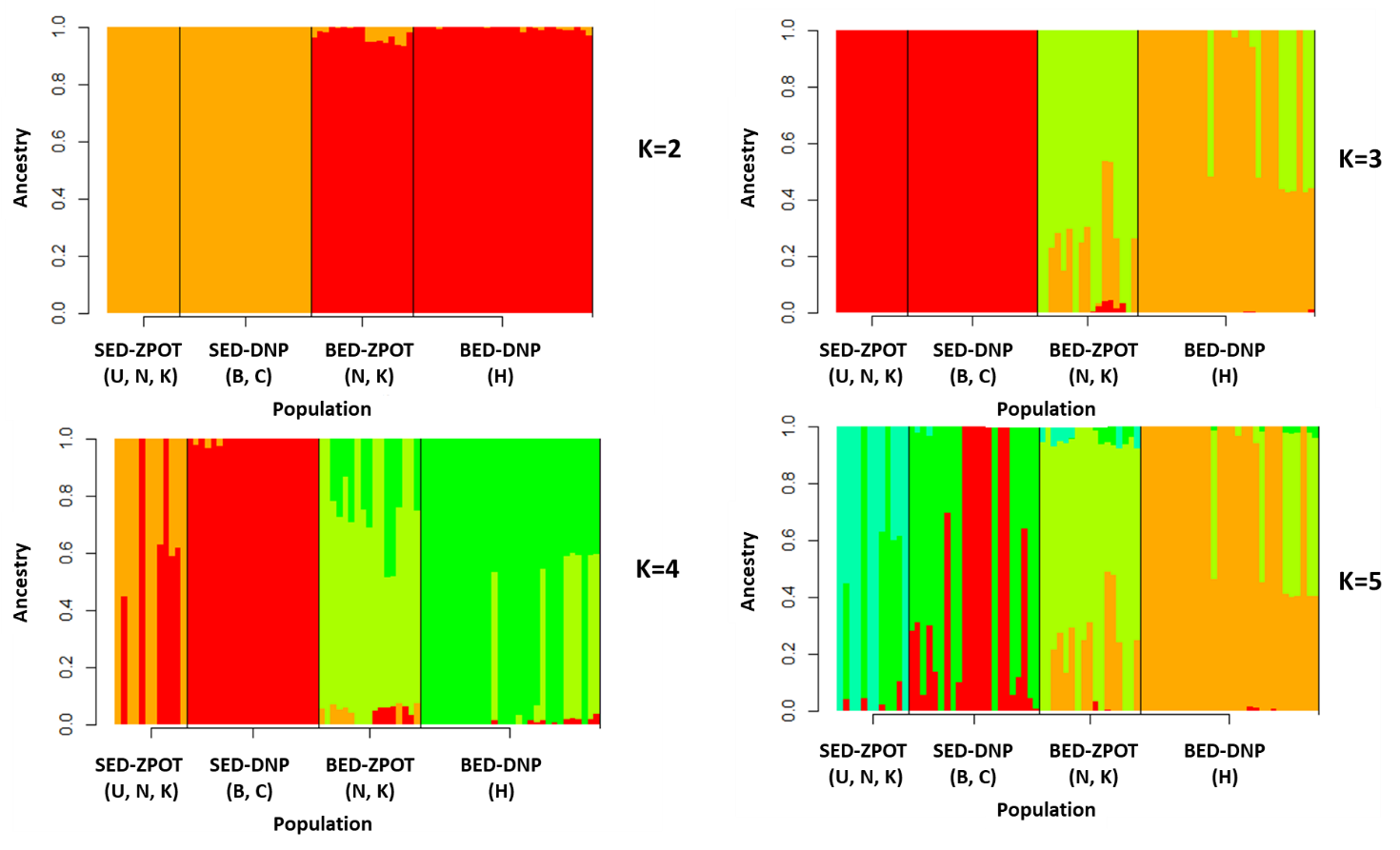


### Supplementary Tables

**Supplementary Table 1.** **BUSCO completeness assessments for genomic** **data quality control of** **the Siamese (SED) and Burmese (BED) Eld’s deer genome assemblies.**

| **Assembled genome** | **SED** | **BED** |
| --- | --- | --- |
| Complete and Single-copy BUSCO (%) | 90.3 | 89.4 |
| Complete and Duplicated BUSCO (%) | 2.1 | 0.6 |
| Fragmented BUSCO (%) | 1.6 | 2.6 |
| Missing BUSCO (%) | 6.0 | 7.4 |
| Total number of gene (mammalia_odb10.2019-11-20) | 9,226 | 9,226 |

**Supplementary Table 2.** **Repeat element content of the Siamese (SED) and Burmese (BED) Eld’s deer genomes.**

| Types of repeats | Number of loci | Length occupied (bp) | Percentage of the assembly |
| --- | --- | --- | --- |
| *SED* |  |  |  |
| Retrotransposons: |  |  |  |
| LINE | 2,506,591 | 712,513,770 | 28.68 |
| SINE | 307,116 | 37,312,839 | 1.50 |
| LTR | 242,353 | 73,198,038 | 2.95 |
| DNA elements | 200,378 | 31,682,066 | 1.28 |
| Simple sequence repeats | 506,123 | 20,930,498 | 0.84 |
| Low complexity | 87,371 | 4,298,438 | 0.17 |
| Unclassified | 44,333 | 13,398,138 | 0.54 |
| *BED* |  |  |  |
| Retrotransposons: |  |  |  |
| LINE | 2,433,625 | 599,055,403 | 25.77 |
| SINE | 301,545 | 36,494,414 | 1.57 |
| LTR | 236,498 | 68,052,629 | 2.93 |
| DNA elements | 192,037 | 30,471,891 | 1.31 |
| Simple sequence repeats | 428,876 | 16,448,977 | 0.71 |
| Low complexity | 77,558 | 3,696,043 | 0.16 |
| Unclassified | 58,853 | 14,590,202 | 0.63 |

**Supplementary Table 3.** **Annotation of** **protein-coding genes in the Siamese (SED) and Burmese (BED) Eld’s deer genomes.**

|  | **SED** | **BED** |
| --- | --- | --- |
| Number of protein-coding genes | 24,913 | 28,831 |
| Total gene length (Mb) | 269 | 413.76 |
| Gene density (%) | 10.81 | 17.80 |
| Total exon number | 170,142 | 345,480 |
| Average number of exons/gene | 6.83 | 9.57 |
| Total exon length (Mb) | 33 | 54.29 |
| Average exon length (bp) | 194 | 196.80 |
| Total intron length (Mb) | 235 | 359.50 |
| Average intron length (bp) | 1,621 | 1,456 |
| BUSCO evaluation (% completeness) | 70.1 | 69 |

**Supplementary Table 4.** **Annotation of non-coding RNAs in the Siamese (SED) and Burmese (BED) Eld’s deer genomes.**

| Type | Average length (bp) | Total length (bp) | Percentage of assembled genome |
| --- | --- | --- | --- |
| *SED* |  |  |  |
| miRNA | 97.89 | 2,576,819 | 0.104 |
| tRNA | 71.91 | 2,271,053 | 0.091 |
| rRNA | 217.97 | 120,758 | 0.005 |
| snRNA | 117.12 | 375,036 | 0.015 |
| *BED* |  |  |  |
| miRNA | 95.28 | 4,902,805 | 0.211 |
| tRNA | 71.29 | 11,531,007 | 0.496 |
| rRNA | 134.30 | 69,969 | 0.003 |
| snRNA | 89.785 | 1,879,477 | 0.081 |

**Supplementary Table 5. Annotation of the complete mitochondrial genomes of Siamese (SED) and Burmese (BED) Eld’s deer.**

| **Name** | **Type** | **SED** | | | | **BED** | | | |
| --- | --- | --- | --- | --- | --- | --- | --- | --- | --- |
|  |  | **Start** | **Stop** | **Strand** | **Length** | **Start** | **Stop** | **Strand** | **Length** |
| D-loop region | NCR | 1 | 774 | – | 774 | 1 | 924 | – | 924 |
| Cytochrome *b* (CYTB) | PCG | 918 | 2051 | – | 1134 | 1068 | 2201 | – | 1134 |
| NADH dehydrogenase subunit 6 (ND6) | PCG | 2128 | 2646 | + | 519 | 2278 | 2796 | + | 519 |
| NADH dehydrogenase subunit 5 (ND5) | PCG | 2654 | 4456 | – | 1803 | 2804 | 4606 | – | 1803 |
| NADH dehydrogenase subunit 4 (ND4) | PCG | 4667 | 6034 | – | 1368 | 4817 | 6184 | – | 1368 |
| NADH dehydrogenase subunit 4L (ND4L) | PCG | 6031 | 6324 | – | 294 | 6181 | 6474 | – | 294 |
| NADH dehydrogenase subunit 3 (ND3) | PCG | 6396 | 6740 | – | 345 | 6546 | 6890 | – | 345 |
| Cytochrome c oxidase subunit 3 (COX3) | PCG | 6811 | 7593 | – | 783 | 6961 | 7743 | – | 783 |
| ATP synthase F0 subunit 6 (ATP6) | PCG | 7599 | 8273 | – | 675 | 7749 | 8423 | – | 675 |
| ATP synthase F0 subunit 8 (ATP8) | PCG | 8240 | 8434 | – | 195 | 8390 | 8584 | – | 195 |
| Cytochrome c oxidase subunit 2 (COX2) | PCG | 8509 | 9189 | – | 681 | 8659 | 9339 | – | 681 |
| Cytochrome c oxidase subunit 1 (COX1) | PCG | 9338 | 10876 | – | 1539 | 9488 | 11026 | – | 1539 |
| NADH dehydrogenase subunit 2 (ND2) | PCG | 11271 | 12302 | – | 1032 | 11421 | 12452 | – | 1032 |
| NADH dehydrogenase subunit 1 (ND1) | PCG | 12517 | 13461 | – | 945 | 12667 | 13611 | – | 945 |
| rrnL | rRNA | 13546 | 15118 | – | 1573 | 13696 | 15269 | – | 1574 |
| rrnS | rRNA | 15184 | 16138 | – | 955 | 15335 | 16289 | – | 955 |
| trnP(cca) | tRNA | 774 | 839 | + | 66 | 924 | 989 | + | 66 |
| trnT(aca) | tRNA | 839 | 908 | – | 70 | 989 | 1058 | – | 70 |
| trnE(gaa) | tRNA | 2056 | 2124 | + | 69 | 2206 | 2274 | + | 69 |
| trnL1(cta) | tRNA | 4457 | 4526 | – | 70 | 4607 | 4676 | – | 70 |
| trnS1(agc) | tRNA | 4528 | 4587 | – | 60 | 4678 | 4737 | – | 60 |
| trnH(cac) | tRNA | 4588 | 4656 | – | 69 | 4738 | 4806 | – | 69 |
| trnR(cga) | tRNA | 6325 | 6393 | – | 69 | 6475 | 6543 | – | 69 |
| trnG(gga) | tRNA | 6741 | 6809 | – | 69 | 6891 | 6959 | – | 69 |
| trnK(aaa) | tRNA | 8436 | 8502 | – | 67 | 8586 | 8652 | – | 67 |
| trnD(gac) | tRNA | 9191 | 9258 | – | 68 | 9341 | 9408 | – | 68 |
| trnS2(tca) | tRNA | 9266 | 9334 | + | 69 | 9416 | 9484 | + | 69 |
| trnY(tac) | tRNA | 10878 | 10946 | + | 69 | 11028 | 11096 | + | 69 |
| trnC(tgc) | tRNA | 10947 | 11014 | + | 68 | 11097 | 11164 | + | 68 |
| trnN(aac) | tRNA | 11047 | 11119 | + | 73 | 11197 | 11269 | + | 73 |
| trnA(gca) | tRNA | 11122 | 11190 | + | 69 | 11272 | 11340 | + | 69 |
| trnW(tga) | tRNA | 11193 | 11260 | – | 68 | 11343 | 11410 | – | 68 |
| trnM(atg) | tRNA | 12303 | 12371 | – | 69 | 12453 | 12521 | – | 69 |
| trnQ(caa) | tRNA | 12374 | 12445 | + | 72 | 12524 | 12595 | + | 72 |
| trnI(atc) | tRNA | 12443 | 12511 | – | 69 | 12593 | 12661 | – | 69 |
| trnL2(tta) | tRNA | 13470 | 13544 | – | 75 | 13620 | 13694 | – | 75 |
| trnV(gta) | tRNA | 15117 | 15183 | – | 67 | 15268 | 15334 | – | 67 |
| trnF(ttc) | tRNA | 16139 | 16207 | – | 69 | 16290 | 16358 | – | 69 |

PCG = protein-coding gene

rRNA = ribosomal RNA

tRNA = transfer RNA

**Supplementary Table 6. List of mitochondrial genomes and the pairwise distances between mitogenome sequences of Siamese Eld’s deer (*Rucervus eldii siamensis*), Burmese Eld’s deer (*Rucervus eldii thamin*) and 28 other cetartiodactyls.**

| **Scientific name** | **Common name** | **Family** | **Accession number** | ***Rucervus eldii siamensis*** | ***Rucervus eldii thamin*** |
| --- | --- | --- | --- | --- | --- |
| *Rucervus eldii siamensis* | Siamese Eld’s deer | Cervidae | Present study |  |  |
| *Rucervus eldii thamin* | Burmese Eld’s deer | Cervidae | Present study | 0.0053 |  |
| *Bos javanicus* | Banteng | Bovidae | NC_012706 | 0.0633 | 0.0631 |
| *Bos taurus* | Cattle | Bovidae | NC_006853 | 0.0647 | 0.0644 |
| *Rucervus eldii hainanus* (Hainan mainland) | Siamese Eld’s deer (Hainan mainland) | Cervidae | HM138200 | 0.0026 | 0.0061 |
| *Rucervus eldii eldii* | Manipur Eld’s deer | Cervidae | KU133959 | 0.0116 | 0.0084 |
| *Rucervus eldii hainanus* (Hainan Island) | Siamese Eld’s deer (Hainan Island) | Cervidae | NC_014701 | 0.0026 | 0.0061 |
| *Elaphurus davidianus* | Père David's deer | Cervidae | NC_018358 | 0.0153 | 0.0137 |
| *Rucervus duvaucelii* | Barasingha | Cervidae | NC_020743 | 0.0195 | 0.0185 |
| *Giraffa camelopardalis* | Giraffe | Giraffidae | NC_024820 | 0.0726 | 0.0712 |
| *Okapia johnstoni* | Okapi | Giraffidae | NC_020730 | 0.0699 | 0.0681 |
| *Moschus chrysogaster* | Alpine musk deer | Moschidae | NC_020093 | 0.0517 | 0.0504 |
| *Moschus moschiferus* | Siberian musk deer | Moschidae | NC_013753 | 0.0552 | 0.0538 |
| *Axis axis* | Chital | Cervidae | NC_020680 | 0.0266 | 0.0261 |
| *Axis porcinus* | Hog deer | Cervidae | NC_020681 | 0.0224 | 0.0235 |
| *Odocoileus virginianus* | White-tailed deer | Cervidae | NC_015247 | 0.0330 | 0.0325 |
| *Rangifer tarandus* | Reindeer | Cervidae | NC_007703 | 0.0340 | 0.0325 |
| *Cervus elaphus* | Red deer | Cervidae | NC_007704 | 0.0190 | 0.0174 |
| *Cervus albirostris* | White-lipped deer | Cervidae | NC_016707 | 0.0161 | 0.0150 |
| *Cervus nippon kopschi* | South China sika deer | Cervidae | NC_016178 | 0.0161 | 0.0145 |
| *Rusa unicolor hainana* | Sambar | Cervidae | NC_031835 | 0.0156 | 0.0145 |
| *Muntiacus muntjak* | Muntjac | Cervidae | NC_004563 | 0.0377 | 0.0372 |
| *Dama dama* | Fallow deer | Cervidae | NC_020700 | 0.0232 | 0.0219 |
| *Muntiacus reevesi* | Reeves' muntjac | Cervidae | NC_004069 | 0.0354 | 0.0340 |
| *Tragulus kanchil* | Lesser mouse-deer | Tragulidae | NC_020753 | 0.1024 | 0.1016 |
| *Rusa alfredi* | Visayan spotted deer | Cervidae | NC_020744 | 0.0253 | 0.0232 |
| *Elaphodus cephalophus* | Tufted deer | Cervidae | NC_008749 | 0.0317 | 0.0314 |
| *Antilocapra americana* | Pronghorn | Antilocapridae | NC_020679 | 0.0594 | 0.0586 |
| *Hippopotamus amphibius* | Hippopotamus | Hippopotamidae | NC_000889 | 0.1297 | 0.1284 |
| *Orcinus orca* | Killer whale | Delphinidae | NC_023889 | 0.1622 | 0.1595 |

**Supplementary Table 7.** **Genome statistics of species used in the comparative genomic analyses.** The list includes the genome statistic information and sources of the eight selected species.

| **Species** | **Common name** | **Assembly size (Gb)** | **Number of protein-coding genes** | **Source** | **Accession number** |
| --- | --- | --- | --- | --- | --- |
| *Rucervus eldii siamensis* | Siamese Eld’s deer | 2.48 | 24,913 | Present study | |
| *Rucervus eldii thamin* | Burmese Eld’s deer | 2.32 | 24,813 | Present study | |
| *Homo sapiens* | Human | 3.1 | 19,813 | Ensembl | GCA_000001405.28 |
| *Cervus hanglu yarkandensis* | Yarkand deer | 2.59 | 22,020 | Ensembl | GCA_010411085.1 |
| *Cervus elaphus* | Red deer | 3.4 | 19.368 | NCBI | MKHE00000000 |
| *Bos taurus* | Cattle | 2.72 | 21,880 | Ensembl | GCA_002263795.2 |
| *Ovis aries* | Sheep | 2.87 | 20,506 | Ensembl | GCA_002742125.1 |
| *Capra hircus* | Goat | 2.92 | 21,361 | Ensembl | GCA_001704415.1 |
| *Moschus moschiferus* | Siberian musk deer | 3.07 | 21,352 | Ensembl | GCA_004024705.2 |
| *Elaphurus davidianus* | Père David’s deer | 2.52 | 20,125 | GigaDB | DOI: 10.5524/100383 |

**Supplementary Table 8. List of 169 species-specific gene clusters of Siamese Eld’s deer (SED) shown in the Venn diagram in Fig. 2a.** The list includes gene cluster name, numbers of proteins found in that cluster, Swiss-Prot ID and GO annotation. The analysis was performed using OrthoVenn2.

| **Cluster name** | **Protein number** | **Swiss-Prot ID** | **GO annotation** |
| --- | --- | --- | --- |
| cluster362 | 30 | P21414 | GO:0019068;P:virion assembly;IEA:InterPro |
| cluster1876 | 15 | Q9XSI3 | GO:0006412;P:translation;IEA:InterPro |
| cluster7210 | 8 | Q24JY1 | GO:0006412;P:translation;IEA:InterPro |
| cluster9172 | 7 | Q3T025 | GO:0002181;P:cytoplasmic translation; IBA:GO_Central |
| cluster11795 | 6 | Q13136 | GO:0007165;P:signal transduction;TAS:ProtInc |
| cluster13553 | 5 | Q576B5 | GO:0006120;P:mitochondrial electron transport,NADH to ubiquinone;IEA:InterPro |
| cluster15181 | 5 | P21414 | GO:0019068;P:virion assembly;IEA:InterPro |
| cluster15183 | 5 | P51991 | GO:0016070;P:RNA metabolic process;TAS:Reactome |
| cluster15184 | 5 | A4FUI2 | GO:0000387;P:spliceosomal snRNP assembly;ISS:UniProtKB |
| cluster15188 | 5 | Q9GZP7 | GO:0019236;P:response to pheromone;IEA:UniProtKB-KW |
| cluster15189 | 5 | P78358 | GO:0005737;C:cytoplasm;IDA:LIFEdb |
| cluster15190 | 5 | Q29432 | GO:0006508;P:proteolysis;IBA:GO_Central |
| cluster17489 | 4 | Q8VEM8 | GO:0035435;P:phosphate ion transmembrane |
| cluster17492 | 4 | Q5E995 | GO:0006412;P:translation;IEA:InterPro |
| cluster17494 | 4 | O03515 | GO:0006119;P:oxidative phosphorylation;IEA:UniProtKB-UniPathway |
| cluster17501 | 4 | Q76I82 | GO:0006412;P:translation;IEA:InterPro |
| cluster20198 | 3 | O15439 | GO:0055085;P:transmembrane transport;IBA:GO_Central |
| cluster20199 | 3 | Q8N7R7 | GO:0007283;P:spermatogenesis;IEA:Ensembl |
| cluster20201 | 3 | P00355 | GO:0006417;P:regulation of translation;IEA:UniProtKB-KW |
| cluster20202 | 3 | P68432 | GO:0006334;P:nucleosome assembly;IBA:GO_Central |
| cluster20204 | 3 | P10273 | GO:0019068;P:virion assembly;IEA:InterPro |
| cluster20205 | 3 | Q24JY1 | GO:0006412;P:translation;IEA:InterPro |
| cluster20206 | 3 | Q3ZCF3 | GO:0031146;P:SCF-dependent proteasomal ubiquitin-dependent protein catabolic process;ISS:UniProtKB |
| cluster20209 | 3 | Q3T025 | GO:0002181;P:cytoplasmic translation;IBA:GO_Central |
| cluster20210 | 3 | Q9JIK5 | GO:0006366;P:transcription by RNA polymerase II;ISS:UniProtKB |
| cluster20211 | 3 | P10096 | GO:0099162;P:regulation of neurotransmitter loading into synaptic vesicle;IDA:SynGO |
| cluster20212 | 3 | P68103 | GO:0006414;P:translational elongation;IBA:GO_Central |
| cluster20214 | 3 | P32969 | GO:0006413;P:translational initiation;TAS:Reactome |
| cluster20217 | 3 | P61077 | GO:0006511;P:ubiquitin-dependent protein catabolic process;IBA:GO_Central |
| cluster20218 | 3 | Q9NQN1 | GO:0007608;P:sensory perception of smell;NAS:UniProtKB |
| cluster20220 | 3 | P48029 | GO:0006936;P:muscle contraction;TAS:ProtInc |
| cluster20223 | 3 | Q56JV9 | GO:0006412;P:translation;IEA:UniProtKB-UniRule |
| cluster20236 | 3 | Q9H343 | GO:0007608;P:sensory perception of smell;NAS:UniProtKB |
| cluster20237 | 3 | P12402 | GO:0031667;P:response to nutrient levels;IBA:GO_Central |
| cluster20241 | 3 | Q6B411 | GO:0008152;P:metabolic process;IEA:UniProtKB-KW |
| cluster23801 | 2 | Q8NGL6 | GO:0007186;P:G protein-coupled receptor signaling pathway;IBA:GO_Central |
| cluster23803 | 2 | P10096 | GO:0099162;P:regulation of neurotransmitter loading into synaptic vesicle;IDA:SynGO |
| cluster23804 | 2 | Q9UJC6 | GO:0006508;P:proteolysis;IDA:BHF-UCL |
| cluster23806 | 2 | Q60887 | GO:0007608;P:sensory perception of smell;ISA:MGI |
| cluster23807 | 2 | C0HL66 | GO:0007140;P:male meiotic nuclear division;IGI:FlyBase |
| cluster23809 | 2 | Q8NGR5 | GO:0007186;P:G protein-coupled receptor signaling pathway;IBA:GO_Central |
| cluster23810 | 2 | P10273 | GO:0019068;P:virion assembly;IEA:InterPro |
| cluster23811 | 2 | Q2KHT7 | GO:0006412;P:translation;IEA:InterPro |
| cluster23812 | 2 | P21414 | GO:0019068;P:virion assembly;IEA:InterPro |
| cluster23813 | 2 | P79402 | GO:0006805;P:xenobiotic metabolic process;IBA:GO_Central |
| cluster23814 | 2 | Q2TBW8 | GO:0006412;P:translation;IEA:InterPro |
| cluster23815 | 2 | P12682 | GO:0034142;P:toll-like receptor 4 signaling pathway;IDA:UniProtKB |
| cluster23816 | 2 | Q8IXS1 | GO:0007165;P:signal transduction;IEA:InterPro |
| cluster23817 | 2 | Q28949 | GO:0006517;P:protein deglycosylation;IBA:GO_Central |
| cluster23818 | 2 | Q8NGL4 | GO:0007608;P:sensory perception of smell;IBA:GO_Central |
| cluster23819 | 2 | Q1JP79 | GO:0034314;P:Arp2/3 complex-mediated actin nucleation;IBA:GO_Central |
| cluster23820 | 2 | Q9NQV7 | GO:0010845;P:positive regulation of reciprocal meiotic recombination;IMP:MGI |
| cluster23821 | 2 | Q9NQV7 | GO:0010845;P:positive regulation of reciprocal meiotic recombination;IMP:MGI |
| cluster23822 | 2 | O15439 | GO:0055085;P:transmembrane transport;IBA:GO_Central |
| cluster23823 | 2 | O15439 | GO:0055085;P:transmembrane transport;IBA:GO_Central |
| cluster23824 | 2 | O15439 | GO:0055085;P:transmembrane transport;IBA:GO_Central |
| cluster23827 | 2 | P26810 | GO:0019068;P:virion assembly;IEA:InterPro |
| cluster23828 | 2 | Q8R2E6 | GO:0019236;P:response to pheromone;IEA:UniProtKB-KW |
| cluster23829 | 2 | Q9UF33 | GO:0070374;P:positive regulation of ERK1 and ERK2 cascade;IBA:GO_Central |
| cluster23832 | 2 | Q86TF6 | GO:0009306;P:proteinsecretion;IBA:GO_Central |
| cluster23833 | 2 | Q2KHT7 | GO:0006412;P:translation;IEA:InterPro |
| cluster23840 | 2 | P82943 | GO:0050729;P:positive regulation of inflammatory response;IBA:GO_Central |
| cluster23841 | 2 | Q9Y3C4 | GO:0002949;P:tRNA threonylcarbamoyladenosine modification;IDA:UniProtKB |
| cluster23842 | 2 | P10272 | GO:0019068;P:virion assembly;IEA:InterPro |
| cluster23845 | 2 | Q60894 | GO:0007608;P:sensory perception of smell;ISA:MGI |
| cluster23846 | 2 | Q2KHT7 | GO:0006412;P:translation;IEA:InterPro |
| cluster23847 | 2 | Q8N7R7 | GO:0007283;P:spermatogenesis;IEA:Ensembl |
| cluster23851 | 2 | Q8IZU3 | GO:0035093;P:spermatogenesis,exchange of chromosomal proteins;IMP:UniProtKB |
| cluster23852 | 2 | P60509 | GO:0005886;C:plasma membrane;IEA:UniProtKB-SubCell |
| cluster23855 | 2 | A0A1B0GWH4 | GO:0061408;P:positive regulation of transcription from RNApolymeraseIIII promoter in response to'&R103&"&S103&"IBA:GO_Central |
| cluster23857 | 2 | Q9U6D3 | GO:0019722;P:calcium-mediated signaling;IEA:InterPro |
| cluster23858 | 2 | Q6PCP5 | GO:0001836;P:release of cytochrome c from mitochondria;IEA:Ensembl |
| cluster23861 | 2 | Q811D2 | GO:0019216;P:regulation of lipid metabolic process;IMP:MGI |
| cluster23864 | 2 | P62261 | GO:1901016;P:regulation of potassiumion transmembrane transporter activity;IEA:Ensembl |
| cluster23865 | 2 | Q58DW0 | GO:0006412;P:translation;IEA:InterPro |
| cluster23866 | 2 | Q3T057 | GO:0006412;P:translation;IEA:InterPro |
| cluster23867 | 2 | Q8N7R7 | GO:0007283;P:spermatogenesis;IEA:Ensembl |
| cluster23868 | 2 | Q9UPS8 | GO:0045599;P:negative rgulation of fat cell differentiation;ISS:UniProtKB |
| cluster23869 | 2 | Q05AK9 | GO:0002181;P:cytoplasmic translation;IBA:GO_Central |
| cluster23870 | 2 | Q3TT38 | GO:0016236;P:macroautophagy;IBA:GO_Central |
| cluster23871 | 2 | P49109 | GO:0050661;F:NADP binding;IEA:InterPro |
| cluster23873 | 2 | Q29407 | GO:0007283;P:spermatogenesis;IEA:UniProtKB-KW |
| cluster23874 | 2 | P08361 | GO:0019068;P:virion assembly;IEA:InterPro |
| cluster23875 | 2 | Q2HJ21 | GO:0071157;P:negative regulation of cell cycle arrest;IEA:InterPro |
| cluster23876 | 2 | Q2YDE4 | GO:0043161;P:proteasome-mediated ubiquitin-dependent protein catabolic process;IBA:GO_Central |
| cluster23877 | 2 | Q76I82 | GO:0006412;P:translation;IEA:InterPro |
| cluster23878 | 2 | P60987 | GO:0010628;P:positive regulation of gene expression;IEA:Ensembl |
| cluster23879 | 2 | Q2HJI8 | GO:0006904;P:vesicle docking involved in exocytosis;IBA:GO_Central |
| cluster23882 | 2 | P62935 | GO:0045069;P:regulation of viral genome replication;ISS:UniProtKB |
| cluster23883 | 2 | P08183 | GO:0055085;P:transmembrane transport;TAS:Reactome |
| cluster23885 | 2 | Q9TTC0 | GO:0019062;P:virion attachment to host cell;IEA:UniProtKB-KW |
| cluster23886 | 2 | Q6IF82 | GO:0007186;P:G protein-coupled receptor signaling pathway;IBA:GO_Central |
| cluster23887 | 2 | Q2Q421 | GO:0006954;P:inflammatory response;IEA:UniProtKB-KW |
| cluster23888 | 2 | O76100 | GO:0007186;P:G protein-coupled receptor signaling pathway;IBA:GO_Central |
| cluster23889 | 2 | Q15622 | GO:0007608;P:sensory perception of smell;NAS:UniProtKB |
| cluster23890 | 2 | O14581 | GO:0007186;P:G protein-coupled receptor signaling pathway;IBA:GO_Central |
| cluster23891 | 2 | Q6IFN5 | GO:0007186;P:G protein-coupled receptor signaling pathway;IBA:GO_Central |
| cluster23892 | 2 | P34925 | GO:1904953;P:Wnt signaling pathway involved in midbrain dopaminergic neuron differentiation;ISS:ParkinsonsUK-UCL |
| cluster23893 | 2 | Q3SZF2 | GO:0016192;P:vesicle-mediated transport;IBA:GO_Central |
| cluster23894 | 2 | Q6ZQ12 | GO:0034454;P:microtubule anchoring at centrosome;IBA:GO_Central |
| cluster23895 | 2 | P0C646 | GO:0004984;F:olfactory receptor activity;IBA:GO_Central |
| cluster23896 | 2 | Q8TCB6 | GO:0004984;F:olfactory receptor activity;IBA:GO_Central |
| cluster23897 | 2 | Q8NGK2 | GO:0050890;P:cognition;IMP:UniProtKB |
| cluster23898 | 2 | Q9XSI3 | GO:0006412;P:translation;IEA:InterPro |
| cluster23899 | 2 | O95918 | GO:0007618;P:mating;TAS:ProtInc |
| cluster23900 | 2 | Q9GZL5 | GO:0004984;F:olfactory receptor activity;IBA:GO_Central |
| cluster23901 | 2 | P13752 | GO:0001916;P:positive regulation of Tcell mediated cytotoxicity;IBA:GO_Central |
| cluster23902 | 2 | P13753 | GO:0001916;P:positive regulation of Tcell mediated cytotoxicity;IBA:GO_Central |
| cluster23903 | 2 | Q96KK5 | GO:0006325;P:chromatin organization;IBA:GO_Central |
| cluster23904 | 2 | P13753 | GO:0001916;P:positive regulation of Tcell mediated cytotoxicity;IBA:GO_Central |
| cluster23905 | 2 | P13753 | GO:0001916;P:positive regulation of Tcell mediated cytotoxicity;IBA:GO_Central |
| cluster23906 | 2 | Q7Z3L3 | GO:0045892;P:negative regulation of transcription,DNA-templated;IEA:InterPro |
| cluster23907 | 2 | Q8NGB8 | GO:0007186;P:Gprotein-coupled receptor signaling pathway;IBA:GO_Central |
| cluster23908 | 2 | A6NET4 | GO:0007608;P:sensory perception of smell;IBA:GO_Central |
| cluster23910 | 2 | Q58DW5 | GO:0006412;P:translation;IEA:InterPro |
| cluster23912 | 2 | Q8NH59 | GO:0004984;F:olfactory receptor activity;IBA:GO_Central |
| cluster23913 | 2 | Q8NGI8 | GO:0007608;P:sensory perception of smell;IBA:GO_Central |
| cluster23914 | 2 | B4DYI2 | GO:0007283;P:spermatogenesis;IEA:UniProtKB-KW |
| cluster23919 | 2 | Q17Q91 | GO:0005758;C:mitochondrial intermembrane space;IEA:UniProtKB-SubCell |
| cluster23920 | 2 | Q29432 | GO:0006508;P:proteolysis;IBA:GO_Central |
| cluster23921 | 2 | Q29432 | GO:0006508;P:proteolysis;IBA:GO_Central |
| cluster23922 | 2 | B4DX44 | GO:0046872;F:metalion binding;IEA:UniProtKB-KW |

**Supplementary Table 9.** **List of significant functionally enriched species-specific gene clusters of Siamese Eld’s deer (SED).** The list shows enriched GO terms and GO categories of SED-specific gene clusters, computed by OrthoVenn2. The numbers of species-specific genes included in the enriched pathways are shown.

| **GO ID** | **GO category** | **GO term** | **Count** | ***p*-value** |
| --- | --- | --- | --- | --- |
| GO:0019068 | biological_process | virion assembly | 8 | 1.12E-14 |
| GO:0006412 | biological_process | translation | 15 | 6.79E-13 |
| GO:0001916 | biological_process | positive regulation of T cell mediated cytotoxicity | 4 | 2.11E-06 |
| GO:0007608 | biological_process | sensory perception of smell | 8 | 9.72E-06 |
| GO:0055085 | biological_process | transmembrane transport | 5 | 8.91E-05 |
| GO:0007186 | biological_process | G-protein-coupled receptor signaling pathway | 7 | 9.00E-05 |
| GO:0010845 | biological_process | positive regulation of reciprocal meiotic recombination | 2 | 0.000571 |
| GO:0002181 | biological_process | cytoplasmic translation | 3 | 0.000885 |
| GO:0006508 | biological_process | proteolysis | 4 | 0.003579 |
| GO:0019236 | biological_process | response to pheromone | 2 | 0.004813 |

**Supplementary Table 10. List of 108 species-specific gene clusters of Burmese Eld’s deer (BED) shown in the Venn diagram in Fig. 2a.** The list includes gene cluster name, numbers of proteins found in that cluster, Swiss-Prot ID and GO annotation. The analysis was performed using OrthoVenn2.

| **Cluster name** | **Protein number** | **Swiss-Prot ID** | **GO annotation** |
| --- | --- | --- | --- |
| cluster30 | 79 | O00370 | GO:0032197; P:transposition, RNA-mediated; IMP:UniProtKB |
| cluster31 | 78 | P10272 | GO:0019068; P:virion assembly; IEA:InterPro |
| cluster129 | 46 | P11369 | GO:0006310; P:DNA recombination; IEA:UniProtKB-KW |
| cluster185 | 40 | O00370 | GO:0032197; P:transposition, RNA-mediated; IMP:UniProtKB |
| cluster186 | 40 | N/A | N/A |
| cluster1055 | 19 | O00370 | GO:0032197; P:transposition, RNA-mediated; IMP:UniProtKB |
| cluster1406 | 17 | O00370 | GO:0032197; P:transposition, RNA-mediated; IMP:UniProtKB |
| cluster1407 | 17 | N/A | N/A |
| cluster2250 | 14 | N/A | N/A |
| cluster3931 | 11 | N/A | N/A |
| cluster3932 | 11 | N/A | N/A |
| cluster5931 | 9 | O02751 | GO:0005634; C:nucleus; IEA:UniProtKB-SubCell |
| cluster7522 | 8 | N/A | N/A |
| cluster7523 | 8 | N/A | N/A |
| cluster7524 | 8 | Q8TE20 | GO:0008277; P:regulation of G protein-coupled receptor signaling pathway; TAS:ProtInc |
| cluster7525 | 8 | O02751 | GO:0005634; C:nucleus; IEA:UniProtKB-SubCell |
| cluster9617 | 7 | Q9UN81 | GO:0032197; P:transposition, RNA-mediated; IDA:UniProtKB |
| cluster9637 | 7 | N/A | N/A |
| cluster12457 | 6 | A2VDU2 | GO:0038203; P:TORC2 signaling; IBA:GO_Central |
| cluster12459 | 6 | N/A | N/A |
| cluster12460 | 6 | N/A | N/A |
| cluster12461 | 6 | O92815 | GO:0006310; P:DNA recombination; IEA:UniProtKB-KW |
| cluster15668 | 5 | O00370 | GO:0032197; P:transposition, RNA-mediated; IMP:UniProtKB |
| cluster16113 | 5 | O00370 | GO:0032197; P:transposition, RNA-mediated; IMP:UniProtKB |
| cluster16127 | 5 | P03359 | GO:0019068; P:virion assembly; IEA:InterPro |
| cluster16128 | 5 | N/A | N/A |
| cluster16129 | 5 | N/A | N/A |
| cluster16130 | 5 | N/A | N/A |
| cluster16131 | 5 | N/A | N/A |
| cluster16132 | 5 | Q7Z5H5 | GO:0019236; P:response to pheromone; IEA:UniProtKB-KW |
| cluster16133 | 5 | P21436 | GO:0019062; P:virion attachment to host cell; IEA:UniProtKB-KW |
| cluster19177 | 4 | N/A | N/A |
| cluster19178 | 4 | N/A | N/A |
| cluster19179 | 4 | Q53H47 | GO:0031297; P:replication fork processing; IMP:UniProtKB |
| cluster19180 | 4 | Q588U8 | GO:0005634; C:nucleus; IEA:UniProtKB-SubCell |
| cluster19181 | 4 | N/A | N/A |
| cluster19182 | 4 | N/A | N/A |
| cluster19185 | 4 | N/A | N/A |
| cluster19186 | 4 | N/A | N/A |
| cluster21031 | 3 | P08548 | GO:0003964; F:RNA-directed DNA polymerase activity; IEA:UniProtKB-KW |
| cluster22797 | 3 | P30205 | GO:0005044; F:scavenger receptor activity; IEA:InterPro |
| cluster22798 | 3 | N/A | N/A |
| cluster22799 | 3 | N/A | N/A |
| cluster22800 | 3 | N/A | N/A |
| cluster22801 | 3 | N/A | N/A |
| cluster22802 | 3 | N/A | N/A |
| cluster22803 | 3 | O15439 | GO:0055085; P:transmembrane transport; IBA:GO_Central |
| cluster22804 | 3 | O02751 | GO:0005634; C:nucleus; IEA:UniProtKB-SubCell |
| cluster22805 | 3 | O02751 | GO:0005634; C:nucleus; IEA:UniProtKB-SubCell |
| cluster22806 | 3 | N/A | N/A |
| cluster22807 | 3 | N/A | N/A |
| cluster22808 | 3 | O02751 | GO:0005634; C:nucleus; IEA:UniProtKB-SubCell |
| cluster22809 | 3 | P11369 | GO:0006310; P:DNA recombination; IEA:UniProtKB-KW |
| cluster22810 | 3 | O00370 | GO:0032197; P:transposition, RNA-mediated; IMP:UniProtKB |
| cluster22811 | 3 | N/A | N/A |
| cluster22812 | 3 | N/A | N/A |
| cluster22813 | 3 | N/A | N/A |
| cluster22814 | 3 | N/A | N/A |
| cluster22815 | 3 | P11369 | GO:0006310; P:DNA recombination; IEA:UniProtKB-KW |
| cluster22816 | 3 | Q8NGP0 | GO:0007186; P:G protein-coupled receptor signaling pathway; IBA:GO_Central |
| cluster22817 | 3 | N/A | N/A |
| cluster23044 | 3 | N/A | N/A |
| cluster23799 | 2 | N/A | N/A |
| cluster31421 | 2 | P31622 | GO:0039702; P:viral budding via host ESCRT complex; IEA:UniProtKB-KW |
| cluster31422 | 2 | Q53H47 | GO:0031297; P:replication fork processing; IMP:UniProtKB |
| cluster31423 | 2 | N/A | N/A |
| cluster31424 | 2 | N/A | N/A |
| cluster31425 | 2 | Q588U8 | GO:0005634; C:nucleus; IEA:UniProtKB-SubCell |
| cluster31426 | 2 | N/A | N/A |
| cluster31427 | 2 | N/A | N/A |
| cluster31428 | 2 | N/A | N/A |
| cluster31429 | 2 | Q32L59 | GO:0016021; C:integral component of membrane; IEA:UniProtKB-KW |
| cluster31430 | 2 | N/A | N/A |
| cluster31431 | 2 | O02751 | GO:0005634; C:nucleus; IEA:UniProtKB-SubCell |
| cluster31432 | 2 | P13184 | GO:0002082; P:regulation of oxidative phosphorylation; IBA:GO_Central |
| cluster31433 | 2 | N/A | N/A |
| cluster31434 | 2 | N/A | N/A |
| cluster31435 | 2 | P08548 | GO:0003964; F:RNA-directed DNA polymerase activity; IEA:UniProtKB-KW |
| cluster31436 | 2 | Q588U8 | GO:0005634; C:nucleus; IEA:UniProtKB-SubCell |
| cluster31437 | 2 | N/A | N/A |
| cluster31438 | 2 | P11369 | GO:0006310; P:DNA recombination; IEA:UniProtKB-KW |
| cluster31439 | 2 | N/A | N/A |
| cluster31440 | 2 | N/A | N/A |
| cluster31441 | 2 | O02751 | GO:0005634; C:nucleus; IEA:UniProtKB-SubCell |
| cluster31442 | 2 | P11369 | GO:0006310; P:DNA recombination; IEA:UniProtKB-KW |
| cluster31443 | 2 | A6NL08 | GO:0004984; F:olfactory receptor activity; IBA:GO_Central |
| cluster31444 | 2 | N/A | N/A |
| cluster31445 | 2 | N/A | N/A |
| cluster31446 | 2 | P30205 | GO:0005044; F:scavenger receptor activity; IEA:InterPro |
| cluster31447 | 2 | Q8NGT0 | GO:0004984; F:olfactory receptor activity; IBA:GO_Central |
| cluster31448 | 2 | N/A | N/A |
| cluster31449 | 2 | N/A | N/A |
| cluster31450 | 2 | O00370 | GO:0032197; P:transposition,RNA-mediated; IMP:UniProtKB |
| cluster31451 | 2 | N/A | N/A |
| cluster31452 | 2 | P31623 | GO:0044826; P:viral genome integration into host DNA; IEA:UniProtKB-KW |
| cluster31453 | 2 | Q8TE69 | N/A |
| cluster31454 | 2 | N/A | N/A |
| cluster31455 | 2 | Q8NGL6 | GO:0007186; P:G protein-coupled receptor signaling pathway; IBA:GO_Central |
| cluster31456 | 2 | P08548 | GO:0003964; F:RNA-directed DNA polymerase activity; IEA:UniProtKB-KW |
| cluster31457 | 2 | N/A | N/A |
| cluster31458 | 2 | N/A | N/A |
| cluster31475 | 2 | N/A | N/A |
| cluster31476 | 2 | N/A | N/A |
| cluster31477 | 2 | N/A | N/A |
| cluster31478 | 2 | P30205 | GO:0005044; F:scavenger receptor activity; IEA:InterPro |
| cluster31479 | 2 | N/A | N/A |
| cluster32148 | 2 | N/A | N/A |
| cluster32149 | 2 | O00370 | GO:0032197; P:transposition,RNA-mediated; IMP:UniProtKB |

**Supplementary Table 11. List of significant functionally enriched species-specific gene clusters of Burmese Eld’s deer (BED).** The list shows enriched GO terms and GO categories of SED-specific gene clusters, computed by OrthoVenn2. The numbers of species-specific genes included in the enriched pathways are shown.

| **GO ID** | **GO category** | **GO term** | **Number of cluster** | ***p*-value** |
| --- | --- | --- | --- | --- |
| GO:0032197 | biological_process | transposition, RNA-mediated | 10 | 1.40E-26 |
| GO:0006310 | biological_process | DNA recombination | 6 | 1.24E-14 |
| GO:0005634 | cellular_component | nucleus | 10 | 1.18E-13 |
| GO:0003964 | molecular_function | RNA-directed DNA polymerase activity | 3 | 4.06E-09 |
| GO:0005044 | molecular_function | scavenger receptor activity | 3 | 1.81E-06 |
| GO:0031297 | biological_process | replication fork processing | 2 | 7.20E-05 |
| GO:0019068 | biological_process | virion assembly | 2 | 0.000529 |

**Supplementary Table 12. Positively selected genes in Eld’s deer [*Rucervus eldii siamensis* (SED) and *Rucervus eldii thamin* (BED)] by setting other Cervidae species (*Cervus elaphus*, *Cervus hanglu yarkandensis* and *Elaphurus davidianus*) as a background.** The analysis was performed using the PosiGene pipeline and the candidate genes detected as positively selected were considered at *p* < 0.05 for False Discovery Rate (FDR) and ω > 1.

| **Gene name** | **Gene ID** | **FDR** | **ω** |
| --- | --- | --- | --- |
| *SED and BED clade* |  |  |  |
| Interleukin 12 receptor subunit beta 1 | *IL12RB1* | 1.71E-10 | 9.55364 |
| Unconventional SNARE in the ER 1 | *USE1* | 3.37E-08 | 15.55911 |
| Calpain 13 | *CAPN13* | 2.85E-06 | 4.370554 |
| Tetratricopeptide repeat domain 21A | *TTC21A* | 0.00097 | 2.443428 |
| RNA polymerase I subunit G | *POLR1G* | 0.001054 | 9.09558 |
| StAR related lipid transfer domain containing 3 | *STARD3* | 0.001269 | 6.262225 |
| RB binding protein 6, ubiquitin ligase | *RBBP6* | 0.013178 | 1.167605 |
| Transmembrane protein 208 | *TMEM208* | 0.014558 | 6.50349 |
| Angel homolog 1 | *ANGEL1* | 0.015242 | 2.131508 |
| HPS1 biogenesis of lysosomal organelles complex 3 subunit 1 | *HPS1* | 0.015242 | 5.438642 |
| G protein signaling modulator 2 | *GPSM2* | 0.018889 | 4.853368 |
| Integrin subunit alpha L | *ITGAL* | 0.018889 | 2.634004 |
| DnaJ heat shock protein family (Hsp40) member B7 | *DNAJB7* | 0.019363 | 17.13499 |
| Coiled-coil domain containing 89 | *CCDC89* | 0.025222 | 1.308103 |
| Popeye domain containing 2 | *POPDC2* | 0.026208 | 2.368116 |
| Alpha-2-glycoprotein 1, zinc-binding | *AZGP1* | 0.030037 | 2.359738 |
| Ligand of numb-protein X 2 | *LNX2* | 0.038519 | 1.063686 |
| *SED clade* |  |  |  |
| BAH domain and coiled-coil containing 1 | *BAHCC1* | 1.67E-12 | 3.16316 |
| Ladybird homeobox 1 | *LBX1* | 2.83E-05 | 14.17929 |
| DEAD/H-box helicase 11 | *DDX11* | 3.61E-05 | 5.081614 |
| Zinc finger protein 395 | *ZNF395* | 0.000103 | 2.341983 |
| TEA domain transcription factor 3 | *TEAD3* | 0.000122 | 20.10915 |
| IQ motif containing B1 | *IQCB1* | 0.000254 | 2.07535 |
| Centrosomal protein 55 | *CEP55* | 0.000269 | 5.043702 |
| Sad1 and UNC84 domain containing 2 | *SUN2* | 0.000957 | 3.876567 |
| Nuclear receptor subfamily 2 group E member 3 | *NR2E3* | 0.001324 | 2.84634 |
| RIO kinase 1 | *RIOK1* | 0.01151 | 2.732608 |
| *BED clade* |  |  |  |
| Cytosolic thiouridylase subunit 2 | *CTU2* | 5.75E-09 | 12.62876 |
| Apurinic/apyrimidinic endodeoxyribonuclease 2 | *APEX2* | 4.99E-07 | 10.17226 |
| CACN subunit beta associated regulatory protein | *CBARP* | 9.47E-07 | 3.446078 |
| Phosphatidylinositol-4-phosphate 5-kinase type 1 gamma | *PIP5K1C* | 1.33E-06 | 6.35364 |
| GA binding protein transcription factor subunit beta 2 | *GABPB2* | 7.98E-05 | 4.518385 |
| SRP receptor subunit alpha | *SRPRA* | 8.82E-05 | 1.776244 |
| MICAL like 2 | *MICALL2* | 0.001581 | 2.604642 |
| ArfGAP with RhoGAP domain, ankyrin repeat and PH domain 1 | *ARAP1* | 0.003165 | 1.002873 |
| ERCC excision repair 2, TFIIH core complex helicase subunit | *ERCC2* | 0.006899 | 1.96803 |
| Cleavage stimulation factor subunit 3 | *CSTF3* | 0.009628 | 2.73726 |
| CD248 molecule | *CD248* | 0.028494 | 3.026099 |
| Twinfilin actin binding protein 1 | *TWF1* | 0.028565 | 2.89821 |

**Supplementary Table 13. Significant GO term enrichment of positively selected genes found in both SED (*Rucervus eldii siamensis*) and BED (*Rucervus eldii thamin*) and only in SED in biological processes, molecular function, and cellular component by setting other Cervidae species (*Cervus elaphus*, *Cervus hanglu yarkandensis*, *Elaphurus davidianus*) as a background.** The analysis was performed using the Database for Annotation, Visualization and Integrated Discovery (DAVID). Significantly enriched pathways were considered at *p* < 0.05.

| **Category** | **Term** | ***p*-value** | **Genes** |
| --- | --- | --- | --- |
| *SED and BED clade* |  |  |  |
| GOTERM_BP_DIRECT | GO:0001916~positive regulation of T cell mediated cytotoxicity | 0.020835 | *AZGP1*, *IL12RB1* |
| GOTERM_CC_DIRECT | GO:0009897~external side of plasma membrane | 0.048349464 | *AZGP1*, *ITGAL*, *IL12RB1* |
| *SED clade* |  |  |  |
| GOTERM_BP_DIRECT | GO:0006357~regulation of transcription from RNA polymerase II promoter | 0.028328615 | *ZNF395*, *LBX1*, *NR2E3*, *TEAD3* |
| GOTERM_MF_DIRECT | GO:0003700~transcription factor activity, sequence-specific DNA binding | 0.027171766 | *ZNF395*, *NR2E3*, *TEAD3* |

**Supplementary Table 14. Sample list Siamese (SED) and Burmese Eld’s deer (BED) used in this study.** The list includes the 35 SED and 49 BED whole blood and muscle specimens that were used for *de novo* assembly and RAD sequencing.

| **Sample ID** | **Animal ID** | **Species** | **Sex** | **Location** | **Age (y)** | **Specimen** | **Remarks** |
| --- | --- | --- | --- | --- | --- | --- | --- |
| ED1MTh | 97 | SED | Male | Khao Kheow Open Zoo, ZPOT | 7 | Whole blood | RADseq |
| ED2MTh | 95 | SED | Male | Khao Kheow Open Zoo, ZPOT | 7 | Whole blood | RADseq |
| ED3MTh | B6 | SED | Male | Ubon Ratchathani Zoo, ZPOT | 7 | Whole blood | *De novo* genome assembly, RADseq |
| ED4MTh | 65 | SED | Male | Ubon Ratchathani Zoo, ZPOT | 17 | Whole blood | RADseq |
| ED5MTh | 92 | SED | Male | Ubon Ratchathani Zoo, ZPOT | 9 | Whole blood | RADseq |
| ED6MMy | 54/1 | BED | Male | Khao Kheow Open Zoo, ZPOT | N/A | Whole blood | RADseq |
| ED7FMy | K189 | BED | Female | Khao Kheow Open Zoo, ZPOT | N/A | Whole blood | RADseq |
| ED8FMy | K213 | BED | Female | Khao Kheow Open Zoo, ZPOT | N/A | Whole blood | RADseq |
| ED9MTh | 01/56 | SED | Male | Nakhon Ratchasima Zoo, ZPOT | 9 | Whole blood | RADseq |
| ED10MTh | 86 | SED | Male | Nakhon Ratchasima Zoo, ZPOT | 11 | Whole blood | RADseq |
| ED11MTh | 01/58 | SED | Male | Nakhon Ratchasima Zoo, ZPOT | 7 | Whole blood | RADseq |
| ED12MTh | 6005 | SED | Male | Ubon Ratchathani Zoo, ZPOT | 5 | Whole blood | RADseq |
| ED13MMy | M8 | BED | Male | Khao Kheow Open Zoo, ZPOT | N/A | Whole blood | RADseq |
| ED14MMy | 55/11 | BED | Male | Khao Kheow Open Zoo, ZPOT | 10 | Whole blood | *De novo* genome assembly, RADseq |
| ED15MMy | K205 | BED | Male | Khao Kheow Open Zoo, ZPOT | N/A | Whole blood | RADseq |
| ED16MMy | K215 | BED | Male | Khao Kheow Open Zoo, ZPOT | N/A | Whole blood | RADseq |
| ED17FMy | K118 | BED | Female | Khao Kheow Open Zoo, ZPOT | N/A | Whole blood | RADseq |
| ED18MMy | K216 | BED | Male | Khao Kheow Open Zoo, ZPOT | N/A | Whole blood | RADseq |
| ED19FMy | Pagae | BED | Female | Khao Kheow Open Zoo, ZPOT | N/A | Whole blood | RADseq |
| ED31MMy | M4 | BED | Male | Khao Kheow Open Zoo, ZPOT | 11 | Whole blood | RADseq |
| ED32FMy | K135 | BED | Female | Khao Kheow Open Zoo, ZPOT | N/A | Whole blood | RADseq |
| ED33MMy | 56/2 | BED | Male | Khao Kheow Open Zoo, ZPOT | N/A | Whole blood | RADseq |
| ED34FMy | 64E01 | BED | Female | Huai Kha Khaeng Wildlife Breeding Center, DNP | 1 | Whole blood | RADseq |
| ED35MMy | 64E02 | BED | Male | Huai Kha Khaeng Wildlife Breeding Center, DNP | 1 | Whole blood | RADseq |
| ED36FMy | 64E03 | BED | Female | Huai Kha Khaeng Wildlife Breeding Center, DNP | 1 | Whole blood | RADseq |
| ED37FMy | 64E04 | BED | Female | Huai Kha Khaeng Wildlife Breeding Center, DNP | 1 | Whole blood | RADseq |
| ED38FMy | 64E05 | BED | Female | Huai Kha Khaeng Wildlife Breeding Center, DNP | 1 | Whole blood | RADseq |
| ED39MMy | 64E06 | BED | Male | Huai Kha Khaeng Wildlife Breeding Center, DNP | 1 | Whole blood | RADseq |
| ED40MMy | 64E07 | BED | Male | Huai Kha Khaeng Wildlife Breeding Center, DNP | 1 | Whole blood | RADseq |
| ED41MMy | 64E08 | BED | Male | Huai Kha Khaeng Wildlife Breeding Center, DNP | 1 | Whole blood | RADseq |
| ED42FMy | 64E09 | BED | Female | Huai Kha Khaeng Wildlife Breeding Center, DNP | 1 | Whole blood | RADseq |
| ED43FMy | 64E10 | BED | Female | Huai Kha Khaeng Wildlife Breeding Center, DNP | 1 | Whole blood | RADseq |
| ED44MMy | 64E11 | BED | Male | Huai Kha Khaeng Wildlife Breeding Center, DNP | 1 | Whole blood | RADseq |
| ED45FMy | 64E12 | BED | Female | Huai Kha Khaeng Wildlife Breeding Center, DNP | 1 | Whole blood | RADseq |
| ED46FMy | 64E13 | BED | Female | Huai Kha Khaeng Wildlife Breeding Center, DNP | 1 | Whole blood | RADseq |
| ED47MMy | 64E14 | BED | Male | Huai Kha Khaeng Wildlife Breeding Center, DNP | 1 | Whole blood | RADseq |
| ED48FMy | 64E15 | BED | Female | Huai Kha Khaeng Wildlife Breeding Center, DNP | 1 | Whole blood | RADseq |
| ED49MMy | 64E16 | BED | Male | Huai Kha Khaeng Wildlife Breeding Center, DNP | 1 | Whole blood | RADseq |
| ED50MMy | 64E17 | BED | Male | Huai Kha Khaeng Wildlife Breeding Center, DNP | 1 | Whole blood | RADseq |
| ED51MMy | 64E18 | BED | Male | Huai Kha Khaeng Wildlife Breeding Center, DNP | 1 | Whole blood | RADseq |
| ED52MMy | 64E19 | BED | Male | Huai Kha Khaeng Wildlife Breeding Center, DNP | 1 | Whole blood | RADseq |
| ED53FMy | 64E20 | BED | Female | Huai Kha Khaeng Wildlife Breeding Center, DNP | 1 | Whole blood | RADseq |
| ED54FMy | 64E21 | BED | Female | Huai Kha Khaeng Wildlife Breeding Center, DNP | 1 | Whole blood | RADseq |
| ED55FMy | 64E22 | BED | Female | Huai Kha Khaeng Wildlife Breeding Center, DNP | 1 | Whole blood | RADseq |
| ED56MMy | 64E25 | BED | Male | Huai Kha Khaeng Wildlife Breeding Center, DNP | 1 | Whole blood | RADseq |
| ED57FMy | 64E26 | BED | Female | Huai Kha Khaeng Wildlife Breeding Center, DNP | 1 | Whole blood | RADseq |
| ED58FMy | 64E27 | BED | Female | Huai Kha Khaeng Wildlife Breeding Center, DNP | 1 | Whole blood | RADseq |
| ED59MMy | 64E28 | BED | Male | Huai Kha Khaeng Wildlife Breeding Center, DNP | 1 | Whole blood | RADseq |
| ED60MMy | 64E29 | BED | Male | Huai Kha Khaeng Wildlife Breeding Center, DNP | 1 | Whole blood | RADseq |
| ED61FMy | 64E30 | BED | Female | Huai Kha Khaeng Wildlife Breeding Center, DNP | 1 | Whole blood | RADseq |
| ED62FMy | 64E31 | BED | Female | Huai Kha Khaeng Wildlife Breeding Center, DNP | 1 | Whole blood | RADseq |
| ED63MMy | 64E32 | BED | Male | Huai Kha Khaeng Wildlife Breeding Center, DNP | 1 | Whole blood | RADseq |
| ED64MMy | 58/1 | BED | Male | Khao Kheow Open Zoo, ZPOT | N/A | Whole blood | RADseq |
| ED65MMy | 61/1 | BED | Male | Khao Kheow Open Zoo, ZPOT | N/A | Whole blood | RADseq |
| ED66MMy | 69/493 | BED | Male | Nakhon Ratchasima Zoo, ZPOT | N/A | Whole blood | RADseq |
| ED67MMy | 566 | BED | Male | Nakhon Ratchasima Zoo, ZPOT | N/A | Whole blood | RADseq |
| ED68MTh | 01/61 | SED | Male | Ubon Ratchathani Zoo, ZPOT | 4 | Whole blood | RADseq |
| ED69MTh | B3 | SED | Male | Ubon Ratchathani Zoo, ZPOT | 4 | Whole blood | RADseq |
| ED70MTh | 03/61 | SED | Male | Ubon Ratchathani Zoo, ZPOT | N/A | Whole blood | RADseq |
| ED71MTh | 6001 | SED | Male | Ubon Ratchathani Zoo, ZPOT | N/A | Whole blood | RADseq |
| ED72MMy | 60/1 | BED | Male | Khao Kheow Open Zoo, ZPOT | N/A | Whole blood | RADseq |
| ED73MMy | M10 | BED | Male | Khao Kheow Open Zoo, ZPOT | 14 | Whole blood | RADseq |
| ED74MTh | THBM61/06 | SED | Male | Chulabhorn Wildlife Breeding Center, DNP | 6 | Whole blood | RADseq |
| ED75FTh | THA00187 | SED | Female | Chulabhorn Wildlife Breeding Center, DNP | 2 | Whole blood | RADseq |
| ED76FTh | THA500025 | SED | Female | Chulabhorn Wildlife Breeding Center, DNP | 5 | Whole blood | RADseq |
| ED77FTh | THA500027 | SED | Female | Chulabhorn Wildlife Breeding Center, DNP | 5 | Whole blood | RADseq |
| ED78MTh | BM61/13 | SED | Male | Chulabhorn Wildlife Breeding Center, DNP | 5 | Whole blood | RADseq |
| ED79FTh | THA500024 | SED | Female | Chulabhorn Wildlife Breeding Center, DNP | 4 | Whole blood | RADseq |
| ED80FTh | THA500021 | SED | Female | Chulabhorn Wildlife Breeding Center, DNP | 3 | Whole blood | RADseq |
| ED81MTh | THA500022 | SED | Male | Chulabhorn Wildlife Breeding Center, DNP | 4 | Whole blood | RADseq |
| ED82MTh | THA001808 | SED | Male | Chulabhorn Wildlife Breeding Center, DNP | 4 | Whole blood | RADseq |
| ED83FTh | THA001809 | SED | Female | Chulabhorn Wildlife Breeding Center, DNP | 3 | Whole blood | RADseq |
| ED84FTh | THA001810 | SED | Female | Chulabhorn Wildlife Breeding Center, DNP | 4 | Whole blood | RADseq |
| ED85MTh | THA001811 | SED | Male | Chulabhorn Wildlife Breeding Center, DNP | 2 | Whole blood | RADseq |
| ED86FTh | THA001812 | SED | Female | Chulabhorn Wildlife Breeding Center, DNP | 5 | Whole blood | RADseq |
| ED87FTh | DNPTHA500080 | SED | Female | Banglamung Wildlife Breeding Center, DNP | 2 | Whole blood | RADseq |
| ED88FTh | DNPTHA500081 | SED | Female | Banglamung Wildlife Breeding Center, DNP | 2 | Whole blood | RADseq |
| ED89MTh | DNPTHA500082 | SED | Male | Banglamung Wildlife Breeding Center, DNP | 2 | Whole blood | RADseq |
| ED90FTh | DNPTHA500083 | SED | Female | Banglamung Wildlife Breeding Center, DNP | 3 | Whole blood | RADseq |
| ED91FTh | DNPTHA500084 | SED | Female | Banglamung Wildlife Breeding Center, DNP | 3 | Whole blood | RADseq |
| ED92FTh | DNPTHA500085 | SED | Female | Banglamung Wildlife Breeding Center, DNP | 3 | Whole blood | RADseq |
| ED93MTh | DNPTHA500086 | SED | Male | Banglamung Wildlife Breeding Center, dNP | 3 | Whole blood | RADseq |
| ED94FTh | DNPTHA500087 | SED | Female | Banglamung Wildlife Breeding Center, DNP | 3 | Whole blood | RADseq |
| ED95MTh | BM61/05 | SED | Male | Banglamung Wildlife Breeding Center, DNP | 8 | Thigh muscle | RADseq |

**Supplementary Table 15. Statistics of RADseq data and mapped reads across 84 Eld’s deer individuals.** Total reads, total mapped reads, and percentage of mapped rates of 35 Siamese Eld’s deer (SED) and 49 Burmese Eld’s deer (BED) RADseq samples are shown. Reads were aligned to the SED reference genome assembly.

| **Number** | **Sample ID** | **Total of paired-end reads** | **Total mapped reads** | **Percentage of overall alignment rate** |
| --- | --- | --- | --- | --- |
| 1 | ED1MTh | 38,385,708 | 36,202,025 | 94.31 |
| 2 | ED2MTh | 33,010,502 | 31,430,746 | 95.21 |
| 3 | ED3MTh | 13,863,986 | 13,488,105 | 97.29 |
| 4 | ED4MTh | 78,595,348 | 74,094,174 | 94.27 |
| 5 | ED5MTh | 7,929,780 | 7,631,904 | 96.24 |
| 6 | ED6MMy | 30,390,858 | 28,708,450 | 94.46 |
| 7 | ED7FMy | 55,906,148 | 52,436,036 | 93.79 |
| 8 | ED8FMy | 60,748,234 | 57,164,587 | 94.10 |
| 9 | ED9MTh | 21,377,266 | 20,339,679 | 95.15 |
| 10 | ED10MTh | 67,283,706 | 64,063,859 | 95.21 |
| 11 | ED11MTh | 43,548,972 | 41,592,807 | 95.51 |
| 12 | ED12MTh | 38,572,738 | 36,868,051 | 95.58 |
| 13 | ED13MMy | 50,259,936 | 48,120,998 | 95.74 |
| 14 | ED14MMy | 14,481,504 | 13,821,923 | 95.45 |
| 15 | ED15MMy | 69,322,782 | 65,843,903 | 94.98 |
| 16 | ED16MMy | 76,065,742 | 72,686,995 | 95.56 |
| 17 | ED17FMy | 77,161,002 | 73,477,371 | 95.23 |
| 18 | ED18MMy | 71,053,386 | 67,705,141 | 95.29 |
| 19 | ED19FMy | 47,036,930 | 45,031,530 | 95.74 |
| 20 | ED31MMy | 14,481,504 | 13,821,923 | 95.45 |
| 21 | ED32FMy | 52,832,678 | 51,981,991 | 98.39 |
| 22 | ED33MMy | 58,152,960 | 57,304,405 | 98.54 |
| 23 | ED34FMy | 123,708,624 | 120,416,168 | 97.34 |
| 24 | ED35MMy | 22,040,922 | 21,341,949 | 96.83 |
| 25 | ED36FMy | 18,346,504 | 17,836,817 | 97.22 |
| 26 | ED37FMy | 15,247,530 | 14,811,099 | 97.14 |
| 27 | ED38FMy | 14,045,740 | 13,668,799 | 97.32 |
| 28 | ED39MMy | 10,031,882 | 9,490,389 | 94.6 |
| 29 | ED40MMy | 19,631,276 | 19,086,922 | 97.23 |
| 30 | ED41MMy | 17,675,214 | 17,138,674 | 96.96 |
| 31 | ED42FMy | 17,197,518 | 16,715,445 | 97.20 |
| 32 | ED43FMy | 20,253,994 | 19,762,876 | 97.58 |
| 33 | ED44MMy | 19,089,620 | 18,595,263 | 97.41 |
| 34 | ED45FMy | 15,443,812 | 15,048,366 | 97.44 |
| 35 | ED46FMy | 19,825,310 | 19,313,773 | 97.42 |
| 36 | ED47MMy | 18,482,960 | 17,955,945 | 97.15 |
| 37 | ED48FMy | 20,837,720 | 20,331,260 | 97.57 |
| 38 | ED49MMy | 28,354,756 | 27,645,334 | 97.50 |
| 39 | ED50MMy | 12,545,488 | 12,197,899 | 97.23 |
| 40 | ED51MMy | 18,414,430 | 17,887,494 | 97.14 |
| 41 | ED52MMy | 10,791,160 | 10,393,394 | 96.31 |
| 42 | ED53FMy | 15,593,224 | 15,143,281 | 97.11 |
| 43 | ED54FMy | 15,900,724 | 15,371,119 | 96.67 |
| 44 | ED55FMy | 13,516,044 | 13,120,050 | 97.07 |
| 45 | ED56MMy | 14,135,548 | 13,655,708 | 96.61 |
| 46 | ED57FMy | 14,853,118 | 14,394,121 | 96.91 |
| 47 | ED58FMy | 15,312,762 | 14,904,086 | 97.33 |
| 48 | ED59MMy | 13,069,294 | 12,596,697 | 96.38 |
| 49 | ED60MMy | 15,785,378 | 15,292,594 | 96.88 |
| 50 | ED61FMy | 14,142,550 | 13,676,914 | 96.71 |
| 51 | ED62FMy | 16,929,588 | 16,353,093 | 96.59 |
| 52 | ED63MMy | 14,165,420 | 13,668,922 | 96.50 |
| 53 | ED64MMy | 14,673,486 | 14,062,560 | 95.84 |
| 54 | ED65MMy | 17,870,538 | 17,045,288 | 95.38 |
| 55 | ED66MMy | 15,385,680 | 14,725,677 | 95.71 |
| 56 | ED67MMy | 13,587,446 | 13,018,910 | 95.82 |
| 57 | ED68MTh | 19,332,226 | 18,531,891 | 95.86 |
| 58 | ED69MTh | 10,914,414 | 10,504,874 | 96.25 |
| 59 | ED70MTh | 10,135,934 | 9,758,137 | 96.27 |
| 60 | ED71MTh | 15,638,656 | 15,027,119 | 96.09 |
| 61 | ED72MMy | 14,406,826 | 13,793,096 | 95.74 |
| 62 | ED73MMy | 14,006,880 | 13,409,371 | 95.73 |
| 63 | ED74MTh | 14,554,546 | 14,011,291 | 96.27 |
| 64 | ED75FTh | 11,192,176 | 10,761,071 | 96.15 |
| 65 | ED76FTh | 12,163,472 | 11,687,866 | 96.09 |
| 66 | ED77FTh | 15,841,642 | 15,176,469 | 95.8 |
| 67 | ED78MTh | 13,754,278 | 13,207,691 | 96.03 |
| 68 | ED79FTh | 14,255,476 | 13,739,189 | 96.38 |
| 69 | ED80FTh | 14,958,520 | 14,370,362 | 96.07 |
| 70 | ED81MTh | 17,760,240 | 17,107,600 | 96.33 |
| 71 | ED82MTh | 17,696,738 | 17,078,652 | 96.51 |
| 72 | ED83FTh | 12,109,832 | 11,648,005 | 96.19 |
| 73 | ED84FTh | 13,816,922 | 13,289,542 | 96.18 |
| 74 | ED85MTh | 17,528,610 | 16,885,972 | 96.33 |
| 75 | ED86FTh | 15,948,284 | 15,325,062 | 96.09 |
| 76 | ED87FTh | 11,354,016 | 10,889,705 | 95.91 |
| 77 | ED88FTh | 13,188,180 | 12,508,017 | 94.84 |
| 78 | ED89MTh | 14,871,958 | 14,376,656 | 96.67 |
| 79 | ED90FTh | 15,890,212 | 15,235,826 | 95.88 |
| 80 | ED91FTh | 13,877,130 | 13,337,564 | 96.11 |
| 81 | ED92FTh | 17,506,980 | 16,799,840 | 95.96 |
| 82 | ED93MTh | 13,966,186 | 13,419,096 | 96.08 |
| 83 | ED94FTh | 19,508,622 | 18,689,297 | 95.8 |
| 84 | ED95MTh | 10,017,230 | 9,701,481 | 96.85 |

**Supplementary Table 16. Statistics of genetic diversity parameters of Eld’s deer individuals, calculated using PLINK v1.9.** The observed number of homozygotes, expected number of homozygotes, number of non-missing genotypes, heterozygosity rate and genomic inbreeding coefficients are listed across 81 Eld’s deer individuals. Sampling sites: SED-ZPOT, Siamese Eld’s deer of the Zoological Park Organization of Thailand; SED-DNP, Siamese Eld’s deer of the Department of National Parks, Wildlife and Plant Conservation; BED-ZPOT, Burmese Eld’s deer of the Zoological Park Organization of Thailand; BED-DNP, Burmese Eld’s deer of the Department of National Parks, Wildlife and Plant Conservation.

| **Sample name** | **Subpopulation** | **Observed number of homozygotes** | **Expected number of homozygotes** | **Number of**  **non-missing genotypes** | **Heterozygosity rate** | **Genomic inbreeding coefficients** |
| --- | --- | --- | --- | --- | --- | --- |
| ED11MTh | SED-ZPOT | 26611 | 23880 | 33528 | 0.21 | 0.28 |
| ED12MTh | SED-ZPOT | 24421 | 23880 | 33532 | 0.27 | 0.06 |
| ED1MTh | SED-ZPOT | 27104 | 23860 | 33496 | 0.19 | 0.34 |
| ED2MTh | SED-ZPOT | 27331 | 23810 | 33431 | 0.18 | 0.37 |
| ED3MTh | SED-ZPOT | 28038 | 23170 | 32528 | 0.14 | 0.52 |
| ED4MTh | SED-ZPOT | 26125 | 23930 | 33587 | 0.22 | 0.23 |
| ED5MTh | SED-ZPOT | 26593 | 21190 | 29764 | 0.11 | 0.63 |
| ED68MTh | SED-ZPOT | 26045 | 23800 | 33405 | 0.22 | 0.23 |
| ED69MTh | SED-ZPOT | 26430 | 21370 | 29947 | 0.12 | 0.59 |
| ED70MTh | SED-ZPOT | 26748 | 22640 | 31737 | 0.16 | 0.45 |
| ED71MTh | SED-ZPOT | 26660 | 23660 | 33206 | 0.20 | 0.31 |
| ED9MTh | SED-ZPOT | 26234 | 22330 | 31373 | 0.16 | 0.43 |
| ED74MTh | SED-DNP | 27464 | 23520 | 33026 | 0.17 | 0.41 |
| ED75FTh | SED-DNP | 27314 | 23080 | 32373 | 0.16 | 0.46 |
| ED76FTh | SED-DNP | 27924 | 23320 | 32701 | 0.15 | 0.49 |
| ED77FTh | SED-DNP | 27030 | 23630 | 33156 | 0.18 | 0.36 |
| ED78MTh | SED-DNP | 28052 | 23390 | 32824 | 0.15 | 0.49 |
| ED79FTh | SED-DNP | 25718 | 23510 | 32971 | 0.22 | 0.23 |
| ED80FTh | SED-DNP | 27999 | 23490 | 32968 | 0.15 | 0.48 |
| ED81MTh | SED-DNP | 27266 | 23660 | 33211 | 0.18 | 0.38 |
| ED82MTh | SED-DNP | 27808 | 23700 | 33271 | 0.16 | 0.43 |
| ED83FTh | SED-DNP | 27871 | 23240 | 32610 | 0.15 | 0.49 |
| ED84FTh | SED-DNP | 27413 | 23440 | 32894 | 0.17 | 0.42 |
| ED85MTh | SED-DNP | 27665 | 23680 | 33229 | 0.17 | 0.42 |
| ED86FTh | SED-DNP | 27990 | 23530 | 33027 | 0.15 | 0.47 |
| ED87FTh | SED-DNP | 27733 | 22990 | 32240 | 0.14 | 0.51 |
| ED88FTh | SED-DNP | 27646 | 22160 | 31025 | 0.11 | 0.62 |
| ED89MTh | SED-DNP | 28324 | 23570 | 33084 | 0.14 | 0.50 |
| ED90FTh | SED-DNP | 28652 | 23620 | 33159 | 0.14 | 0.53 |
| ED91FTh | SED-DNP | 27600 | 23430 | 32886 | 0.16 | 0.44 |
| ED92FTh | SED-DNP | 27990 | 23710 | 33283 | 0.16 | 0.45 |
| ED93MTh | SED-DNP | 28085 | 23490 | 32968 | 0.15 | 0.48 |
| ED94FTh | SED-DNP | 27285 | 23760 | 33344 | 0.18 | 0.37 |
| ED95MTh | SED-DNP | 27564 | 22760 | 31911 | 0.14 | 0.52 |
| ED13MMy | BED-ZPOT | 21692 | 23870 | 33507 | 0.35 | –0.23 |
| ED14MMy | BED-ZPOT | 24024 | 23050 | 32347 | 0.26 | 0.10 |
| ED15MMy | BED-ZPOT | 22879 | 23910 | 33568 | 0.32 | –0.11 |
| ED16MMy | BED-ZPOT | 23445 | 23870 | 33510 | 0.30 | –0.04 |
| ED17FMy | BED-ZPOT | 24278 | 23810 | 33436 | 0.27 | 0.05 |
| ED19FMy | BED-ZPOT | 20835 | 21050 | 29564 | 0.30 | –0.03 |
| ED31MMy | BED-ZPOT | 22918 | 23100 | 32412 | 0.29 | –0.02 |
| ED32FMy | BED-ZPOT | 23651 | 23650 | 33201 | 0.29 | 0.00 |
| ED33MMy | BED-ZPOT | 23357 | 23760 | 33353 | 0.30 | –0.04 |
| ED64MMy | BED-ZPOT | 24233 | 23250 | 32628 | 0.26 | 0.10 |
| ED65MMy | BED-ZPOT | 23893 | 23480 | 32947 | 0.27 | 0.04 |
| ED66MMy | BED-ZPOT | 25310 | 23280 | 32667 | 0.23 | 0.22 |
| ED67MMy | BED-ZPOT | 25913 | 23070 | 32372 | 0.20 | 0.31 |
| ED6MMy | BED-ZPOT | 23014 | 23810 | 33436 | 0.31 | –0.08 |
| ED72MMy | BED-ZPOT | 24695 | 23350 | 32769 | 0.25 | 0.14 |
| ED73MMy | BED-ZPOT | 25861 | 22990 | 32268 | 0.20 | 0.31 |
| ED7FMy | BED-ZPOT | 23163 | 23900 | 33541 | 0.31 | –0.08 |
| ED34FMy | BED-DNP | 24117 | 23840 | 33464 | 0.28 | 0.03 |
| ED35MMy | BED-DNP | 24623 | 23550 | 33050 | 0.25 | 0.11 |
| ED36FMy | BED-DNP | 24589 | 23140 | 32470 | 0.24 | 0.16 |
| ED37FMy | BED-DNP | 24963 | 23200 | 32545 | 0.23 | 0.19 |
| ED38FMy | BED-DNP | 24038 | 21230 | 29693 | 0.19 | 0.33 |
| ED39MMy | BED-DNP | 24512 | 23670 | 33225 | 0.26 | 0.09 |
| ED40MMy | BED-DNP | 24396 | 23740 | 33319 | 0.27 | 0.07 |
| ED41MMy | BED-DNP | 24570 | 23630 | 33176 | 0.26 | 0.10 |
| ED42FMy | BED-DNP | 24570 | 23390 | 32833 | 0.25 | 0.12 |
| ED43FMy | BED-DNP | 24152 | 23740 | 33330 | 0.28 | 0.04 |
| ED44MMy | BED-DNP | 24628 | 23700 | 33265 | 0.26 | 0.10 |
| ED45FMy | BED-DNP | 24643 | 23160 | 32496 | 0.24 | 0.16 |
| ED46FMy | BED-DNP | 24684 | 23630 | 33171 | 0.26 | 0.11 |
| ED47MMy | BED-DNP | 24317 | 23510 | 33000 | 0.26 | 0.09 |
| ED48FMy | BED-DNP | 24362 | 23630 | 33176 | 0.27 | 0.08 |
| ED49MMy | BED-DNP | 23880 | 23900 | 33541 | 0.29 | 0.00 |
| ED50MMy | BED-DNP | 25174 | 23000 | 32265 | 0.22 | 0.23 |
| ED51MMy | BED-DNP | 24490 | 23440 | 32908 | 0.26 | 0.11 |
| ED52MMy | BED-DNP | 24420 | 22070 | 30927 | 0.21 | 0.27 |
| ED53FMy | BED-DNP | 24867 | 23450 | 32908 | 0.24 | 0.15 |
| ED54FMy | BED-DNP | 24460 | 23240 | 32628 | 0.25 | 0.13 |
| ED55FMy | BED-DNP | 24508 | 22720 | 31867 | 0.23 | 0.20 |
| ED56MMy | BED-DNP | 24890 | 23170 | 32513 | 0.23 | 0.18 |
| ED57FMy | BED-DNP | 24757 | 23120 | 32467 | 0.24 | 0.18 |
| ED58FMy | BED-DNP | 24011 | 23490 | 32973 | 0.27 | 0.06 |
| ED59MMy | BED-DNP | 24220 | 22870 | 32095 | 0.25 | 0.15 |
| ED60MMy | BED-DNP | 24075 | 23310 | 32732 | 0.26 | 0.08 |
| ED61FMy | BED-DNP | 24709 | 23230 | 32602 | 0.24 | 0.16 |
| ED62FMy | BED-DNP | 24251 | 23390 | 32834 | 0.26 | 0.09 |
| ED63MMy | BED-DNP | 24248 | 22680 | 31828 | 0.24 | 0.17 |

**Supplementary Table 17. Number of ROH tract lengths per class-size (Mbps) of 81 Siamese Eld’s deer (SED) and Burmese Eld’s deer (BED) individuals.** Sampling sites: SED-ZPOT, Siamese Eld’s deer of the Zoological Park Organization of Thailand; SED-DNP, Siamese Eld’s deer of the Department of National Parks, Wildlife and Plant Conservation; BED-ZPOT, Burmese Eld’s deer of the Zoological Park Organization of Thailand; BED-DNP, Burmese Eld’s deer of the Department of National Parks, Wildlife and Plant Conservation.

| **Sample name** | **Subpopulation** | **Class size (Mbps)** | | | | |
| --- | --- | --- | --- | --- | --- | --- |
|  |  | **0–6** | **6–12** | **12–24** | **24–28** | **>48** |
| ED11MTh | SED-ZPOT | 137 | 34 | 12 | 4 | - |
| ED12MTh | SED-ZPOT | 75 | 14 | 4 | 2 | - |
| ED1MTh | SED-ZPOT | 135 | 39 | 18 | 5 | - |
| ED2MTh | SED-ZPOT | 129 | 42 | 24 | 6 | 1 |
| ED3MTh | SED-ZPOT | 194 | 27 | 17 | 8 | - |
| ED4MTh | SED-ZPOT | 110 | 33 | 15 | 4 | - |
| ED5MTh | SED-ZPOT | 348 | 36 | 8 | - | - |
| ED68MTh | SED-ZPOT | 182 | 15 | 9 | 1 | - |
| ED69MTh | SED-ZPOT | 317 | 28 | 6 | 2 | - |
| ED70MTh | SED-ZPOT | 338 | 30 | 7 | - | - |
| ED71MTh | SED-ZPOT | 211 | 14 | 10 | 1 | - |
| ED9MTh | SED-ZPOT | 143 | 47 | 20 | 7 | - |
| ED74MTh | SED-DNP | 183 | 24 | 16 | 5 | - |
| ED75FTh | SED-DNP | 266 | 32 | 9 | 4 | - |
| ED76FTh | SED-DNP | 239 | 45 | 16 | 3 | - |
| ED77FTh | SED-DNP | 197 | 19 | 20 | 3 | - |
| ED78MTh | SED-DNP | 200 | 40 | 10 | 8 | 1 |
| ED79FTh | SED-DNP | 280 | 5 | - | - | - |
| ED80FTh | SED-DNP | 207 | 41 | 20 | 6 | - |
| ED81MTh | SED-DNP | 196 | 26 | 9 | 8 | - |
| ED82MTh | SED-DNP | 178 | 36 | 18 | 4 | - |
| ED83FTh | SED-DNP | 254 | 37 | 16 | 4 | - |
| ED84FTh | SED-DNP | 233 | 29 | 17 | 7 | - |
| ED85MTh | SED-DNP | 167 | 29 | 17 | 6 | 2 |
| ED86FTh | SED-DNP | 186 | 32 | 24 | 6 | - |
| ED87FTh | SED-DNP | 271 | 38 | 13 | 5 | - |
| ED88FTh | SED-DNP | 349 | 39 | 13 | 3 | - |
| ED89MTh | SED-DNP | 171 | 35 | 15 | 11 | - |
| ED90FTh | SED-DNP | 187 | 31 | 24 | 5 | - |
| ED91FTh | SED-DNP | 229 | 32 | 13 | 4 | 1 |
| ED92FTh | SED-DNP | 171 | 36 | 26 | 1 | - |
| ED93MTh | SED-DNP | 188 | 41 | 22 | 3 | 1 |
| ED94FTh | SED-DNP | 198 | 21 | 18 | 5 | - |
| ED95MTh | SED-DNP | 293 | 33 | 11 | 4 | - |
| ED13MMy | BED-ZPOT | 30 | 1 | - | - | - |
| ED14MMy | BED-ZPOT | 183 | 4 | - | - | - |
| ED15MMy | BED-ZPOT | 63 | 18 | 2 | 1 | - |
| ED16MMy | BED-ZPOT | 86 | 18 | 6 | - | - |
| ED17FMy | BED-ZPOT | 81 | 28 | 15 | 3 | - |
| ED19FMy | BED-ZPOT | 67 | 14 | 8 | - | - |
| ED31MMy | BED-ZPOT | 120 | 1 | - | - | - |
| ED32FMy | BED-ZPOT | 101 | 14 | 4 | 2 | - |
| ED33MMy | BED-ZPOT | 100 | 6 | 2 | 1 | - |
| ED64MMy | BED-ZPOT | 175 | 1 | - | - | - |
| ED65MMy | BED-ZPOT | 143 | 1 | - | - | - |
| ED66MMy | BED-ZPOT | 187 | 14 | 4 | 3 | - |
| ED67MMy | BED-ZPOT | 206 | 32 | 10 | - | - |
| ED6MMy | BED-ZPOT | 89 | 9 | - | 1 | - |
| ED72MMy | BED-ZPOT | 200 | 9 | 1 | - | - |
| ED73MMy | BED-ZPOT | 215 | 27 | 3 | - | - |
| ED7FMy | BED-ZPOT | 88 | 11 | 4 | - | - |
| ED34FMy | BED-DNP | 127 | 3 | 1 | - | - |
| ED35MMy | BED-DNP | 199 | 3 | - | - | - |
| ED36FMy | BED-DNP | 185 | 2 | - | - | - |
| ED37FMy | BED-DNP | 216 | 9 | 4 | - | - |
| ED38FMy | BED-DNP | 224 | 4 | - | - | - |
| ED39MMy | BED-DNP | 164 | 6 | 2 | - | - |
| ED40MMy | BED-DNP | 164 | 5 | - | - | - |
| ED41MMy | BED-DNP | 168 | 4 | 1 | - | - |
| ED42FMy | BED-DNP | 174 | 7 | 1 | - | - |
| ED43FMy | BED-DNP | 156 | 3 | - | - | - |
| ED44MMy | BED-DNP | 181 | 7 | - | - | - |
| ED45FMy | BED-DNP | 208 | 7 | - | - | - |
| ED46FMy | BED-DNP | 126 | 16 | 6 | - | - |
| ED47MMy | BED-DNP | 169 | 7 | 1 | - | - |
| ED48FMy | BED-DNP | 173 | 3 | - | - | - |
| ED49MMy | BED-DNP | 106 | 5 | - | - | - |
| ED50MMy | BED-DNP | 227 | 8 | 1 | - | - |
| ED51MMy | BED-DNP | 180 | 2 | 1 | - | - |
| ED52MMy | BED-DNP | 234 | 5 | - | - | - |
| ED53FMy | BED-DNP | 199 | 2 | 1 | - | - |
| ED54FMy | BED-DNP | 206 | 3 | - | - | - |
| ED55FMy | BED-DNP | 209 | 4 | 1 | - | - |
| ED56MMy | BED-DNP | 233 | 4 | - | - | - |
| ED57FMy | BED-DNP | 220 | 9 | - | - | - |
| ED58FMy | BED-DNP | 137 | 5 | 1 | - | - |
| ED59MMy | BED-DNP | 195 | 5 | - | - | - |
| ED60MMy | BED-DNP | 167 | 3 | - | - | - |
| ED61FMy | BED-DNP | 200 | 1 | 1 | - | - |
| ED62FMy | BED-DNP | 189 | 3 | 1 | - | - |
| ED63MMy | BED-DNP | 194 | 5 | - | - | - |

**Supplementary Table 18. Genomic inbreeding coefficient based on ROH (F_ROH_) of 81 Siamese Eld’s deer (SED) and Burmese Eld’s deer (BED) individuals.** Sampling sites: SED-ZPOT, Siamese Eld’s deer of the Zoological Park Organization of Thailand; SED-DNP, Siamese Eld’s deer of the Department of National Parks, Wildlife and Plant Conservation; BED-ZPOT, Burmese Eld’s deer of the Zoological Park Organization of Thailand; BED-DNP, Burmese Eld’s deer of the Department of National Parks, Wildlife and Plant Conservation.

| **Sample name** | **Subpopulation** | **F_ROH_** |
| --- | --- | --- |
| ED11MTh | SED-ZPOT | 0.38 |
| ED12MTh | SED-ZPOT | 0.15 |
| ED1MTh | SED-ZPOT | 0.47 |
| ED2MTh | BED-ZPOT | 0.50 |
| ED3MTh | SED-ZPOT | 0.50 |
| ED4MTh | SED-ZPOT | 0.37 |
| ED5MTh | SED-ZPOT | 0.46 |
| ED68MTh | SED-ZPOT | 0.26 |
| ED69MTh | SED-ZPOT | 0.42 |
| ED70MTh | SED-ZPOT | 0.40 |
| ED71MTh | SED-ZPOT | 0.30 |
| ED9MTh | SED-ZPOT | 0.52 |
| ED74MTh | SED-DNP | 0.38 |
| ED75FTh | SED-DNP | 0.44 |
| ED76FTh | SED-DNP | 0.50 |
| ED77FTh | SED-DNP | 0.38 |
| ED78MTh | SED-DNP | 0.51 |
| ED79FTh | SED-DNP | 0.22 |
| ED80FTh | SED-DNP | 0.51 |
| ED81MTh | SED-DNP | 0.40 |
| ED82MTh | SED-DNP | 0.46 |
| ED83FTh | SED-DNP | 0.50 |
| ED84FTh | SED-DNP | 0.45 |
| ED85MTh | SED-DNP | 0.46 |
| ED86FTh | SED-DNP | 0.51 |
| ED87FTh | SED-DNP | 0.50 |
| ED88FTh | SED-DNP | 0.56 |
| ED89MTh | SED-DNP | 0.51 |
| ED90FTh | SED-DNP | 0.52 |
| ED91FTh | SED-DNP | 0.45 |
| ED92FTh | SED-DNP | 0.49 |
| ED93MTh | SED-DNP | 0.52 |
| ED94FTh | SED-DNP | 0.41 |
| ED95MTh | SED-DNP | 0.49 |
| ED7FMy | BED-ZPOT | 0.14 |
| ED13MMy | BED-ZPOT | 0.02 |
| ED14MMy | BED-ZPOT | 0.14 |
| ED15MMy | BED-ZPOT | 0.13 |
| ED16MMy | BED-ZPOT | 0.17 |
| ED17FMy | BED-ZPOT | 0.31 |
| ED19FMy | BED-ZPOT | 0.16 |
| ED31MMy | SED-ZPOT | 0.11 |
| ED32FMy | BED-ZPOT | 0.18 |
| ED33MMy | BED-ZPOT | 0.11 |
| ED64MMy | BED-ZPOT | 0.12 |
| ED65MMy | BED-ZPOT | 0.09 |
| ED66MMy | BED-ZPOT | 0.25 |
| ED67MMy | BED-ZPOT | 0.33 |
| ED6MMy | BED-ZPOT | 0.11 |
| ED72MMy | BED-ZPOT | 0.17 |
| ED73MMy | BED-ZPOT | 0.30 |
| ED34FMy | BED-DNP | 0.09 |
| ED35MMy | BED-DNP | 0.16 |
| ED36FMy | BED-DNP | 0.16 |
| ED37FMy | BED-DNP | 0.22 |
| ED38FMy | BED-DNP | 0.18 |
| ED39MMy | BED-DNP | 0.14 |
| ED40MMy | BED-DNP | 0.13 |
| ED41MMy | BED-DNP | 0.14 |
| ED42FMy | BED-DNP | 0.16 |
| ED43FMy | BED-DNP | 0.12 |
| ED44MMy | BED-DNP | 0.15 |
| ED45FMy | BED-DNP | 0.18 |
| ED46FMy | BED-DNP | 0.21 |
| ED47MMy | BED-DNP | 0.14 |
| ED48FMy | BED-DNP | 0.13 |
| ED49MMy | BED-DNP | 0.09 |
| ED50MMy | BED-DNP | 0.21 |
| ED51MMy | BED-DNP | 0.14 |
| ED52MMy | BED-DNP | 0.19 |
| ED53FMy | BED-DNP | 0.17 |
| ED54FMy | BED-DNP | 0.17 |
| ED55FMy | BED-DNP | 0.17 |
| ED56MMy | BED-DNP | 0.18 |
| ED57FMy | BED-DNP | 0.19 |
| ED58FMy | BED-DNP | 0.12 |
| ED59MMy | BED-DNP | 0.16 |
| ED60MMy | BED-DNP | 0.13 |
| ED61FMy | BED-DNP | 0.17 |
| ED62FMy | BED-DNP | 0.16 |
| ED63MMy | BED-DNP | 0.15 |

### References

1 Vurture, G. W. *et al.* GenomeScope: fast reference-free genome profiling from short reads. *Bioinformatics* **33**, 2202−2204 (2017).

2 Andrews, S. FastQC: a quality control tool for high throughput sequence data. http://www.bioinformatics.babraham.ac.uk/projects/fastqc. (2010).

3 Brown, D. & Morgenstern B. *Algorithms in Bioinformatics*. 14th International Workshop, WABI 2014, Wroclaw, Poland, September 8–10, 2014. Proceedings (Springer, 2014).

4 Koren, S. *et al.* Canu: scalable and accurate long-read assembly via adaptive k-mer weighting and repeat separation. *Genome Res.* **27**, 722−736 (2017).

5 Boetzer, M. & Pirovano, W. SSPACE-LongRead: scaffolding bacterial draft genomes using long read sequence information. *BMC Bioinformatics* **15**, 1−9 (2014).

6 Chakraborty, M., Baldwin-Brown, J. G., Long, A. D. & Emerson, J. Contiguous and accurate *de novo* assembly of metazoan genomes with modest long read coverage. *Nucleic Acids Res.* **44**, e147 (2016).

7 Walker, B. J. *et al.* Pilon: an integrated tool for comprehensive microbial variant detection and genome assembly improvement. *PLoS One* **9**, e112963 (2014).

8 Krueger, F. Trim galore: a wrapper tool around Cutadapt and FastQC to consistently apply quality and adapter trimming to FastQ files, with some extra functionality for MspI-digested RRBS-type (Reduced Representation Bisufite-Seq) libraries. http://www.bioinformatics.babraham.ac.uk/projects/trim_galore/. (2015).

9 Simpson, J. T. *et al.* ABySS: a parallel assembler for short read sequence data. *Genome Res.* **19**, 1117−1123 (2009).

10 Alonge, M. *et al.* RaGOO: fast and accurate reference-guided scaffolding of draft genomes. *Genome Biol.* **20**, 224 (2019).

11 Smit, A., Hubley, R. & Green, P. RepeatMasker Open-4.0. https://www.repeatmasker.org/ (2015).

12 Cantarel, B. L. *et al.* MAKER: an easy-to-use annotation pipeline designed for emerging model organism genomes. *Genome Res.* **18**, 188−196 (2008).

13 Altschul, S. F. *et al.* Gapped BLAST and PSI-BLAST: a new generation of protein database search programs. *Nucleic Acids Res.* **25**, 3389−3402 (1997).

14 Arias-Carrasco, R., Vásquez-Morán, Y., Nakaya, H. I. & Maracaja-Coutinho, V. StructRNAfinder: an automated pipeline and web server for RNA families prediction. *BMC Bioinformatics* **19**, 55 (2018).

15 Nawrocki, E. P. & Eddy, S. R. Infernal 1.1: 100-fold faster RNA homology searches. *Bioinformatics* **29**, 2933−2935 (2013).

16 Lorenz, R. *et al.* ViennaRNA Package 2.0. *Algorithms Mol. Biol.* **6**, 26 (2011).

17 Nawrocki, E. P. *et al.* Rfam 12.0: updates to the RNA families database. *Nucleic Acids Res.* **43**, 130−137 (2015).

18 Lowe, T. M. & Eddy, S. R. tRNAscan-SE: a program for improved detection of transfer RNA genes in genomic sequence. *Nucleic Acids Res.* **25**, 955−964 (1997).

19 Dierckxsens, N., Mardulyn, P. & Smits, G. NOVOPlasty: *de novo* assembly of organelle genomes from whole genome data. *Nucleic Acids Res.* **45**, e18 (2017).

20 Bernt, M. *et al.* MITOS: improved *de novo* metazoan mitochondrial genome annotation. *Mol. Phylogenet. Evol.* **69**, 313−319 (2013).

21 Altschul, S. F., Gish, W., Miller, W., Myers, E. W. & Lipman, D. J. Basic local alignment search tool. *J. Mol. Biol.* **215**, 403−410 (1990).

22 Greiner, S., Lehwark, P. & Bock, R. OrganellarGenomeDRAW (OGDRAW) version 1.3. 1: expanded toolkit for the graphical visualization of organellar genomes. *Nucleic Acids Res.* **47**, W59−W64 (2019).

23 Letunic, I. & Bork, P. Interactive Tree Of Life (iTOL) v5: an online tool for phylogenetic tree display and annotation. *Nucleic Acids Res.* **49**, W293−W296 (2021).

24 Emms, D. M. & Kelly, S. OrthoFinder: phylogenetic orthology inference for comparative genomics. *Genome Biol.* **20**, 238 (2019).

25 Capella-Gutiérrez, S., Silla-Martínez, J. M. & Gabaldón, T. trimAl: a tool for automated alignment trimming in large-scale phylogenetic analyses. *Bioinformatics* **25**, 1972−1973 (2009).

26 Darriba, D. *et al.* ModelTest-NG: a new and scalable tool for the selection of DNA and protein evolutionary models. *Mol. Biol. Evol.* **37**, 291−294 (2020).

27 Camacho, C. *et al.* BLAST+: architecture and applications. *BMC Bioinformatics* **10**, 421 (2009).

28 Overbeek, R., Fonstein, M., D’Souza, M., Pusch, G. D. & Maltsev, N. The use of gene clusters to infer functional coupling. *Proc. Natl. Acad. Sci. U.S.A.* **96**, 2896−2901 (1999).

29 Larkin, M. A. *et al.* Clustal W and Clustal X version 2.0. *Bioinformatics* **23**, 2947−2948 (2007).

30 Liu, K., Linder, C. R. & Warnow, T. Multiple sequence alignment: a major challenge to large-scale phylogenetics. *PLoS Curr.* **2**, RRN1198 (2010).

31 Felsenstein, J. PHYLIP (Phylogeny Inference Package) version 3.6 http://www.evolution.gs.washington.edu/phylip.html. (2004).

32 Loytynoja, A. & Goldman, N. Phylogeny-aware gap placement prevents errors in sequence alignment and evolutionary analysis. *Science* **320**, 1632−1635 (2008).

33 Langmead, B. & Salzberg, S. L. Fast gapped-read alignment with Bowtie 2. *Nat. Methods* **9**, 357−359 (2012).

34 Li, H. *et al.* The sequence alignment/map format and SAMtools. *Bioinformatics* **25**, 2078−2079 (2009).

35 Anderson, C. A. *et al.* Data quality control in genetic case-control association studies. *Nat. Protoc.* **5**, 1564−1573 (2010).

36 Gazal, S. *et al.* Inbreeding coefficient estimation with dense SNP data: comparison of strategies and application to HapMap III. *Hum. Hered.* **77**, 49−62 (2014).
